# Smoking inequalities among culturally diverse populations in Australia: A secondary dataset analysis of the HILDA survey 2002–2019

**DOI:** 10.18332/tid/217085

**Published:** 2026-06-30

**Authors:** Lin Aung, Karen Block, Humaira Maheen, Upul Cooray, Ankur Singh

**Affiliations:** 1 The University of Sydney School of Dentistry, The University of Sydney, Sydney, Australia; 2 Charles Perkins Centre, University of Sydney, Sydney, Australia; 3 Nossal Institute for Global Health, Melbourne School of Population and Global Health, University of Melbourne, Melbourne, Australia; 4 Centre for Health Policy, Melbourne School of Population and Global Health, University of Melbourne, Australia

**Keywords:** tobacco smoking, smoking prevalence, inequalities, IPW, smoking trend

## Abstract

**INTRODUCTION:**

Given that over 7 million Australians are born overseas, this study aimed to examine smoking prevalence, trends, and inequalities across major cultural subpopulations in Australia from 2002 to 2019, using a causal weighting method.

**METHODS:**

This was a secondary analysis of repeated cross-sectional data from 18 waves (2002–2019) of the Household, Income and Labour Dynamics in Australia (HILDA) survey. Respondents aged ≥15 years with smoking status and region of birth were grouped into six cultural subpopulations. Inverse probability of treatment weighting (IPTW) was applied to adjust for sociodemographic characteristics across six cultural subpopulations. Inequalities in never, current, and ex-smokers were assessed using prevalence differences and ratios, stratified by sex, with Australian-born individuals as the reference group.

**RESULTS:**

Absolute differences (per 100 people) in never smoking prevalence between the group with the poorest smoking prevalence and the Australian-born group, decreased from -16.53% (95% CI: -18.17 – -14.89) to -10.84% (95% CI: -12.24 – -9.43), and from -7.56% (95% CI: -11.88 – -3.24) to -4.01% (95% CI: -8.16–0.14) in ex-smoking prevalence. However, absolute differences in current smoking increased from 3.49% (95% CI: -0.70–7.68) to 9.09% (95% CI: 5.52–12.66). Men from the Middle East and Africa and women from Europe, the Middle East, and Africa had the poorest outcomes, with increasing current smoking and declining never smoking prevalence.

**CONCLUSIONS:**

While inequalities in never and ex-smoking prevalence have narrowed, inequalities in current smoking persist or have worsened among specific subgroups. These findings indicate persistent smoking inequalities among certain immigrant subgroups, suggesting the need for further research to understand better the social, cultural, and structural factors that may underlie these patterns and guide future equity-focused tobacco control strategies.

## INTRODUCTION

Tobacco smoking is a significant public health issue worldwide. Cigarette smoking is the predominant form of tobacco use globally among various types of tobacco products, including cigars, waterpipe tobacco, pipe tobacco, cigarillos, roll-your-own tobacco, heated tobacco, bidis and kreteks, and smokeless tobacco^1^. All types of tobacco pose health risks, and even minimal exposure to secondhand smoke is unsafe^1,2^. Smoking can damage nearly every part of the body, leading to life-threatening diseases such as lung cancer, chronic respiratory disease, heart disease, and stroke^3^. Smoking during pregnancy also increases the risk of poor fetal outcomes like growth restriction, stillbirth, preterm birth, and potential behavioral disorders in childhood^4^. In Australia, smokers have a threefold higher risk of death compared to non-smokers^5^.

Globally, smoking prevalence has decreased since 1990, but population growth has increased the total number of smokers^6^. According to the World Health Organization (WHO), the global prevalence of any tobacco use among individuals aged ≥15 years declined from 26.2% in 2010 to 19.5% in 2024, and is projected to further decrease to 17.4% by 2030^7^. However, over 7 million people die annually from tobacco use, with 1.6 million from secondhand smoke^8^. In Australia, smoking prevalence has significantly decreased from 16.1% in 2011–2012 to 10.1% in 2021–2022^9^.

Smoking behaviors are influenced by numerous sociodemographic and socioeconomic factors, including income, education level, employment, ethnicity, cultural and family characteristics, acculturation, social marginalization, tobacco industry influence, and insufficient tobacco control policies^10,11^. In Australia, higher smoking prevalence is seen among lower socioeconomic groups, regional populations, people with mental disorders, and people with disabilities^12,13^. Furthermore, smoking prevalence varies across cultural subgroups^14^, highlighting the role of cultural background.

Australia is one of the most multicultural nations in the world. In 2021, about 7 million people in Australia (27.6%) were born overseas, and 5.8 million (22.8%) speak a language other than English at home^15^. Individuals from non-English speaking countries or who do not speak English at home are referred to as Culturally and Linguistically Diverse (CALD) populations – a widely used term in Australian research^16^. CALD populations face notable health inequalities, with certain groups experiencing higher rates of poor physical and mental health outcomes^17^.

Despite the overall decrease in smoking prevalence in Australia, studies comparing immigrants with those Australian-born have not provided consistent results. In 2011, a cross-sectional study conducted in the state of New South Wales, Australia, suggested that smoking among immigrants varied by their region of origin, with immigrants from Europe, North Africa, and the Middle East having higher odds of smoking than those Australian-born, while women from the Middle East and Southeast Asia were less likely to smoke than Australian-born women^14^. More recently, a longitudinal study in 2018 found that immigrants from non-English-speaking countries initially exhibit lower smoking rates upon arrival in Australia. Still, this trend diminished over time, with immigrants who arrived as children or adolescents and resided in Australia for more than 20 years demonstrating higher smoking rates compared to those Australian-born^18^. Another cross-sectional study in 2020 reported that people from CALD backgrounds are more likely to support policies against drug and tobacco use compared to those born in Australia, the United Kingdom, or New Zealand, who only speak English at home, but there was no significant difference between the two groups when it comes to smoking^19^. However, in 2021–2022, the Australian Bureau of Statistics reported that Australian-born adults were more likely to be daily smokers than those born overseas (11.1% vs 8.1%)^9^. Additionally, individuals who speak English at home are more likely to smoke daily than those who speak other languages (10.6% vs 6.9%)^9^. This contrasts with the global trend of higher smoking prevalence among immigrants^20^.

Despite Australia’s cultural diversity, research on smoking trends among CALD subpopulations remains limited. As this population grows, it is crucial to understand how their smoking patterns differ from those of the general population. This study addresses this gap by examining smoking prevalence trends among six subpopulations classified by region of origin and comparing them with the Australian-born population. It analyzes similarities, differences, and inequalities over time, focusing on the three smoking outcomes: never, current, and ex-smoking.

## METHODS

This was a secondary dataset analysis of repeated cross-sectional data from 18 waves (2002–2019) of the Household, Income and Labour Dynamics in Australia (HILDA) survey.

### Study population and data collection

The HILDA survey is an annual household panel study that follows a nationally representative sample of over 17000 Australians aged ≥15 years and collects data on demographics, health, income, employment, and wellbeing^21^. The sample was refreshed in 2011 to maintain representativeness^21^. Data were collected via face-to-face interviews, self-completed questionnaires, and telephone interviews.

In this analysis, we included respondents aged ≥15 years with non-missing smoking status and region of birth. We categorized them into six cultural subpopulations based on their country of birth, using the Standard Australian Classification of Countries (SACC) Level 1^22^. This study follows the categorization established in a previous publication^23^ and included the following regions: Australia; English-speaking countries (ESC), which include the United Kingdom, Ireland, the United States, New Zealand, South Africa, and Canada; Europe (excluding English-speaking countries); Asia, which includes Southeast Asia, Northeast Asia, Southern and Central Asia; Middle East and Africa, which includes the Middle East, North Africa, and Sub-Saharan Africa (excluding South Africa); Oceania and America (excluding North America)

These categories, based on the regions of birth, serve as a proxy for culturally and linguistically diverse status. It is acknowledged, however, that cultural diversity encompasses factors beyond the country of birth.

### Outcome variable: Smoking

At each survey wave, participants were asked: ‘Do you smoke cigarettes or any other tobacco product?’. Response options were: ‘No, I have never smoked’, ‘No, I no longer smoke’, ‘Yes, I smoke daily’, ‘Yes, I smoke at least weekly’, and ‘Yes, I smoke less often than weekly’. Individuals who responded that they were current smokers, regardless of the frequency, were classified as smokers, those who had never smoked were classified as never smokers, and those who responded ‘I no longer smoke’ were classified as ex-smokers.

### Covariates

Education status was categorized into three groups: Year 12 or lower, Diploma or Certificate, and Bachelor’s degree or higher. Residence (urban, rural) and sex (male, female) were analyzed as binary variables, while age and equalized household income were treated as continuous variables.

### Statistical analysis

The initial step was to quantify and describe smoking prevalence over time within six culturally diverse population groups. Due to the varying sizes and characteristics of these groups, inverse probability of treatment weighting (IPTW) was employed to address potential data imbalance and bias and ensure comparability^24^. This method creates a pseudo-population that balances covariates across groups, allowing valid comparisons of smoking prevalence between the six groups and thereby minimizing bias in our estimates.

To achieve this, a multinomial logistic regression model was first used to predict the probability that each individual belongs to their respective population subgroup, based on covariates age, sex, education level, household income, and residence. The inverse of the predicted probabilities was used as the inverse probability weight (IPW), which was trimmed at the top and bottom 1% to exclude extreme values. The IPWs were then rescaled to match the original overall weight and used to estimate smoking prevalence and its 95% confidence interval (95% CI). This process was repeated for each study wave of data, incorporating survey weights. Next, linear regression models were fitted to the IPW-adjusted data to estimate trend lines for current, ex-, and never smoking statuses.

Finally, smoking inequalities were examined by estimating absolute and relative differences in prevalence, with 95% CIs. Absolute differences were estimated by subtracting the Australian-born population’s prevalence (reference group) from each group’s prevalence. Relative differences were obtained by dividing each group’s prevalence by that of the Australian-born group. Then, an inequality plot was employed, in which all three measures can be visualized simultaneously: the smoking prevalence for the reference group (Australian) is plotted on the x-axis, and the prevalence difference (absolute inequality) on the y-axis. Contour lines represent the prevalence ratio (relative inequality). This method of visualization has been applied in previous studies^13^. Data analysis was conducted in R Studio (R version 4.4.2, 2024-10-31;^25^ Platform: aarch64-apple-darwin20).

## RESULTS

### Characteristics of the sample

Table 1 shows group characteristics for baseline (2002) and final wave (2019). About 12000 individuals responded in each wave, with slightly more females, especially in the Asia-born group. In 2002, less than half of participants had an education level higher than Year 12, rising to about two-thirds by 2019. In 2002, the mean age was around 41 years for most groups, except ESC and Europe, with mean ages of 50 and 55 years, respectively. By 2019, the mean age increased to about 45 years for all groups, while ESC and Europe reached mean ages of 60 years. Generally, unadjusted current smoking decreased, while the prevalence of never and ex-smokers increased across all groups.

**Table 1:** Characteristics* of study participants by region of birth (Baseline Year: 2002, Final Year: 2019)

*Characteristics*	*2002*						*2019*					
	*Australia*	*English* *speaking* *countries*	*Europe* *(excluding* *ES* *countries)*	*Asia*	*Middle* *East,* *African* *countries*	*Oceania* *and* *America*	*Australia*	*English* *speaking* *countries*	*Europe* *(excluding* *ES* *countries)*	*Asia*	*Middle* *East,* *African* *countries*	*Oceania* *and* *America*
**Total,** n	9270	1275	638	542	149	126	12719	1405	524	867	184	203
**Age** (years)	41 (29–55)	49 (38–62)	55 (44–65)	40 (28–49)	40 (33–50)	36 (29–45)	43 (28–59)	57 (44–70)	66 (51–74)	45 (34–59)	55 (36–62)	46 (35–56)
**Sex**												
Male	4355 (47)	643 (50)	307 (48)	232 (43)	79 (53)	62 (49)	5958 (47)	706 (50)	243 (46)	354 (41)	99 (54)	102 (50)
Female	4915 (53)	632 (50)	331 (52)	310 (57)	70 (47)	64 (51)	6761 (53)	699 (50)	281 (54)	513 (59)	85 (46)	101 (50)
**Education level**												
Bachelor’s degree or higher	1222 (13)	207 (16)	84 (13)	162 (30)	34 (23)	24 (19)	2503 (20)	311 (22)	135 (26)	430 (50)	60 (33)	58 (29)
Diploma or Certificate	2780 (30)	484 (38)	210 (33)	132 (24)	39 (26)	35 (28)	5035 (40)	616 (44)	198 (38)	236 (27)	59 (32)	91 (45)
Year 12 or lower	5268 (57)	584 (46)	344 (54)	248 (46)	76 (51)	67 (53)	5181 (41)	478 (34)	191 (36)	201 (23)	65 (35)	54 (27)
**Equalized income** (A$)	30721 (18296–46959)	31272 (17637–48233)	23640 (13651–40641)	28999 (17329–43748)	28056 (14118–42333)	26667 (16400–44250)	59800 (36947–89644)	59733 (35548–93333)	40778 (25540–74548)	63823 (40000–92901)	48400 (27361–81267)	58433 (38120–87082)
**Residence**												
Urban	7804 (84)	1127 (88)	576 (90)	528 (97)	139 (93)	124 (98)	10887 (86)	1218 (87)	476 (91)	824 (95)	175 (95)	189 (93)
Rural	1466 (16)	148 (12)	62 (9.7)	14 (2.6)	10 (6.7)	2 (1.6)	1832 (14)	187 (13)	48 (9.2)	43 (5.0	9 (4.9)	14 (6.9)
**Smoking**												
Never smoke	4540 (49)	512 (40)	245 (38)	385 (71)	87 (58)	71 (56)	7235 (57)	646 (46)	244 (47)	653 (75)	105 (57)	118 (58)
Ex-smoke	2392 (26)	486 (38)	256 (40)	80 (15)	27 (18)	19 (15)	3319 (26)	561 (40)	215 (41)	144 (17)	34 (18)	52 (26)
Current smoke	2338 (25)	277 (22)	137 (21)	77 (14)	35 (23)	36 (29)	2165 (17)	198 (14)	65 (12)	70 (8.1)	45 (24)	33 (16)

* Values are presented as n (%) for categorical variables and median (IQR) for continuous variables. All descriptive statistics are unweighted. Equivalized household income was calculated using the modified OECD equivalence scale (1.0 for the first adult, 0.5 for each additional adult, and 0.3 for each child). Data source: Household, Income and Labour Dynamics in Australia (HILDA) Survey. ES: English speaking. IQR: interquartile range. A$: 1000 Australian Dollars about US$710.

Supplementary file Table 1 shows the prevalence and inequalities of smoking (never, current, and ex-smoking) by subpopulation for men and women, along with 95% CI and p-value for linear trend.

### Never smoking

**Figure 1:**
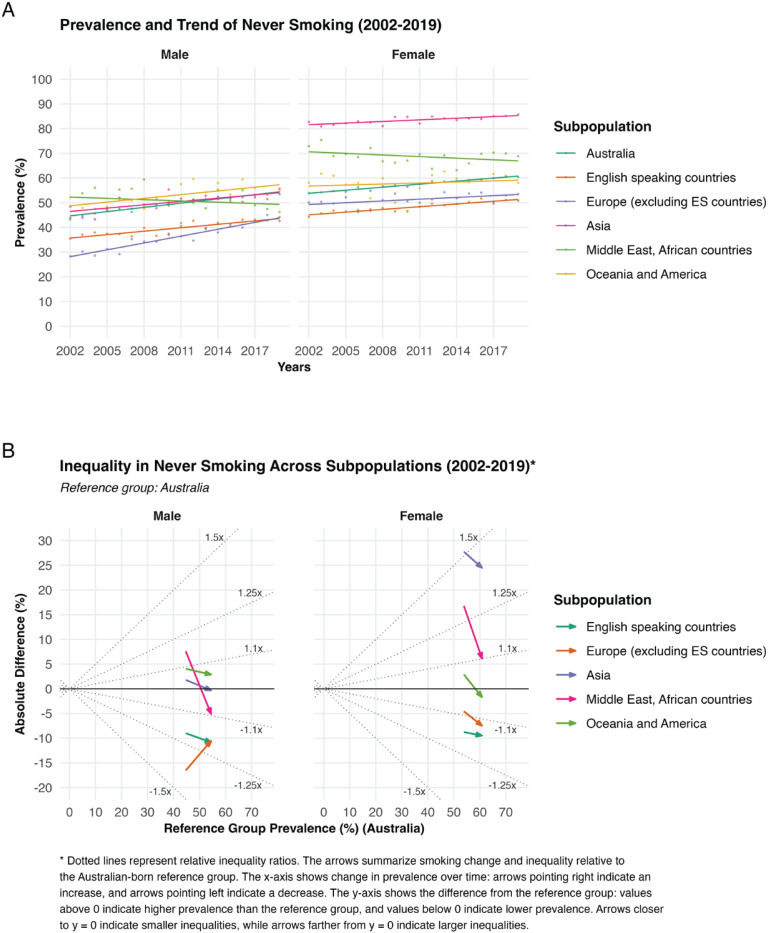
Never smoking trends and inequalities across subpopulations of Australia, 2009–2021

Figure 1A illustrates yearly trends in never smoking prevalence by sex. Apart from Middle East- and African-born individuals, all groups showed an improving trend in never smoking. Among men, all groups except those who were Oceania- and America-born had lower prevalence than Australian-born men by the end of the study period. Among women, Oceania- and America-born women followed a similar trend to Australian-born women. ESC-born and Europe-born women had lower prevalence than Australian-born women, while Asia-born women had higher prevalence of never smoking. Although Middle East- and African-born men and women had higher never smoking prevalence than Australian-born at baseline, this declined over time, with men falling below Australian-born men by the end of the study period.

Figure 1B illustrates never smoking inequalities. Among men, the prevalence differences in never smoking narrowed over time, suggesting reduced inequality. However, the prevalence differences are below those of the reference group, indicating poorer outcomes than those of Australian-born men. Among women, the differences also narrowed but remained dispersed, indicating reduced but high inequalities – mainly due to consistently higher never smoking prevalence among Asia-born women.

Among men, inequalities in never smoking between Australian-born individuals and the group with the poorest smoking prevalence declined from -16.5% (95% CI: -18.2 – -14.9) to -10.8% (95% CI: -12.2 – -9.4) on the absolute scale, and from 0.63 (95% CI: 0.60–0.67) (approximately 1.4-fold) to 0.80 (95% CI: 0.78–0.83) (about 1.2-fold) on the relative scale over the study period. Among women, inequalities increased from -8.8 (95% CI: -9.7 – -7.9) to -9.5 (95% CI: -10.4 – -8.6) on the absolute scale, while remaining stable on the relative scale at approximately 1.2-fold, from 0.83 (95% CI: 0.82–0.85) to 0.84 (95% CI: 0.83–0.86).

### Current smoking

**Figure 2:**
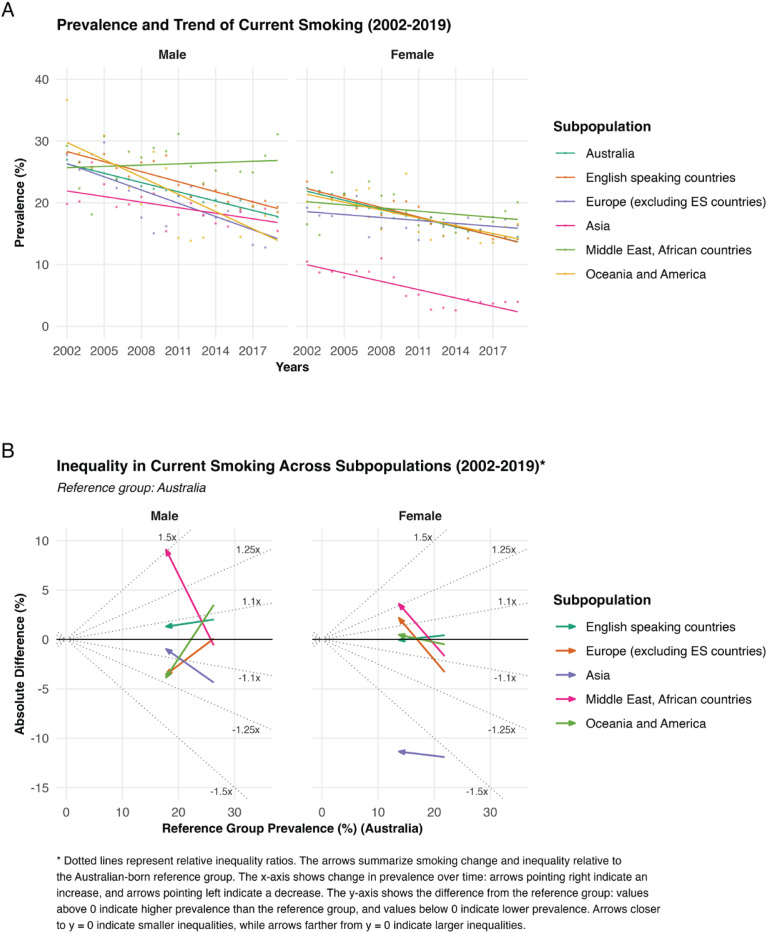
Current smoking trends and inequalities across subpopulations of Australia, 2009–2021

Figure 2A shows a decline in current smoking prevalence across all subpopulations for both men and women, except Middle East- and African-born men. Among men, those born in ESC and the Middle East and Africa had higher smoking prevalence than Australia-born men. In contrast, those from Europe, Asia, Oceania, and the Americas had lower prevalence. Middle East and Africa-born men showed a slight increase over time. Among women, Middle East- and African-born and European-born women had higher current smoking prevalence than Australian-born women. Oceania- and America-born women and those from ESC had prevalence rates similar to those of Australian-born women, while Asian-born women consistently had lower prevalence throughout the study period.

Figure 2B shows current smoking inequalities. Among men, widening prevalence differences over time indicate persistent or worsening inequalities in current smoking. Among women, except those born in Asia, prevalence differences narrowed but clustered slightly above Australian-born women, suggesting narrowing inequalities but a poorer current smoking outcome than Australian-born women.

Among men, inequalities in current smoking between Australian-born individuals and the group with the poorest smoking prevalence increased from 3.50% (95% CI: -0.70–7.68) to 9.09% (95% CI: 5.52–12.66) on the absolute scale, and from 1.13 (95% CI: 0.98–1.30) (approximately 1.1-fold) to 1.51 (95% CI: 1.32–1.73) (about 1.5-fold) on the relative scale, over the study period. Among women, inequalities also increased, rising from 0.42% (95% CI: -0.80–1.65) to 3.6% (95% CI: 0.83–6.38) on the absolute scale, and from 1.02 (95% CI: 0.96–1.08) (almost equality) to 1.26 (95% CI: 1.07–1.49) on the relative scale.

### Ex-smoking

**Figure 3:**
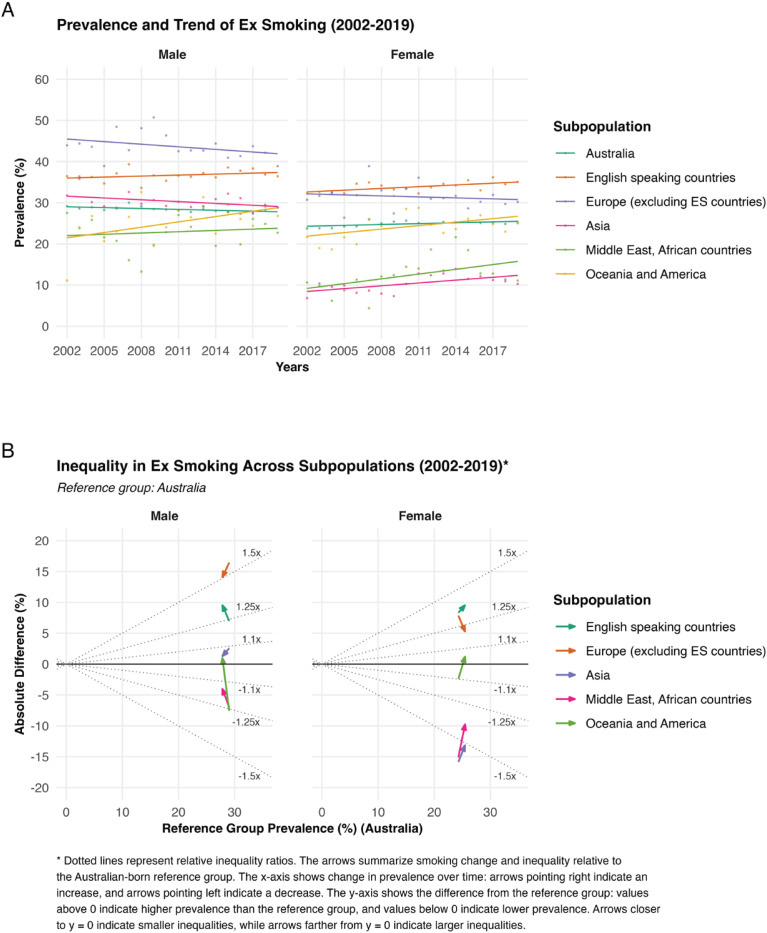
Ex-smoking trends and inequalities across subpopulations of Australia, 2009–2021

Figure 3A shows yearly trends in ex-smoking prevalence by sex. Among men, ESC-born, Asia-born, and Europe-born individuals had higher ex-smoking prevalence than Australia-born men, while Middle East- and African-born men had lower prevalence. Oceania- and America-born men increased gradually over time and were similar to Australian-born men by the end of the study period. Among women, ESC-born and Europe-born had stable trends but remained better than Australia-born women. In contrast, Middle East/Africa-born and Asia-born women showed improving trends, though still below Australia-born women. Oceania/America-born women had similar trends to Australia-born women.

Figure 3B illustrates smoking inequalities. Among men, the prevalence differences showed a gradual narrowing over the years; this pattern suggests that inequalities in ex-smoking decreased between the groups. Additionally, the prevalence differences appear to be clustered above the Australia-born reference group. Similarly, among women, the pattern also suggests reducing inequalities in ex-smoking, and the trend lines are converging towards the reference group of Australia.

Among men, inequalities in ex-smoking between Australian-born individuals and the group with the poorest smoking prevalence declined from -7.56% (95% CI: -11.88 – -3.24) to -4.01% (95% CI: -8.16–0.14] on the absolute scale, and from 0.74 (95% CI: 0.60–0.90) (approximately 1.3-fold) to 0.86 (95% CI: 0.72–1.02) (about 1.1-fold) on the relative scale over the study period. Among women, inequalities also decreased, from -15.85% (95% CI: -17.56 – -14.15) to -13.15% (95% CI: -14.85 – -11.45) on the absolute scale, and from 0.35 (95% CI: 0.29–0.42) (about 1.6-fold) to 0.48 (95% CI: 0.42–0.55) (about 1.5–fold) on the relative scale.

## DISCUSSION

Over the 18 years (2002–2019), Australia experienced overall improvements in smoking trends; however, not all subpopulations benefited equally. Women had more favorable outcomes than men, with lower current smoking and higher never smoking prevalences. Men from ESC, and particularly those from the Middle East and Africa, had worse outcomes than Australian-born men. Similarly, women from Europe, the Middle East, and Africa experienced poorer smoking outcomes than Australian-born women. These persistent variations indicate the smoking inequalities across subpopulations.

To our knowledge, no literature clearly defines how large the difference in smoking prevalence needs to be to indicate significant smoking inequalities and public health implications. However, due to the substantial health risks associated with smoking^26^, even slight differences in smoking prevalence may have significant public health implications.

While smoking outcomes have improved over the past two decades, inequalities persist across different demographic groups in Australia. Our findings suggest that inequalities in never smoking and ex-smoking have narrowed over time. However, inequalities between groups in current smoking remain largely unchanged or have worsened. This uneven pattern of inequality reduction may be partially attributed to selective migration^27^, in which individuals who migrate tend to be healthier, more educated, or of higher socioeconomic status, which may contribute to lower smoking prevalence in some groups.

Gender is one factor that influences the differences in these smoking trends. Women generally have better smoking prevalence (lower current smoking and higher never smoking) compared to men. Furthermore, the prevalence of smoking in countries where immigrants come from, can also play an essential role in these differences^20^. The immigrants’ countries of origin and Australia are at different stages of the ‘smoking epidemic’^28^. Additionally, smoking habits tend to pass down from one generation to the next in a recurring pattern influenced by societal norms, familiarity, and addiction^29^. For example, young people who are exposed to smoking behavior are more likely to try smoking, and once addicted, it is harder for them to quit. A study from the US and Canada found that smoking initiation among young adults is associated with alcohol and illegal drug use, as well as exposure to social norms and perceptions that favor smoking^30^. The rise of e-cigarette (vaping) use in Australia^31^ may contribute to slow improvement in smoking prevalence among young adults, as e-cigarette use has been associated with higher odds of subsequent cigarette use^32^. Furthermore, immigration challenges such as language barriers, adjusting to a new culture, discrimination, and other uncertainties associated with migrating to a new country may be associated with immigrants using smoking as a tool to deal with these stresses. To better understand these inequalities in smoking outcomes, we need more research (both quantitative and qualitative) to look at how immigrants start and stop smoking over time.

It is important to address the specific drivers of smoking initiation and tobacco addiction in certain subpopulations. These inequalities in smoking outcomes could exacerbate existing health and social inequalities^29^. For example, groups with poor smoking outcomes are at greater risk for smoking-related diseases such as lung cancer, heart disease, and respiratory disorders. In addition, the inequalities in smoking also reflect social inequities, including access to education, employment opportunities, and healthcare services. Future analyses should consider socioeconomic status and education, particularly among immigrants from regions like the Middle East and Africa, to understand their impact on smoking behaviors and inequalities.

The overall smoking prevalence in Australia improved from 2002 to 2019; however, persistent inequalities across subpopulations suggest that future public health efforts may need to consider how improvements in smoking prevalence are achieved equitably. Applying proportionate universalism^33^, where interventions are universally available but support is tailored to levels of need, may help reduce these gaps. Additionally, the prevalence differences between men and women suggest that strategies may also need to consider sex-related factors.

### Strengths and limitations

The strength of the study is that the HILDA survey is a nationally representative, household-based panel study, and we used IPW to address imbalances in group sizes and characteristics, enhancing comparability and the precision of smoking prevalence estimates. This makes the findings generalizable to the broader Australian population and provides new evidence on population-subgroup-specific smoking prevalence and trends over the past decade.

This study also has limitations. Despite the use of IPTW, unmeasured confounding may remain. In addition, as this is a repeated cross-sectional, descriptive analysis, the findings are not causal and should not be interpreted as evidence of a causal link between cultural background and smoking outcomes. Smoking status was self-reported without biochemical validation, and the survey question did not distinguish tobacco product types (e.g. cigarettes vs other tobacco products), which may introduce information bias through misclassification if reporting or product preferences vary across cultural groups. Finally, subpopulations were categorized by region of birth, and due to small sample sizes, some groups were combined; this may not fully capture within-group diversity and reduces granularity, potentially obscuring distinct smoking patterns.

## CONCLUSIONS

Between 2002 and 2019, all subpopulations in Australia experienced declines in current smoking, increases in never smoking, and ex-smoking prevalence. Inequalities in current smoking persisted despite narrowing inequalities in never and ex-smoking. Women generally had more favorable smoking outcomes than men. More research is needed to understand smoking initiation and cessation among immigrants and the factors influencing their smoking behaviors. Tobacco-control strategies should aim not only to improve overall health outcomes but also to address inequalities between subpopulations.

## Supplementary Material



## Data Availability

This study used data from the general release of the Household, Income and Labour Dynamics in Australia (HILDA) survey, which is available to approved researchers via the Australian Data Archive. L. Aung and A. Singh had full access to the dataset. The authors do not have permission to share the raw data directly.

## References

[1] World Health Organization. Tobacco: Key Facts; 2023. Accessed January 17, 2026. https://www.who.int/news-room/fact-sheets/detail/tobacco

[2] Wynne O, Bonevski B. Developments in the research base on reducing exposure to second-hand smoke. Int J Environ Res Public Health. 2018;15(9):1873. doi:10.3390/ijerph15091873 30200190 PMC6164103

[3] US Centers for Disease Control and Prevention. Cigarette Smoking; 2024. Accessed June 26, 2026. https://www.cdc.gov/tobacco/about/index.html

[4] Cnattingius S. The epidemiology of smoking during pregnancy: Smoking prevalence, maternal characteristics, and pregnancy outcomes. Nicotine Tob Res. 2004;6 suppl 2:s125-s140. doi:10.1080/14622200410001669187 15203816

[5] Banks E, Joshy G, Weber MF, et al. Tobacco smoking and all-cause mortality in a large Australian cohort study: Findings from a mature epidemic with current low smoking prevalence. BMC Medicine. 2015;13(1):38. doi:10.1186/s12916-015-0281-z 25857449 PMC4339244

[6] GBD 2019 Tobacco Collaborators. Spatial, temporal, and demographic patterns in prevalence of smoking tobacco use and attributable disease burden in 204 countries and territories, 1990-2019: A systematic analysis from the Global Burden of Disease Study 2019. Lancet. 2021;397(10292):2337-2360. doi:10.1016/S0140-6736(21)01169-7 34051883 PMC8223261

[7] World Health Organization. WHO global report on trends in prevalence of tobacco use 2000–2030; 2024. Accessed January 17, 2026. https://www.who.int/publications/i/item/9789240088283

[8] World Health Organization. WHO Global Report on Trends in Prevalence of Tobacco Use 2000-2025; 2019. Accessed January 17, 2026. https://www.who.int/publications/i/item/who-global-report-on-trends-in-prevalence-of-tobacco-use-2000-2025-third-edition

[9] Australian Bureau of Statistics. Insights into Australian Smokers, 2021-22; 2022. Accessed January 17, 2026. https://www.abs.gov.au/articles/insights-australian-smokers-2021-22

[10] Garrett BE, Dube SR, Babb S, McAfee T. Addressing the Social Determinants of Health to Reduce Tobacco-Related Disparities. Nicotine Tob Res. 2015;17(8):892-897. doi:10.1093/ntr/ntu266 25516538 PMC5104348

[11] Benowitz NL, Blum A, Braithwaite RL, Castro FG. Tobacco use among U.S. racial/ethnic minority groups - African Americans, American Indians and Alaska Natives, Asian Americans and Pacific Islanders, and Hispanics: a report of the Surgeon General. Executive summary. Centers for Disease Control and Prevention, National Center for Chronic Disease Prevention and Health Promotion, Office on Smoking and Health; 1998. Accessed June 26, 2026. https://digitalrepository.unm.edu/cgi/viewcontent.cgi?article=1025&context=nhd

[12] McEntee A, Kim S, Harrison N, Chapman J, Roche A. Patterns and prevalence of daily tobacco smoking in Australia by industry and occupation: 2007-2016. Nicotine Tob Res. 2021;23(12):2047-2055. doi:10.1093/ntr/ntab126 34129034

[13] Disney G, Petrie D, Yang Y, et al. Smoking inequality trends by disability and income in Australia, 2001 to 2020. Epidemiology. 2023;34(2):302. doi:10.1097/EDE.0000000000001582 36722813 PMC9891295

[14] Weber MF, Banks E, Sitas F. Smoking in migrants in New South Wales, Australia: report on data from over 100 000 participants in The 45 and Up Study. Drug Alcohol Rev. 2011;30(6):597-605. doi:10.1111/j.1465-3362.2010.00247.x 21355908

[15] Australian Bureau of Statistics. Cultural Diversity of Australia; 2022. Accessed January 17, 2026. https://www.abs.gov.au/articles/cultural-diversity-australia

[16] Pham TTL, Berecki-Gisolf J, Clapperton A, O’Brien KS, Liu S, Gibson K. Definitions of culturally and linguistically diverse (CALD): A literature review of epidemiological research in Australia. Int J Environ Res Public Health. 2021;18(2):737. doi:10.3390/ijerph18020737 33467144 PMC7830035

[17] Khatri RB, Assefa Y. Access to health services among culturally and linguistically diverse populations in the Australian universal health care system: Issues and challenges. BMC Public Health. 2022;22(1):880. doi:10.1186/s12889-022-13256-z 35505307 PMC9063872

[18] Joshi S, Jatrana S, Paradies Y. Tobacco smoking between immigrants and non-immigrants in Australia: A longitudinal investigation of the effect of nativity, duration of residence and age at arrival. Health Promot J Austr. 2018;29(3):282-292. doi:10.1002/hpja.19 30511489

[19] Rowe R, Gavriel Ansara Y, Jaworski A, Higgs P, Clare PJ. What is the alcohol, tobacco, and other drug prevalence among culturally and linguistically diverse groups in the Australian population? A national study of prevalence, harms, and attitudes. J Ethn Subst Abuse. 2020;19(1):101-118. doi:10.1080/15332640.2018.1484310 30064336

[20] Amiri S. Worldwide prevalence of smoking in immigration: A global systematic review and meta-analysis. J Addict Dis. 2020;38(4):567-579. doi:10.1080/10550887.2020.1800888 32780650

[21] Australian Government Department of Social Services. Living in Australia: The Household, Income and Labour Dynamics in Australia (HILDA) Survey. Accessed January 17, 2026. https://www.dss.gov.au/long-term-research/living-australia-household-income-and-labour-dynamics-australia-hilda-survey

[22] Australian Bureau of Statistics. Standard Australian Classification of Countries (SACC); 2016. Accessed January 17, 2026. https://www.abs.gov.au/statistics/classifications/standard-australian-classification-countries-sacc/latest-release

[23] Maheen H, King T. Employment-related mental health outcomes among Australian migrants: A 19-year longitudinal study. Aust N Z J Psychiatry. 2023;57(11):1475-1485. doi:10.1177/00048674231174809 37211808 PMC10619185

[24] Mansournia MA, Altman DG. Inverse probability weighting. BMJ. 2016;352:i189. doi:10.1136/bmj.i189 26773001

[25] R Core Team. R: A Language and Environment for Statistical Computing. Accessed January 17, 2026. https://www.R-project.org/

[26] West R. Tobacco smoking: Health impact, prevalence, correlates and interventions. Psychol Health. 2017;32(8):1018-1036. doi:10.1080/08870446.2017.1325890 28553727 PMC5490618

[27] Koslowski R. Selective migration policy models and changing realities of implementation. International Migration. 2014;52(3):26–39. doi:10.1111/imig.1213625346548

[28] Reiss K, Lehnhardt J, Razum O. Factors associated with smoking in immigrants from non-western to western countries - what role does acculturation play? A systematic review. Tob Induc Dis. 2015;13(1):11. doi:10.1186/s12971-015-0036-9 25908932 PMC4407357

[29] Action on Smoking and Health. Health inequalities and smoking; 2019. Accessed January 17, 2026. https://ash.org.uk/resources/view/health-inequalities-and-smoking

[30] Freedman KS, Nelson NM, Feldman LL. Smoking initiation among young adults in the United States and Canada, 1998-2010: A systematic review. Prev Chronic Dis. 2012;9:E05. doi:10.5888/pcd9.110037 22172172 PMC3277388

[31] Stockings EA, Gardner LA, Newton NC. Vaping among young people-Our best defence is self-defence. Drug Alcohol Rev. 2024;43(2):355-358. doi:10.1111/dar.13746 37782572

[32] Hair EC, Barton AA, Perks SN, et al. Association between e-cigarette use and future combustible cigarette use: Evidence from a prospective cohort of youth and young adults, 2017-2019. Addict Behav. 2021;112:106593. doi:10.1016/j.addbeh.2020.106593 32927247

[33] Van Brussel L. Putting proportionate universalism into practice: Challenges and tools. Int J Integr Care. 2023;23(S1):370. doi:10.5334/ijic.ICIC23480

