# Supplementary material for: Smoking inequalities among culturally diverse populations in Australia: A secondary dataset analysis of the HILDA survey 2002–2019
Source: Tob Induc Dis. 2026 Jun 30;24:10.18332/tid/217085. doi: 10.18332/tid/217085 (PMC13320934; doi:10.18332/tid/217085)
Supplement: Supplementary file 1 [file TID-24-100-s1.pdf]

Supplementary Figure 1 − Prevalence and Trends of Never Smoking of Subpopulations of Australia by Sex (2002−2019)

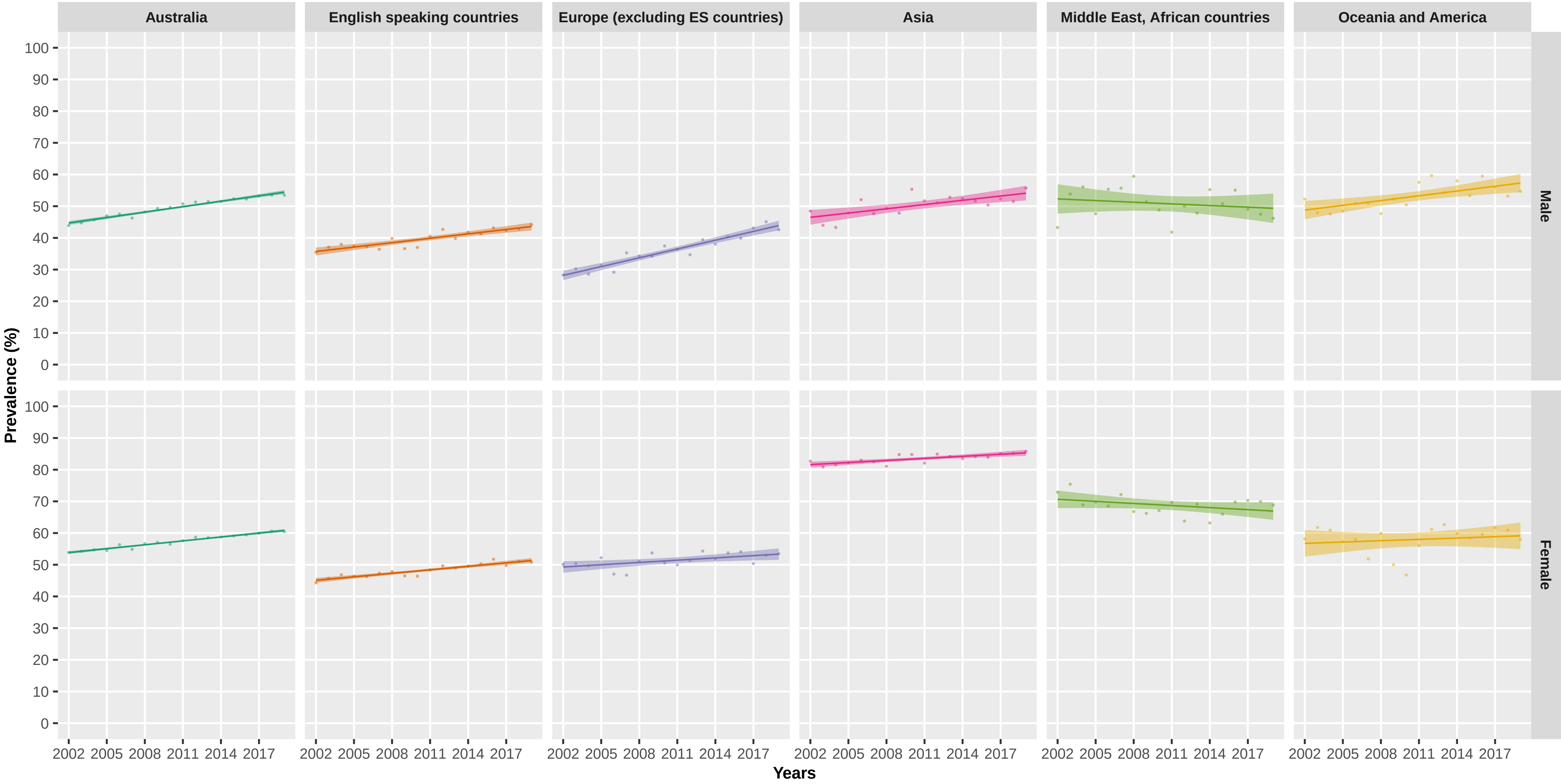

Data source: Secondary Dataset Analysis of HILDA Survey Waves 2 to 19

Supplementary Figure 2 − Inequality in Never Smoking of Subpopulations of Australia by Sex (2002−2019)

Reference group: Australia

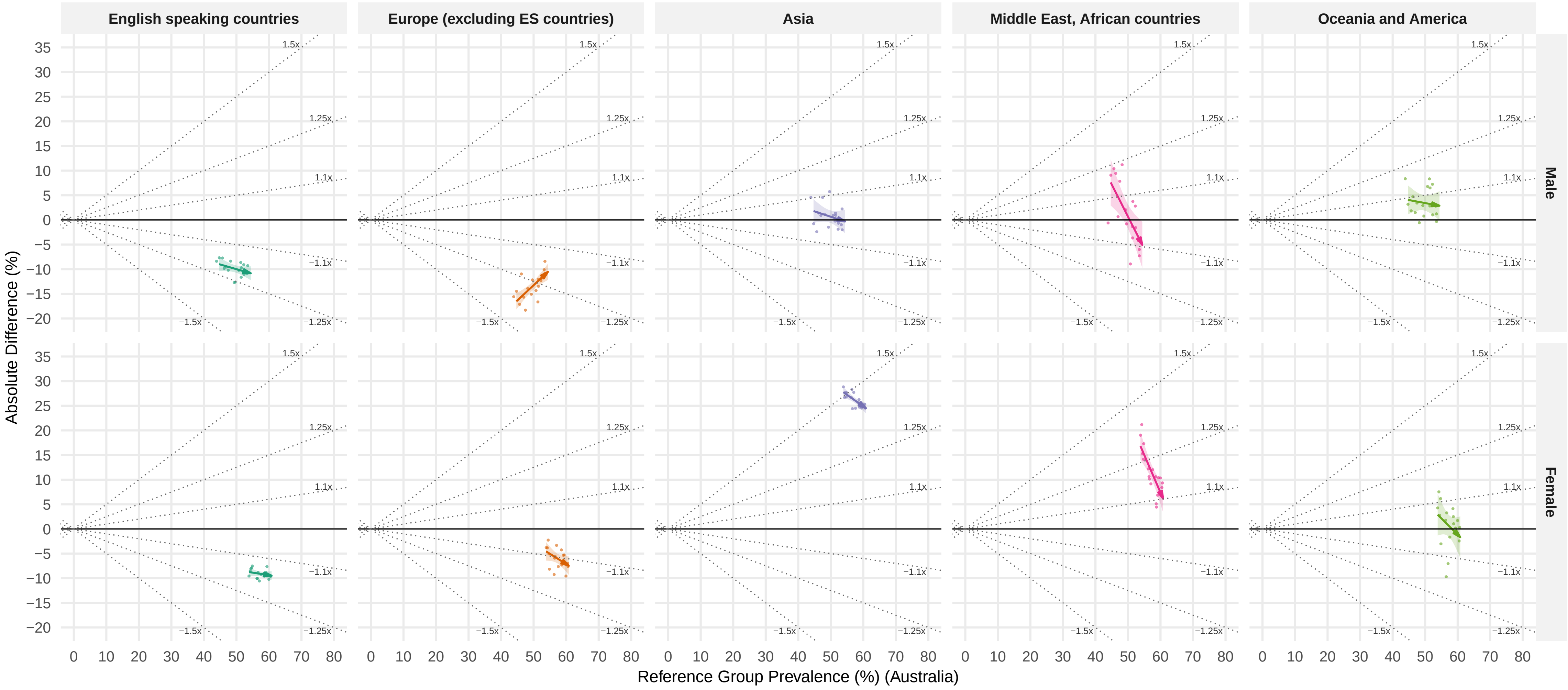

Dotted lines represent relative inequality ratios. The arrows summarize smoking change and inequality relative to the Australian−born reference group. The x−axis shows change in prevalence over time: arrows pointing right indicate an increase, and arrows pointing left indicate a decrease. The y−axis shows the difference from the reference group: values above 0 indicate higher prevalence than the reference group, and values below 0 indicate lower prevalence. Arrows closer to y = 0 indicate smaller inequalities, while arrows farther from y = 0 indicate larger inequalities. Data source: Secondary Dataset Analysis of HILDA Survey Waves 2 to 19

Supplementary Figure 3 − Prevalence and Trends of Current Smoking of Subpopulations of Australia by Sex (2002−2019)

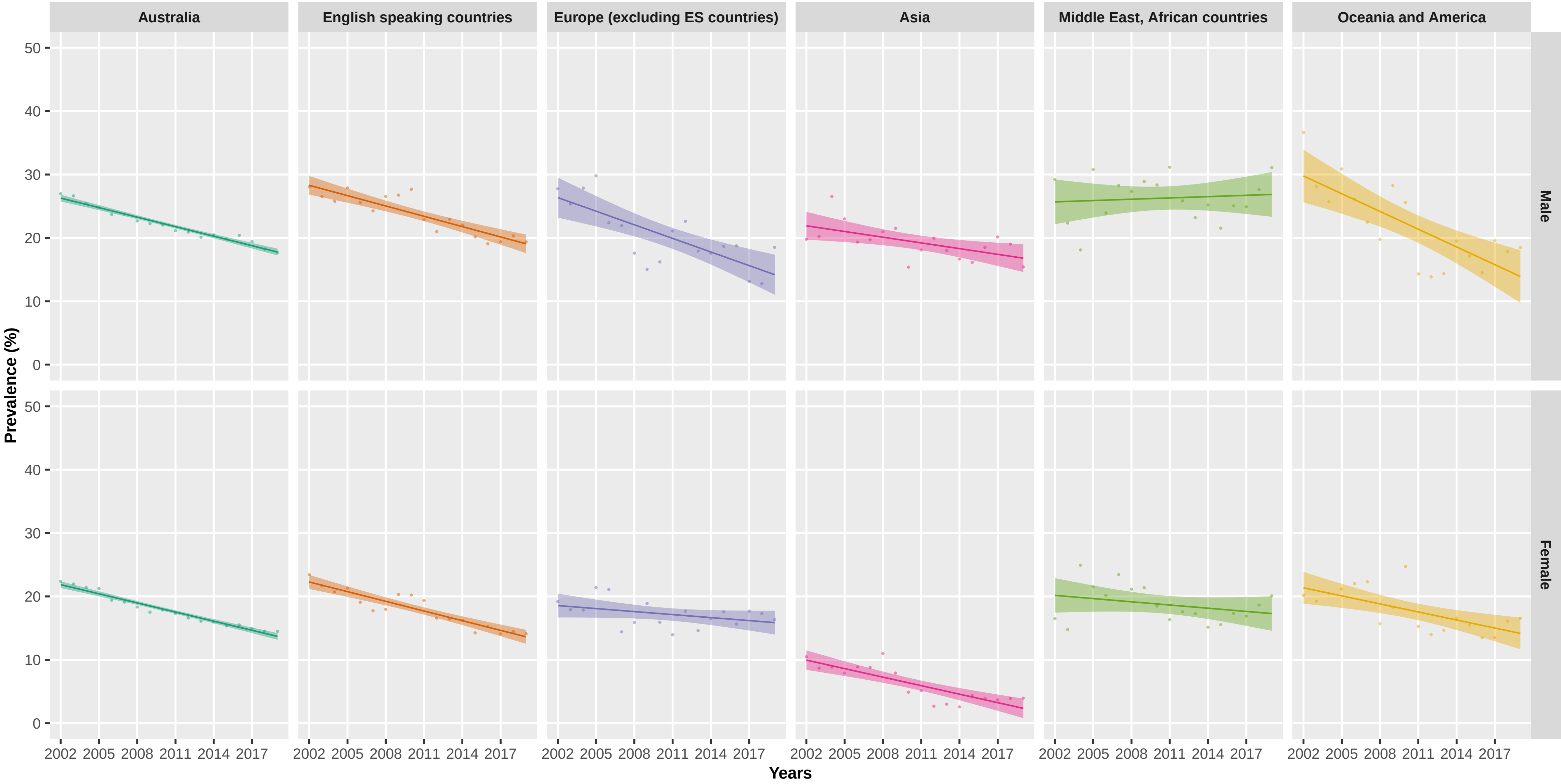

Data source: Secondary Dataset Analysis of HILDA Survey Waves 2 to 19

Supplementary Figure 4 − Inequality in Current Smoking of Subpopulations of Australia by Sex (2002−2019)

Reference group: Australia

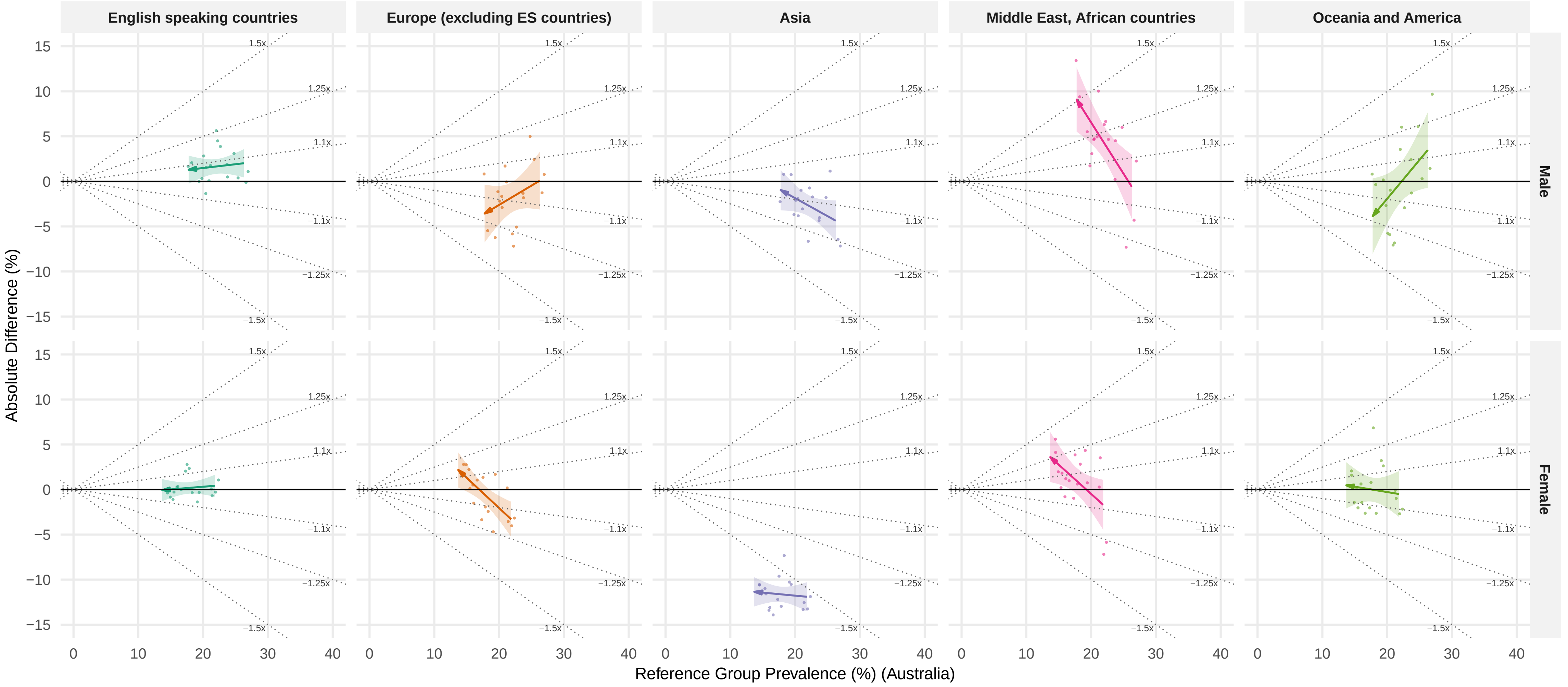

Dotted lines represent relative inequality ratios. The arrows summarize smoking change and inequality relative to the Australian−born reference group. The x−axis shows change in prevalence over time: arrows pointing right indicate an increase, and arrows pointing left indicate a decrease. The y−axis shows the difference from the reference group: values above 0 indicate higher prevalence than the reference group, and values below 0 indicate lower prevalence. Arrows closer to y = 0 indicate smaller inequalities, while arrows farther from y = 0 indicate larger inequalities. Data source: Secondary Dataset Analysis of HILDA Survey Waves 2 to 19

Supplementary Figure 5 − Prevalence and Trends of Ex Smoking by Sex and Subpopulation (2002−2019)

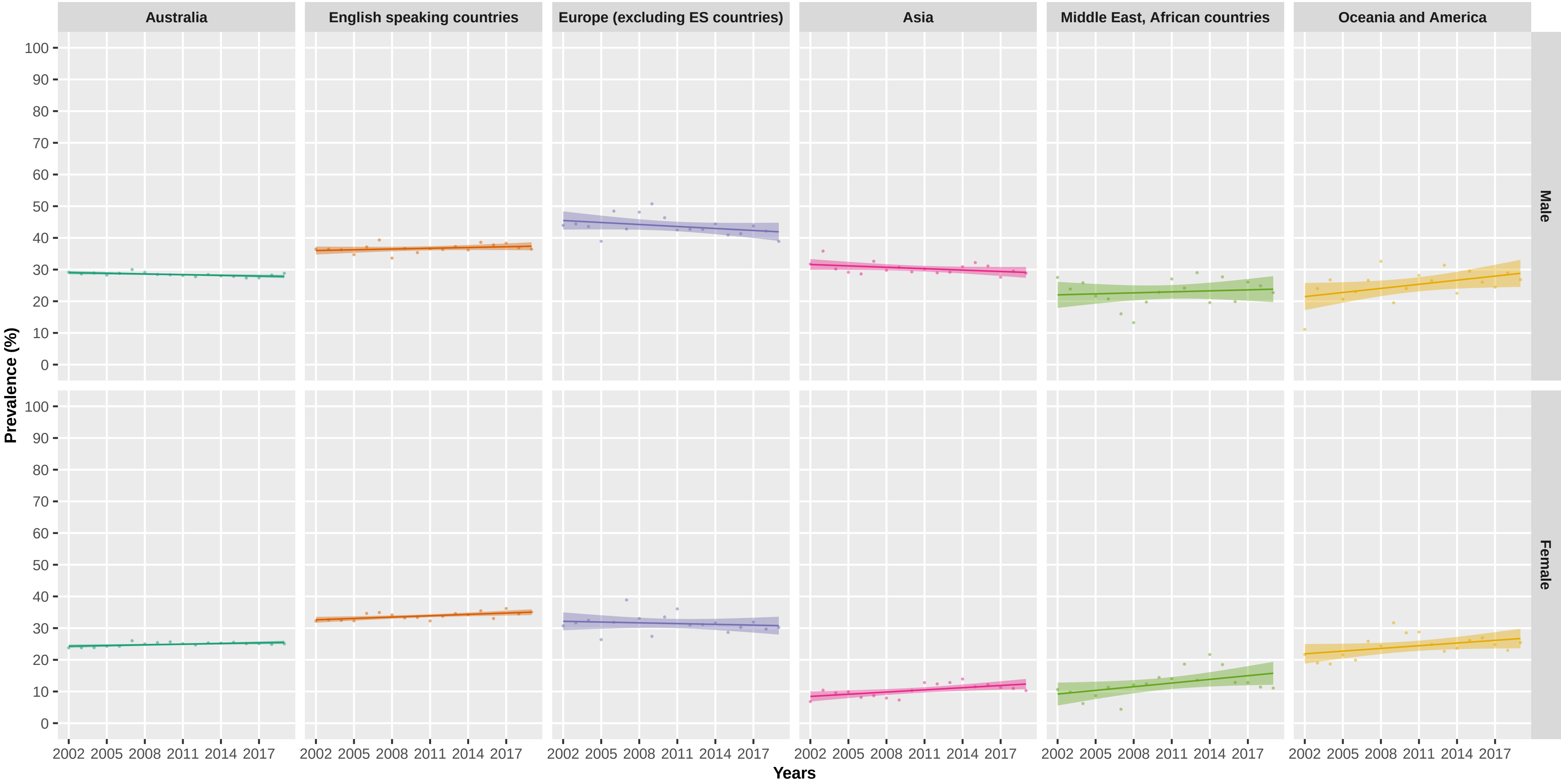

Data source: Secondary Dataset Analysis of HILDA Survey Waves 2 to 19

Supplementary Figure 6 − Inequality in Ex Smoking by Sex and Subpopulation (2002−2019)*

Reference group: Australia

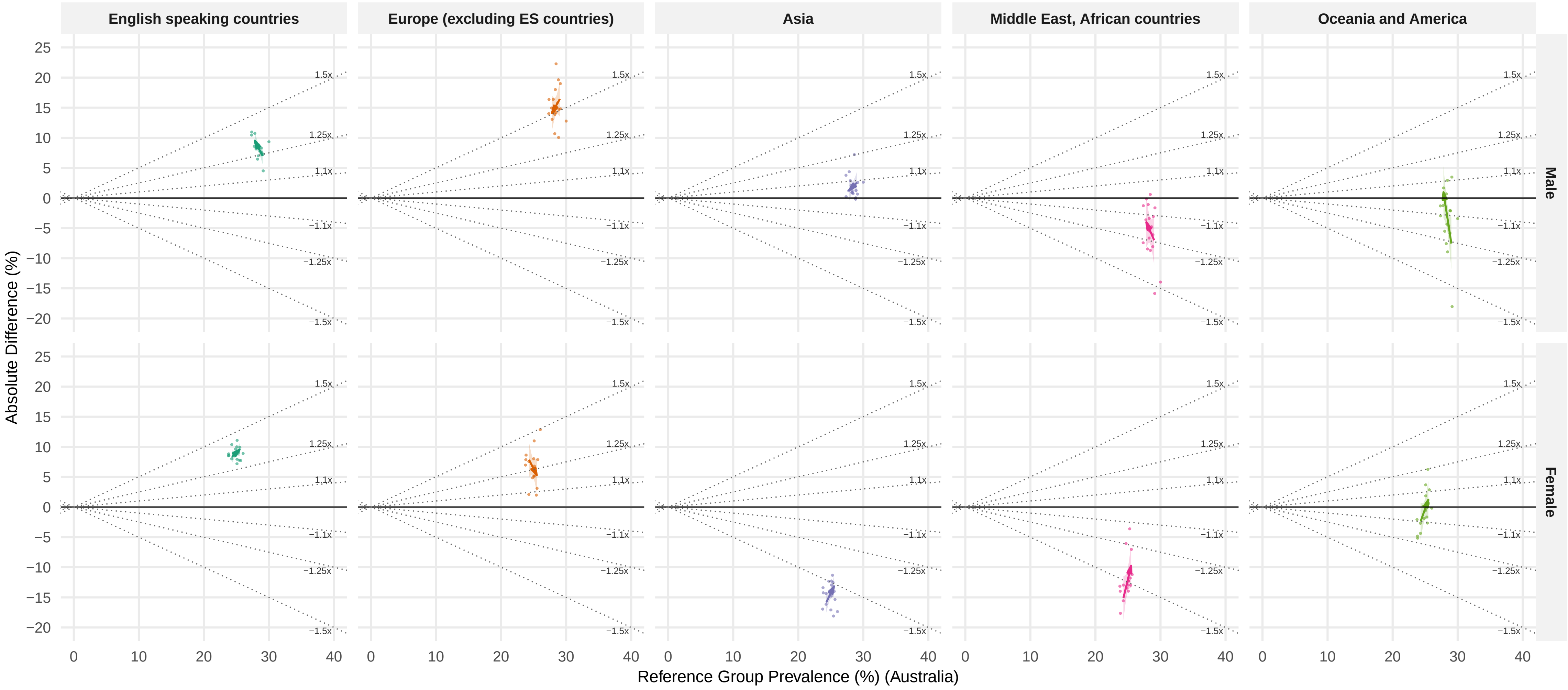

Dotted lines represent relative inequality ratios. The arrows summarize smoking change and inequality relative to the Australian−born reference group. The x−axis shows change in prevalence over time: arrows pointing right indicate an increase, and arrows pointing left indicate a decrease. The y−axis shows the difference from the reference group: values above 0 indicate higher prevalence than the reference group, and values below 0 indicate lower prevalence. Arrows closer to y = 0 indicate smaller inequalities, while arrows farther from y = 0 indicate larger inequalities. Data source: Secondary Dataset Analysis of HILDA Survey Waves 2 to 19

Figure 7. Covariate Balance Comparison Of Adjusted and Unadjusted Across HILDA Waves (Love Plot)

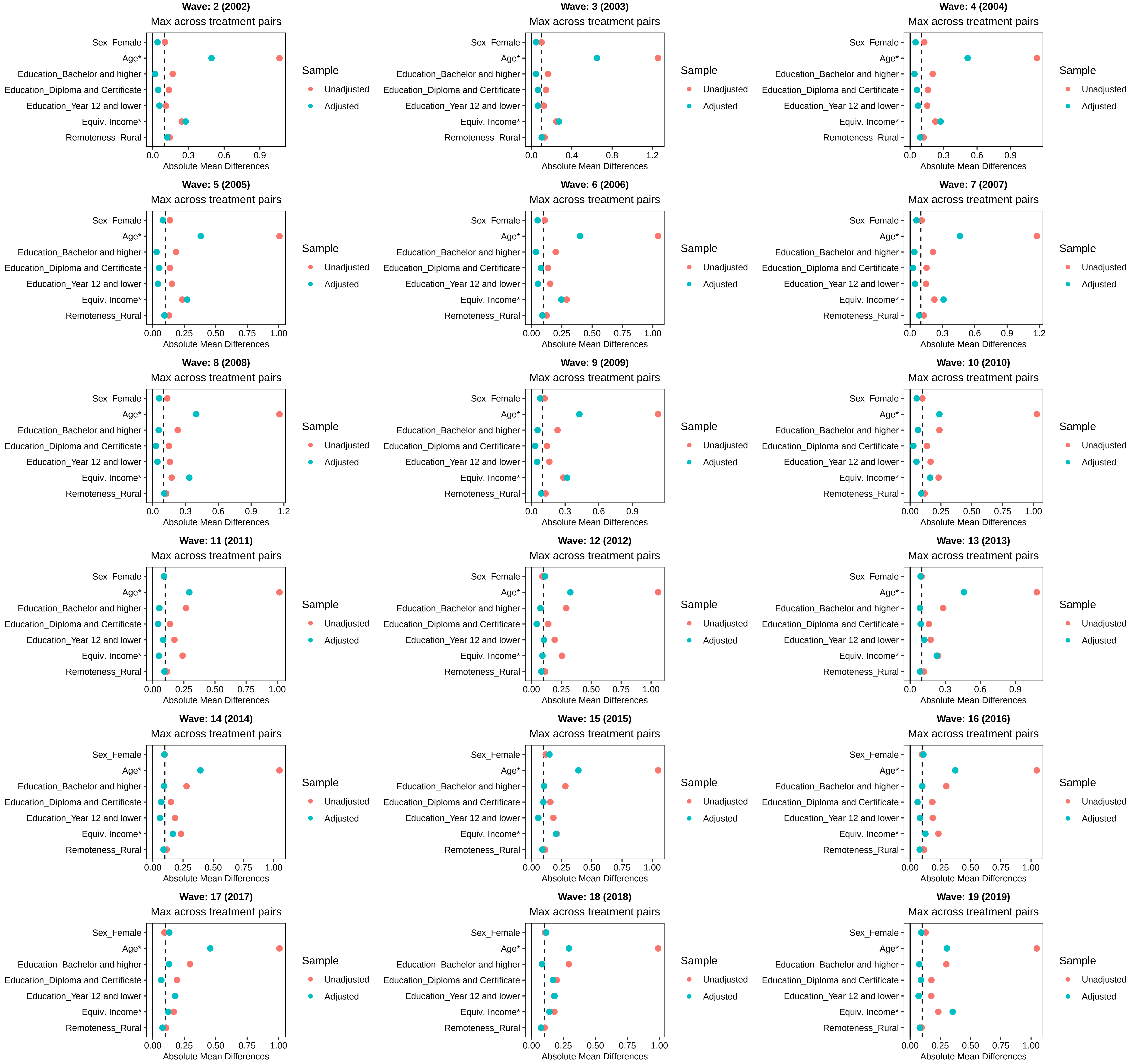

Note: Love plot comparing covariate balance before and after inverse probability weighting (IPW); smaller absolute mean differences (max pairwise) indicate better balance.
The dashed vertical line at 0.1 indicates the commonly used threshold for acceptable balance.
Data source: Household, Income and Labour Dynamics in Australia (HILDA) Survey, 2002−2019.

Subpopulation	Sex	Prevalence(P)	P_LCI	P_UCI	Year	Linear(lr)	lr_LCI	lr_UCI	p_value	Difference(D)	D_LCI	D_UCI	Ratio(R)	R_LCI	R_UCI	Australia	Australia
Australia	Male	43,8876541	42,4111204	45,3750902	2002	44,6925776	44,0646419	45,3205133	0	0	-0,88803516	0,88803516	1	0,98032625	1,02006858	0,98032625	1,02006858
Australia	Female	53,8757253	52,4725425	55,2727933	2002	53,8420974	53,3890949	54,2950998	0	0	-0,64064225	0,64064225	1	0,98817197	1,01196961	0,98817197	1,01196961
English speakin	Male	35,4934938	31,5708668	39,6213018	2002	35,7043424	34,4456404	36,9630444	0	-8,98823515	-10,3948744	-7,58159592	0,79888752	0,7691377	0,82978804	0,7691377	0,82978804
English speakin	Female	44,3157135	40,2225835	48,4875107	2002	45,0897711	44,3094176	45,8701246	0	-8,75232627	-9,65463598	-7,85001657	0,83744455	0,82148334	0,85371588	0,82148334	0,85371588
Europe (exclud	Male	28,2974934	22,6392744	34,7349126	2002	28,1619846	26,6430733	29,6808958	0	-16,530593	-18,174185	-14,887001	0,63012666	0,59596744	0,66624379	0,59596744	0,66624379
Europe (exclud	Female	50,078022	43,888306	56,2653476	2002	49,2852557	47,4726553	51,097856	0,013	-4,55684169	-6,42519158	-2,68849181	0,91536656	0,88147511	0,9505611	0,88147511	0,9505611
Asia	Male	48,472888	40,6522191	56,3690709	2002	46,4902649	44,1643064	48,8162234	0,001	1,79768735	-0,61154203	4,20691672	1,0402234	0,9875472	1,09570937	0,9875472	1,09570937
Asia	Female	82,7065864	75,6248131	88,0558357	2002	81,6027228	80,6522888	82,5531567	0	27,7606254	26,7077551	28,8134957	1,51559331	1,49397282	1,5375267	1,49397282	1,5375267
Middle East, Af	Male	43,2654194	32,3988511	54,8210152	2002	52,2985375	47,6506051	56,9464699	0,441	7,60595996	2,91580228	12,2961176	1,17018396	1,06949239	1,28035554	1,06949239	1,28035554
Middle East, Af	Female	72,8891873	60,9319079	82,2527234	2002	70,6457779	67,8734541	73,4181017	0,117	16,8036805	13,9945898	19,6127713	1,31209186	1,2604746	1,36582289	1,2604746	1,36582289
Oceania and Ar	Male	52,2401286	39,3608611	64,8282652	2002	48,762526	45,8753741	51,649678	0,002	4,06994846	1,11529966	7,02459726	1,09106542	1,026651	1,15952136	1,026651	1,15952136
Oceania and Ar	Female	58,1430016	45,3984864	69,8860146	2002	56,7435557	52,5282268	60,9588845	0,493	2,9014583	-1,33814176	7,14105835	1,05388829	0,97797044	1,13569947	0,97797044	1,13569947
Australia	Male	44,7450245	43,244463	46,2552191	2003	45,2647539	44,6897022	45,8398057	0	0	-0,81324603	0,81324603	1	0,982194	1,0181288	0,982194	1,0181288
Australia	Female	54,2385485	52,8260192	55,644296	2003	54,252073	53,8372218	54,6669242	0	0	-0,58668822	0,58668822	1	0,98924415	1,0108728	0,98924415	1,0108728
English speakin	Male	37,0717308	32,9613985	41,378257	2003	36,167757	35,0150612	37,3204528	0	-9,09699692	-10,3851709	-7,80882297	0,79902692	0,77207762	0,82691688	0,77207762	0,82691688
English speakin	Female	45,6950378	41,3768226	50,0789363	2003	45,4571633	44,7425301	46,1717965	0	-8,79490967	-9,62122806	-7,96859128	0,83788804	0,82336731	0,85266486	0,82336731	0,85266486
Europe (exclud	Male	30,2454132	23,9276599	37,4109324	2003	29,087064	27,6960734	30,4780546	0	-16,1776899	-17,6828608	-14,6725191	0,64259852	0,61157635	0,67519429	0,61157635	0,67519429
Europe (exclud	Female	50,4078724	43,6901257	57,110925	2003	49,5244295	47,8644839	51,1843751	0,013	-4,72764346	-6,43864342	-3,0166435	0,91285783	0,8820081	0,94478658	0,8820081	0,94478658
Asia	Male	43,966418	36,015065	52,2400044	2003	46,9377457	44,8076763	49,0678151	0,001	1,67299176	-0,53333555	3,87931907	1,03696014	0,9892265	1,0869971	0,9892265	1,0869971
Asia	Female	80,9000487	73,3226984	86,7151018	2003	81,8209226	80,9505329	82,6913122	0	27,5688496	26,6046506	28,5330486	1,50816214	1,4885327	1,52805044	1,4885327	1,52805044
Middle East, Af	Male	53,8245829	41,1140385	66,0566232	2003	52,124554	47,8680642	56,3810438	0,441	6,85980006	2,56464119	11,1549589	1,15154838	1,06020805	1,25075797	1,06020805	1,25075797
Middle East, Af	Female	75,4078078	62,9950434	84,6701273	2003	70,428392	67,8895496	72,9672345	0,117	16,1763191	13,6038062	18,7488319	1,2981696	1,2512019	1,34690038	1,2512019	1,34690038
Oceania and Ar	Male	47,9206671	35,1255245	60,9945603	2003	49,2645005	46,6205007	51,9085004	0,002	3,9997466	1,29393436	6,70555883	1,08836338	1,02996259	1,1500756	1,02996259	1,1500756
Oceania and Ar	Female	61,7574098	48,3813214	73,5614524	2003	56,8836697	53,0233502	60,7439892	0,493	2,63159672	-1,2509499	6,51414334	1,04850684	0,97929166	1,12261407	0,97929166	1,12261407
Australia	Male	45,7003434	44,1719586	47,2368663	2004	45,8369303	45,3125085	46,3613521	0	0	-0,74164441	0,74164441	1	0,98395013	1,01631167	0,98395013	1,01631167
Australia	Female	54,7529531	53,3261602	56,1719774	2004	54,6620486	54,2837226	55,0403745	0	0	-0,5350337	0,5350337	1	0,99025972	1,00983609	0,99025972	1,00983609
English speakin	Male	37,9705992	33,7706237	42,3588381	2004	36,6311716	35,579964	37,6823792	0	-9,2057587	-10,3805164	-8,03100102	0,79916284	0,77485123	0,82423725	0,77485123	0,82423725
English speakin	Female	46,8490172	42,5315014	51,2142314	2004	45,8245555	45,1728417	46,4762693	0	-8,83749307	-9,59105889	-8,08392724	0,83832488	0,82516971	0,85168978	0,82516971	0,85168978
Europe (exclud	Male	28,5603193	22,183659	35,9237583	2004	30,0121434	28,7436216	31,2806653	0	-15,8247869	-17,1974361	-14,4521376	0,65475902	0,62670705	0,68406661	0,62670705	0,68406661
Europe (exclud	Female	49,6758163	42,6670664	56,6973297	2004	49,7636034	48,2498064	51,2774003	0,013	-4,89844523	-6,45880151	-3,33808894	0,91038672	0,88242367	0,9392359	0,88242367	0,9392359
Asia	Male	43,2950957	35,164571	51,8034785	2004	47,3852265	45,4426974	49,3277556	0,001	1,54829617	-0,46377675	3,56036909	1,03377836	0,99070289	1,07872673	0,99070289	1,07872673
Asia	Female	81,5460412	73,9208207	87,3240617	2004	82,0391224	81,2453655	82,8328793	0	27,3770738	26,497767	28,2563806	1,50084244	1,48309418	1,5188031	1,48309418	1,5188031
Middle East, Af	Male	56,0757103	43,5222056	67,8970973	2004	51,9505705	48,0688401	55,8323009	0,441	6,11364017	2,19664525	10,0306351	1,13337805	1,05086334	1,22237187	1,05086334	1,22237187
Middle East, Af	Female	68,8893772	55,5766648	79,6718031	2004	70,2110062	67,8956942	72,5263182	0,117	15,5489576	13,2029396	17,8949755	1,28445618	1,2418975	1,32847331	1,2418975	1,32847331
Oceania and Ar	Male	47,5486295	33,9066988	61,5664856	2004	49,7664751	47,3552641	52,177686	0,002	3,92954474	1,46196365	6,39712582	1,08572879	1,03300129	1,14114766	1,03300129	1,14114766
Oceania and Ar	Female	60,913381	47,1922643	73,1013961	2004	57,0237837	53,5033431	60,5442244	0,493	2,36173514	-1,17897565	5,90244593	1,04320612	0,98037088	1,11006868	0,98037088	1,11006868
Australia	Male	46,967862	45,4452421	48,4961474	2005	46,4091067	45,9323424	46,885871	0	0	-0,67424656	0,67424656	1	0,9855767	1,01463437	0,9855767	1,01463437
Australia	Female	54,4797799	53,0604126	55,8919059	2005	55,0720242	54,7280791	55,4159693	0	0	-0,48641186	0,48641186	1	0,9912066	1,00887141	0,9912066	1,00887141
English speakin	Male	37,4113955	33,1894845	41,8340786	2005	37,0945862	36,1389084	38,050264	0	-9,31452047	-10,3825206	-8,24652035	0,79929542	0,77743083	0,82177493	0,77743083	0,82177493
English speakin	Female	46,407353	42,0635993	50,8063102	2005	46,1919477	45,5994592	46,7844363	0	-8,88007646	-9,56516106	-8,19499186	0,83875522	0,82687422	0,85080693	0,82687422	0,85080693
Europe (exclud	Male	31,2896873	24,6703452	38,7709733	2005	30,9372229	29,7839795	32,0904662	0	-15,4718838	-16,7197919	-14,2239758	0,66661966	0,64133574	0,69290036	0,64133574	0,69290036
Europe (exclud	Female	52,2141221	45,201885	59,1401635	2005	50,0027772	48,6265484	51,379006	0,013	-5,06924699	-6,48780398	-3,65069001	0,90795241	0,88268574	0,93394234	0,88268574	0,93394234
Asia	Male	47,8751052	39,3695164	56,5057079	2005	47,8327073	46,0667079	49,5987066	0,001	1,42360058	-0,40562273	3,25282389	1,03067503	0,99192381	1,07094012	0,99192381	1,07094012
Asia	Female	82,1913304	75,102317	87,5953031	2005	82,2573222	81,5356989	82,9789454	0	27,185298	26,3858992	27,9846967	1,49363172	1,47763348	1,50980317	1,47763348	1,50980317
Middle East, Af	Male	47,6176901	35,3854744	60,1425592	2005	51,776587	48,2476136	55,3055603	0,441	5,36748028	1,8064471	8,92851346	1,11565576	1,04134671	1,19526739	1,04134671	1,19526739
Middle East, Af	Female	69,7721472	56,3975346	80,46525	2005	69,9936203	67,8887151	72,0985255	0,117	14,9215961	12,7887754	17,0544168	1,27094693	1,23250387	1,31058908	1,23250387	1,31058908
Oceania and Ar	Male	48,4583454	34,5963177	62,5617191	2005	50,2684496	48,0763604	52,4605387	0,002	3,85934287	1,61600627	6,10267948	1,08315917	1,03570313	1,13278965	1,03570313	1,13278965
Oceania and Ar	Female	57,2478751	44,1050882	69,4414928	2005	57,1638978	53,9633815	60,364414	0,493	2,09187356	-1,12707077	5,3108179	1,03798432	0,98112541	1,09813837	0,98112541	1,09813837
Australia	Male	47,4907396	45,9871533	48,9988896	2006	46,9812831	46,5482212	47,4143449	0	0	-0,6124419	0,6124419	1	0,98704873	1,01312121	0,98704873	1,01312121
Australia	Female	56,3147586	54,9147371	57,7047896	2006	55,4819998	55,1695823	55,7944173	0	0	-0,44182502	0,44182502	1	0,99206823	1,00799519	0,99206823	1,00799519
English speakin	Male	37,2684033	33,0374999	41,7036875	2006	37,5580008	36,689925	38,4260766	0	-9,42328224	-10,3933844	-8,45318011	0,79942476	0,77977794	0,81956658	0,77977794	0,81956658
English speakin	Female	46,2644267	41,9234173	50,6628164	2006	46,5593399	46,0211617	47,0975181	0	-8,92265986	-9,54494634	-8,30037338	0,8391792	0,82845844	0,85003868	0,82845844	0,85003868
Europe (exclud	Male	29,1723809	22,6632621	36,6646781	2006	31,8623023	30,8147707	32,9098339	0	-15,1189808	-16,2524996	-13,9854619	0,6781914	0,65542571	0,70174785	0,65542571	0,70174785
Europe (exclud	Female	47,0604378	39,9969944	54,243613	2006	50,241951	48,991874	51,4920281	0,013	-5,24004876	-6,52857404	-3,95152348	0,90555408	0,88274522	0,92895228	0,88274522	0,92895228
Asia	Male	52,0514454	43,3408488	60,639071	2006	48,2801881	46,6760686	49,8843075	0,001	1,29890499	-0,36264298	2,96045297	1,02764729	0,99281776	1,06369869	0,99281776	1,06369869
Asia	Female	82,9910151	75,3147244	88,640279	2006	82,475522	81,8200462	83,1309978	0	26,9935222	26,2674001	27,7196442	1,48652756	1,47211882	1,50107734	1,47211882	1,50107734
Middle East, Af	Male	55,3376473	42,5713128	67,436808	2006	51,6026035	48,3971126	54,8080943	0,441	4,62132039	1,3867085	7,85593227	1,09836514	1,03151013	1,16955321	1,03151013	1,16955321
Middle East, Af	Female	68,5311998	55,89149	78,9152699	2006	69,7762344	67,8642749	71,688194	0,117	14,2942346	12,3569185	16,2315508	1,25763734	1,22294383	1,29331505	1,22294383	1,29331505
Oceania and Ar	Male	50,9121416	36,688434	64,9896866	2006	50,7704241	48,7792723	52,7615759	0,002	3,78914101	1,75143934	5,82684268	1,08065214	1,03798078	1,12507771	1,03798078	1,12507771
Oceania and Ar	Female	58,0680019	44,5392905	70,4836042	2006	57,3040118	54,3968701	60,2111535	0,493	1,82201198	-1,10186855	4,74589252	1,0328397	0,98144289	1,08692808	0,98144289	1,08692808
Australia	Male	46,2398898	44,7175655	47,7692573	2007	47,5534594	47,158829	47,9480899	0	0	-0,55809176	0,55809176	1	0,98833251	1,01180523	0,98833251	1,01180523
Australia	Female	54,8695817	53,4531508	56,2781657	2007	55,8919754	55,6072829	56,1766679	0	0	-0,40261599	0,40261599	1	0,99282242	1,00722947	0,99282242	1,00722947
English speakin	Male	36,406957	31,9287627	41,1337038	2007	38,0214154	37,2303756	38,8124553	0	-9,53204402	-10,416056	-8,64803199	0,79955099	0,78184083	0,81766231	0,78184083	0,81766231
English speakin	Female	47,3482956	42,8215603	51,9190469	2007	46,9267322	46,4363137	47,4171506	0	-8,96524326	-9,53230595	-8,39818056	0,83959695	0,82989236	0,84941502	0,82989236	0,84941502
Europe (exclud	Male	35,271659	27,7248683	43,6325509	2007	32,7873817	31,8328116	33,7419518	0	-14,7660777	-15,7990043	-13,7331511	0,68948468	0,66892429	0,71067702	0,66892429	0,71067702
Europe (exclud	Female	46,6999562	39,3608469	54,1847211	2007	50,4811249	49,3419838	51,620266	0,013	-5,41085053	-6,58502777	-4,23667329	0,90319092	0,88253685	0,92432836	0,88253685	0,92432836
Asia	Male	47,6418891	39,3513997	56,0643995	2007	48,7276688	47,2659043	50,1894334	0,001	1,17420941	-0,33988724	2,68830606	1,02469241	0,99328975	1,05708786	0,99328975	1,05708786
Asia	Female	82,5023505	75,2593664	87,9640168	2007	82,6937218	82,0964151	83,2910285	0	26,8017464	26,1400628	27,4634299	1,47952763	1,4665085	1,49266234	1,4665085	1,49266234
Middle East, Af	Male	55,684379	42,9080527	67,7504806	2007	51,4286199	48,5075951	54,3496448	0,441	3,8751605	0,92759889	6,8227221	1,08149061	1,02116035	1,14538519	1,02116035	1,14538519
Middle East, Af	Female	72,1642248	59,677502	81,9534841	2007	69,5588486	67,8165627	71,3011344	0,117	13,6668732	11,9014809	15,4322654	1,24452299	1,21311569	1,27674343	1,21311569	1,27674343
Oceania and Ar	Male	50,9417649	37,1629501	64,5789601	2007	51,2723986	49,4579483	53,0868489	0,002	3,71893915	1,86206994	5,57580836	1,07820544	1,03971806	1,11811751	1,03971806	1,11811751
Oceania and Ar	Female	51,8341725	38,9071709	64,5202175	2007	57,4441258	54,7949736	60,093278	0,493	1,5521504	-1,11225519	4,216556	1,02777054	0,98117393	1,07658005	0,98117393	1,07658005
Australia	Male	48,2097984	46,6728429	49,7501489	2008	48,1256358	47,7624882	48,4887835	0	0	-0,51356831	0,51356831	1	0,98938533	1,01072855	0,98938533	1,01072855
Australia	Female	56,6568104	55,2285184	58,0741274	2008	56,301951	56,0399707	56,5639313	0	0	-0,37049609	0,37049609	1	0,99344108	1,00660222	0,99344108	1,00660222
English speakin	Male	39,8286733	35,1711966	44,6778586	2008	38,48483	37,7568978	39,2127623	0	-9,64080579	-10,4542931	-8,82731848	0,79967421	0,78355403	0,81612604	0,78355403	0,81612604
English speakin	Female	47,8378634	43,1197702	52,5948508	2008	47,2941244	46,8428305	47,7454182	0	-9,00782665	-9,52965021	-8,48600309	0,84000862	0,83113797	0,84897395	0,83113797	0,84897395
Europe (exclud	Male	34,2909172	25,9348145	43,7490272	2008	33,7124611	32,8340447	34,5908776	0	-14,4131747	-15,3636965	-13,4626529	0,70050942	0,68176228	0,71977206	0,68176228	0,71977206
Europe (exclud	Female	51,0679923	42,8303749	59,2480111	2008	50,7202987	49,6720361	51,7685614	0,013	-5,58165229	-6,66215601	-4,50114858	0,90086219	0,88197829	0,92015041	0,88197829	0,92015041
Asia	Male	49,2860911	40,6581165	57,9568008	2008	49,1751496	47,8300018	50,5202975	0,001	1,04951382	-0,34379121	2,44281885	1,02180779	0,99322057	1,05121782	0,99322057	1,05121782
Asia	Female	81,0751035	73,0985263	87,1038027	2008	82,9119216	82,3622668	83,4615764	0	26,6099706	26,0010748	27,2188663	1,47262964	1,46075039	1,48460549	1,46075039	1,48460549
Middle East, Af	Male	59,4049185	45,9172286	71,6087343	2008	51,2546364	48,5666451	53,9426277	0,441	3,1290006	0,4165896	5,84141161	1,06501734	1,01005737	1,12296782	1,01005737	1,12296782
Middle East, Af	Female	66,7903877	53,1462457	78,0986296	2008	69,3414627	67,738173	70,9447524	0,117	13,0395117	11,4149589	14,6640644	1,23159964	1,20289196	1,26099245	1,20289196	1,26099245
Oceania and Ar	Male	47,6494715	33,9836079	61,676597	2008	51,7743731	50,104676	53,4440702	0,002	3,64873729	1,94000541	5,35746917	1,07581692	1,04076891	1,11204517	1,04076891	1,11204517
Oceania and Ar	Female	59,9148951	45,8463423	72,519339	2008	57,5842399	55,1464317	60,022048	0,493	1,28228883	-1,1695558	3,73413345	1,02277521	0,98013019	1,06727569	0,98013019	1,06727569
Australia	Male	49,3049918	47,7879636	50,8233009	2009	48,6978122	48,3572663	49,0383581	0	0	-0,48160468	0,48160468	1	0,99015908	1,00993872	0,99015908	1,00993872
Australia	Female	57,0585882	55,6489206	58,4568985	2009	56,7119266	56,4662515	56,9576017	0	0	-0,34743704	0,34743704	1	0,99389238	1,00614515	0,99389238	1,00614515
English speakin	Male	36,5928534	32,1738312	41,2496963	2009	38,9482446	38,2656176	39,6308716	0	-9,74956756	-10,5124248	-8,98671033	0,79979455	0,78484383	0,81503006	0,78484383	0,81503006
English speakin	Female	46,4894429	41,8420842	51,1985689	2009	47,6615166	47,2383105	48,0847227	0	-9,05041005	-9,53975619	-8,56106391	0,84041434	0,83215212	0,8487586	0,83215212	0,8487586
Europe (exclud	Male	34,2174621	26,5818805	42,7686694	2009	34,6375406	33,8137953	35,4612858	0	-14,0602716	-14,9516345	-13,1689087	0,71127509	0,69386024	0,72912702	0,69386024	0,72912702
Europe (exclud	Female	53,7189138	45,9480261	61,3132484	2009	50,9594726	49,976452	51,9424931	0,013	-5,75245406	-6,76570905	-4,73919907	0,89856712	0,88097631	0,91650918	0,88097631	0,91650918
Asia	Male	47,8182703	39,470089	56,2901286	2009	49,6226304	48,3612023	50,8840585	0,001	0,92481823	-0,38176985	2,23140631	1,01899096	0,99247657	1,04621369	0,99247657	1,04621369
Asia	Female	84,7749715	77,2913831	90,1079885	2009	83,1301214	82,6146762	83,6455666	0	26,4181948	25,8471957	26,9891939	1,46583138	1,45478587	1,47696074	1,45478587	1,47696074
Middle East, Af	Male	51,3585074	37,8824821	64,6398726	2009	51,0806529	48,5599576	53,6013482	0,441	2,38284071	-0,16075442	4,92643584	1,04893117	0,99793346	1,10253503	0,99793346	1,10253503
Middle East, Af	Female	66,2064022	52,4625727	77,6681079	2009	69,1240768	67,6205731	70,6275805	0,117	12,4121502	10,8887069	13,9355935	1,21886314	1,19212876	1,24619706	1,19212876	1,24619706
Oceania and Ar	Male	52,2254237	38,6254153	65,5033018	2009	52,2763476	50,7105696	53,8421256	0,002	3,57853543	1,97615214	5,18091872	1,07348452	1,04096947	1,10701519	1,04096947	1,10701519
Oceania and Ar	Female	50,0063179	37,120283	62,8915136	2009	57,7243539	55,4382708	60,0104369	0,493	1,01242725	-1,28681871	3,3116732	1,0178521	0,97809837	1,05922159	0,97809837	1,05922159
Australia	Male	49,6158585	48,1385312	51,093857	2010	49,2699886	48,9413258	49,5986513	0	0	-0,46479932	0,46479932	1	0,99061064	1,00947836	0,99061064	1,00947836
Australia	Female	56,4832584	55,1024182	57,8541128	2010	57,1219022	56,8847999	57,3590046	0	0	-0,33531339	0,33531339	1	0,99414706	1,0058874	0,99414706	1,0058874
English speakin	Male	36,9859214	32,6318393	41,5625451	2010	39,4116592	38,7528522	40,0704663	0	-9,85832934	-10,594567	-9,12209165	0,79991208	0,78564414	0,81443914	0,78564414	0,81443914
English speakin	Female	46,4183514	41,8667075	51,0304563	2010	48,0289088	47,6204703	48,4373473	0	-9,09299345	-9,56526409	-8,62072281	0,84081424	0,83289518	0,84880859	0,83289518	0,84880859
Europe (exclud	Male	37,4378088	29,1787305	46,4997184	2010	35,56262	34,7676189	36,3576211	0	-13,7073686	-14,5676278	-12,8471094	0,72179071	0,70514695	0,73882731	0,70514695	0,73882731
Europe (exclud	Female	50,5557989	42,6371759	58,4466354	2010	51,1986464	50,2499279	52,1473649	0,013	-5,92325583	-6,90115376	-4,94535789	0,896305	0,87944533	0,91348788	0,87944533	0,91348788
Asia	Male	55,3593086	46,9393786	63,4825803	2010	50,0701112	48,8527	51,2875224	0,001	0,80012264	-0,46087266	2,06111795	1,01623955	0,99093783	1,04218731	0,99093783	1,04218731
Asia	Female	84,8007952	77,7869618	89,8878872	2010	83,3483212	82,8508623	83,8457802	0	26,226419	25,6753446	26,7774933	1,4591307	1,44856143	1,46977709	1,44856143	1,46977709
Middle East, Af	Male	48,8097866	35,5293418	62,2604145	2010	50,9066694	48,4739326	53,3394062	0,441	1,63668082	-0,81815676	4,09151839	1,03321862	0,98454793	1,08429532	0,98454793	1,08429532
Middle East, Af	Female	67,0860303	53,7389872	78,1481039	2010	68,906691	67,4556513	70,3577306	0,117	11,7847887	10,3145052	13,2550722	1,20630946	1,18069404	1,23248061	1,18069404	1,23248061
Oceania and Ar	Male	50,398111	37,7649773	62,9806156	2010	52,7783221	51,2671812	54,289463	0,002	3,50833357	1,96186469	5,05480245	1,0712063	1,0401727	1,10316579	1,0401727	1,10316579
Oceania and Ar	Female	46,7744171	34,9187088	59,0058899	2010	57,8644679	55,6581567	60,0707792	0,493	0,74256567	-1,47644918	2,96158052	1,01299967	0,97488251	1,05260718	0,97488251	1,05260718
Australia	Male	50,7473039	49,415086	52,0784614	2011	49,8421649	49,5135022	50,1708277	0	0	-0,46479932	0,46479932	1	0,99071792	1,00936904	0,99071792	1,00936904
Australia	Female	57,6041855	56,3661677	58,8327318	2011	57,5318779	57,2947755	57,7689802	0	0	-0,33531339	0,33531339	1	0,99418865	1,00584532	0,99418865	1,00584532
English speakin	Male	40,5163061	36,8772655	44,2626401	2011	39,8750738	39,2162668	40,5338809	0	-9,96709111	-10,7033288	-9,23085342	0,80002692	0,78592103	0,81438599	0,78592103	0,81438599
English speakin	Female	48,378788	44,5584919	52,2181272	2011	48,396301	47,9878625	48,8047395	0	-9,13557685	-9,60784748	-8,66330621	0,84120844	0,83334482	0,84914625	0,83334482	0,84914625
Europe (exclud	Male	36,4107444	29,8043067	43,5725797	2011	36,4876994	35,6926983	37,2827005	0	-13,3544655	-14,2147247	-12,4942063	0,7320649	0,7155883	0,74892087	0,7155883	0,74892087
Europe (exclud	Female	49,9726676	43,4899283	56,456326	2011	51,4378203	50,4891017	52,3865388	0,013	-6,09405759	-7,07195553	-5,11615966	0,89407511	0,87733682	0,91113275	0,87733682	0,91113275
Asia	Male	51,6919105	44,5885064	58,7275913	2011	50,517592	49,3001808	51,7350032	0,001	0,67542706	-0,58556825	1,93642236	1,01355132	0,98854184	1,03919352	0,98854184	1,03919352
Asia	Female	82,08998	76,1688556	86,7949821	2011	83,566521	83,0690621	84,06398	0	26,0346432	25,4835688	26,5857175	1,45252552	1,44204688	1,46308031	1,44204688	1,46308031
Middle East, Af	Male	41,808686	31,8908116	52,4363249	2011	50,7326859	48,2999491	53,1654227	0,441	0,89052093	-1,56431665	3,3453585	1,01786682	0,96977204	1,0683468	0,96977204	1,0683468
Middle East, Af	Female	69,6315771	58,8818589	78,5926858	2011	68,6893051	67,2382655	70,1403447	0,117	11,1574272	9,68714374	12,6277107	1,1939347	1,16851228	1,21991021	1,16851228	1,21991021
Oceania and Ar	Male	57,5339506	46,1371577	68,1823118	2011	53,2802966	51,7691558	54,7914375	0,002	3,43813171	1,89166283	4,98460059	1,06898038	1,03830204	1,10056517	1,03830204	1,10056517
Oceania and Ar	Female	55,9485576	45,4209288	65,9670596	2011	58,0045819	55,7982707	60,2108932	0,493	0,47270409	-1,74631076	2,69171894	1,00821639	0,97037116	1,04753761	0,97037116	1,04753761
Australia	Male	51,3265591	49,9913844	52,6598433	2012	50,4143413	50,0737954	50,7548873	0	0	-0,48160468	0,48160468	1	0,99049255	1,00959871	0,99049255	1,00959871
Australia	Female	58,6863293	57,4597022	59,9022693	2012	57,9418535	57,6961784	58,1875286	0	0	-0,34743704	0,34743704	1	0,99402164	1,00601432	0,99402164	1,00601432
English speakin	Male	42,6837583	39,0180755	46,4316114	2012	40,3384884	39,6558614	41,0211154	0	-10,0758529	-10,8387101	-9,31299566	0,80013915	0,78569196	0,814852	0,78569196	0,814852
English speakin	Female	49,6727976	45,9020552	53,4472657	2012	48,7636932	48,3404871	49,1868993	0	-9,17816024	-9,66750638	-8,6888141	0,84159705	0,83350713	0,8497655	0,83350713	0,8497655
Europe (exclud	Male	34,6751857	27,8505242	42,1941824	2012	37,4127788	36,5890336	38,2365241	0	-13,0015625	-13,8929254	-12,1101996	0,74210587	0,72521001	0,75939538	0,72521001	0,75939538
Europe (exclud	Female	51,3708828	44,6095785	58,0823829	2012	51,6769941	50,6939736	52,6600147	0,013	-6,26485936	-7,27811435	-5,25160437	0,89187679	0,87466309	0,90942925	0,87466309	0,90942925
Asia	Male	51,0868145	43,8879288	58,2409009	2012	50,9650728	49,7036447	52,2265009	0,001	0,55073147	-0,75585661	1,85731955	1,0109241	0,98531767	1,037196	0,98531767	1,037196
Asia	Female	84,9215829	79,5262409	89,0901697	2012	83,7847208	83,2692757	84,300166	0	25,8428674	25,2718683	26,4138665	1,44601382	1,43525001	1,45685835	1,43525001	1,45685835
Middle East, Af	Male	50,0064175	38,8067695	61,2054217	2012	50,5587024	48,0380071	53,0793976	0,441	0,14436103	-2,39923409	2,68795616	1,00286349	0,95365536	1,05461073	0,95365536	1,05461073
Middle East, Af	Female	63,7856265	52,3394321	73,856007	2012	68,4719192	66,9684155	69,9754229	0,117	10,5300658	9,00662242	12,0535091	1,18173505	1,15560054	1,20846061	1,15560054	1,20846061
Oceania and Ar	Male	59,6564085	48,4322891	69,9531317	2012	53,7822712	52,2164932	55,3480491	0,002	3,36792985	1,76554656	4,97031314	1,066805	1,0353935	1,09916945	1,0353935	1,09916945
Oceania and Ar	Female	61,1778578	50,9763042	70,4856383	2012	58,144696	55,8586129	60,430779	0,493	0,20284251	-2,09640344	2,50208846	1,00350079	0,96459166	1,04397943	0,96459166	1,04397943
Australia	Male	51,4603237	50,1200155	52,7985347	2013	50,9865177	50,6233701	51,3496653	0	0	-0,51356831	0,51356831	1	0,98997793	1,01012353	0,98997793	1,01012353
Australia	Female	58,5514994	57,3224896	59,7699559	2013	58,3518291	58,0898488	58,6138094	0	0	-0,37049609	0,37049609	1	0,99367077	1,00636955	0,99367077	1,00636955
English speakin	Male	39,8253681	36,230937	43,5329605	2013	40,801903	40,0739708	41,5298353	0	-10,1846147	-10,998102	-9,37112735	0,80024887	0,78502294	0,81577011	0,78502294	0,81577011
English speakin	Female	48,9810606	45,1744553	52,7995193	2013	49,1310854	48,6797916	49,5823793	0	-9,22074364	-9,7425672	-8,69892008	0,84198021	0,83341564	0,85063279	0,83341564	0,85063279
Europe (exclud	Male	39,4309021	32,5732706	46,7316541	2013	38,3378583	37,4594418	39,2162747	0	-12,6486594	-13,5991812	-11,6981377	0,75192149	0,73409461	0,77018128	0,73409461	0,77018128
Europe (exclud	Female	54,2989475	47,765672	60,6876074	2013	51,916168	50,8679053	52,9644306	0,013	-6,43566113	-7,51616484	-5,35515741	0,88970935	0,87149512	0,90830426	0,87149512	0,90830426
Asia	Male	52,8255322	45,6055961	59,9291648	2013	51,4125536	50,0674057	52,7577014	0,001	0,42603588	-0,96726915	1,81934091	1,00835585	0,98138073	1,03607244	0,98138073	1,03607244
Asia	Female	84,1635525	78,9207523	88,2957563	2013	84,0029206	83,4532659	84,5525754	0	25,6510916	25,0421958	26,2599873	1,43959362	1,42821497	1,45106291	1,42821497	1,45106291
Middle East, Af	Male	47,7954026	37,2123566	58,5801387	2013	50,3847188	47,6967275	53,0727101	0,441	-0,60179886	-3,31420987	2,11061215	0,9881969	0,93641551	1,04284168	0,93641551	1,04284168
Middle East, Af	Female	69,145993	58,5278585	78,0644477	2013	68,2545334	66,6512436	69,8578231	0,117	9,90270429	8,27815152	11,5272571	1,16970684	1,14206505	1,19801764	1,14206505	1,19801764
Oceania and Ar	Male	54,2878156	43,4204771	64,7618147	2013	54,2842457	52,6145486	55,9539428	0,002	3,29772799	1,58899611	5,00645986	1,06467843	1,03158921	1,09882902	1,03158921	1,09882902
Oceania and Ar	Female	62,6544494	52,5238867	71,7845688	2013	58,28481	55,8470019	60,7226181	0,493	-0,06701907	-2,51886369	2,38482555	0,99885147	0,95770521	1,0417655	0,95770521	1,0417655
Australia	Male	51,5042257	50,185836	52,8205253	2014	51,5586941	51,1640636	51,9533245	0	0	-0,55809176	0,55809176	1	0,98923398	1,01088319	0,98923398	1,01088319
Australia	Female	58,7653054	57,5461063	59,9738448	2014	58,7618047	58,4771122	59,0464972	0	0	-0,40261599	0,40261599	1	0,99317176	1,00687519	0,99317176	1,00687519
English speakin	Male	41,78969	38,1383519	45,5332986	2014	41,2653176	40,4742778	42,0563575	0	-10,2933764	-11,1773885	-9,40936441	0,80035615	0,7840052	0,8170481	0,7840052	0,8170481
English speakin	Female	49,5207201	45,7387827	53,30815	2014	49,4984777	49,0080592	49,9888961	0	-9,26332704	-9,83038973	-8,69626434	0,84235802	0,8331188	0,85169971	0,8331188	0,85169971
Europe (exclud	Male	38,0221578	30,8772885	45,7266197	2014	39,2629377	38,3083675	40,2175078	0	-12,2957564	-13,328683	-11,2628298	0,76151924	0,74235446	0,78117879	0,74235446	0,78117879
Europe (exclud	Female	51,8584834	44,9654544	58,6814902	2014	52,1553418	51,0162007	53,2944829	0,013	-6,60646289	-7,78064013	-5,43228565	0,88757216	0,86793569	0,90765289	0,86793569	0,90765289
Asia	Male	52,4384341	45,2847698	59,4934114	2014	51,8600344	50,3982699	53,3217989	0,001	0,30134029	-1,21275636	1,81543694	1,00584461	0,97689133	1,035656	0,97689133	1,035656
Asia	Female	83,4921192	77,7492241	87,9818975	2014	84,2211204	83,6238137	84,8184272	0	25,4593158	24,7976322	26,1209993	1,433263	1,42100542	1,44562631	1,42100542	1,44562631
Middle East, Af	Male	55,2460822	44,5448603	65,482455	2014	50,2107353	47,2897105	53,1317602	0,441	-1,34795875	-4,29552036	1,59960286	0,97385584	0,91835739	1,03270819	0,91835739	1,03270819
Middle East, Af	Female	63,1938868	52,2077221	72,9625016	2014	68,0371475	66,2948617	69,7794333	0,117	9,27534281	7,50995056	11,0407351	1,15784646	1,12806033	1,18841908	1,12806033	1,18841908
Oceania and Ar	Male	57,9915454	47,4171697	67,8798989	2014	54,7862202	52,9717698	56,6006705	0,002	3,22752613	1,37065692	5,08439533	1,06259907	1,02708652	1,09933949	1,02708652	1,09933949
Oceania and Ar	Female	59,8127276	50,1943509	68,7307648	2014	58,424924	55,7757718	61,0740762	0,493	-0,33688065	-3,00128624	2,32752495	0,99426701	0,94994573	1,04065618	0,94994573	1,04065618
Australia	Male	52,3338901	51,0116245	53,6528921	2015	52,1308704	51,6978086	52,5639323	0	0	-0,6124419	0,6124419	1	0,98832058	1,01181744	0,98832058	1,01181744
Australia	Female	59,1177588	57,9024529	60,322022	2015	59,1717803	58,8593628	59,4841978	0	0	-0,44182502	0,44182502	1	0,99256099	1,00749477	0,99256099	1,00749477
English speakin	Male	41,2633786	37,5551179	45,0735006	2015	41,7287322	40,8606564	42,596808	0	-10,4021382	-11,3722403	-9,43203607	0,80046107	0,78272994	0,81859386	0,78272994	0,81859386
English speakin	Female	50,2871462	46,4214021	54,1494603	2015	49,8658699	49,3276917	50,4040481	0	-9,30591043	-9,92819691	-8,68362396	0,8427306	0,83266595	0,8529169	0,83266595	0,8529169
Europe (exclud	Male	40,4114218	33,0970152	48,1782462	2015	40,1880171	39,1404855	41,2355487	0	-11,9428533	-13,0763722	-10,8093345	0,77090631	0,7501021	0,79228753	0,7501021	0,79228753
Europe (exclud	Female	53,7822456	46,9275328	60,4968887	2015	52,3945156	51,1444386	53,6445927	0,013	-6,77726466	-8,06578994	-5,48873938	0,88546458	0,86408947	0,90736846	0,86408947	0,90736846
Asia	Male	51,6562285	44,4522172	58,7920522	2015	52,3075151	50,7033957	53,9116346	0,001	0,1766447	-1,48490327	1,83819268	1,00338849	0,97200964	1,03578032	0,97200964	1,03578032
Asia	Female	84,1341855	78,6276766	88,4307131	2015	84,4393203	83,7838444	85,0947961	0	25,26754	24,5414179	25,993662	1,42702011	1,41368582	1,44048016	1,41368582	1,44048016
Middle East, Af	Male	50,7710009	40,2873888	61,1872424	2015	50,0367518	46,831261	53,2422426	0,441	-2,09411864	-5,32873053	1,14049324	0,95982959	0,89978573	1,02388024	0,89978573	1,02388024
Middle East, Af	Female	65,9503765	54,300219	75,9462048	2015	67,8197616	65,9078021	69,7317212	0,117	8,64798133	6,71066517	10,5852975	1,14615043	1,1137436	1,17950022	1,1137436	1,17950022
Oceania and Ar	Male	53,3574169	42,8801533	63,5466444	2015	55,2881947	53,2970429	57,2793465	0,002	3,15732426	1,1196226	5,19502593	1,06056535	1,02208268	1,10049694	1,02208268	1,10049694
Oceania and Ar	Female	58,4001555	48,4406595	67,71791	2015	58,5650381	55,6578964	61,4721798	0,493	-0,60674222	-3,53062276	2,31713831	0,98974609	0,94155135	1,04040774	0,94155135	1,04040774
Australia	Male	52,2570226	50,974587	53,5364895	2016	52,7030468	52,2262825	53,1798111	0	0	-0,67424656	0,67424656	1	0,98728817	1,0128755	0,98728817	1,0128755
Australia	Female	59,3980904	58,2126022	60,5727256	2016	59,5817559	59,2378108	59,925701	0	0	-0,48641186	0,48641186	1	0,99186946	1,00819719	0,99186946	1,00819719
English speakin	Male	43,1581663	39,4272444	46,9683816	2016	42,1921468	41,236469	43,1478246	0	-10,5109	-11,5789001	-9,44289986	0,80056371	0,78127396	0,82032973	0,78127396	0,82032973
English speakin	Female	51,7387196	47,8820347	55,5748008	2016	50,2332621	49,6407736	50,8257506	0	-9,34849383	-10,0335784	-8,66340923	0,84309805	0,83209917	0,85424231	0,83209917	0,85424231
Europe (exclud	Male	39,9035201	32,8030844	47,4554838	2016	41,1130965	39,9598531	42,2663399	0	-11,5899503	-12,8378583	-10,3420422	0,78008956	0,75743339	0,80342341	0,75743339	0,80342341
Europe (exclud	Female	54,1077749	47,2977801	60,7677132	2016	52,6336895	51,2574607	54,0099183	0,013	-6,94806643	-8,36662341	-5,52950944	0,88338601	0,86004552	0,90735993	0,86004552	0,90735993
Asia	Male	50,3673023	43,4948243	57,2259291	2016	52,7549959	50,9889966	54,5209953	0,001	0,05194912	-1,7772742	1,88117243	1,00098569	0,96687017	1,03630496	0,96687017	1,03630496
Asia	Female	83,9335994	78,9932777	87,8901801	2016	84,6575201	83,9358968	85,3791433	0	25,0757641	24,2763654	25,8751629	1,42086313	1,40631068	1,43556617	1,40631068	1,43556617
Middle East, Af	Male	55,0571026	44,2442379	65,4122934	2016	49,8627683	46,3337949	53,3917417	0,441	-2,84027853	-6,40131171	0,72075465	0,94610789	0,88095548	1,01607874	0,88095548	1,01607874
Middle East, Af	Female	69,8145865	58,8591087	78,8986027	2016	67,6023758	65,4974706	69,7072809	0,117	8,02061985	5,88779917	10,1534405	1,13461537	1,09924827	1,17112036	1,09924827	1,17112036
Oceania and Ar	Male	59,485322	48,9668111	69,1995267	2016	55,7901692	53,5980801	57,9822584	0,002	3,0871224	0,8437858	5,33045901	1,05857579	1,01674343	1,10212927	1,01674343	1,10212927
Oceania and Ar	Female	59,5665833	49,6976627	68,7180346	2016	58,7051521	55,5046358	61,9056684	0,493	-0,8766038	-4,09554814	2,34234053	0,98528738	0,93272471	1,04081216	0,93272471	1,04081216
Australia	Male	53,2406571	51,9526041	54,5244051	2017	53,2752232	52,7508014	53,799645	0	0	-0,74164441	0,74164441	1	0,98617545	1,01401835	0,98617545	1,01401835
Australia	Female	59,9590789	58,7737166	61,1328975	2017	59,9917315	59,6134056	60,3700575	0	0	-0,5350337	0,5350337	1	0,99112119	1,00895835	0,99112119	1,00895835
English speakin	Male	42,2966578	38,6611372	46,0175707	2017	42,6555614	41,6043539	43,706769	0	-10,6196618	-11,7944194	-9,44490408	0,80066415	0,77969613	0,82219605	0,77969613	0,82219605
English speakin	Female	49,7276537	45,9471227	53,5113014	2017	50,6006543	49,9489405	51,2523681	0	-9,39107723	-10,144643	-8,6375114	0,84346047	0,83145108	0,85564334	0,83145108	0,85564334
Europe (exclud	Male	43,1129808	35,660122	50,8914178	2017	42,038176	40,7696541	43,3066978	0	-11,2370472	-12,6096965	-9,86439799	0,78907555	0,76442324	0,81452288	0,76442324	0,81452288
Europe (exclud	Female	50,3944231	43,47476	57,2990096	2017	52,8728633	51,3590664	54,3866603	0,013	-7,11886819	-8,67922448	-5,55851191	0,88133584	0,85587264	0,90755661	0,85587264	0,90755661
Asia	Male	52,2691243	45,3933204	59,0599921	2017	53,2024767	51,2599476	55,1450058	0,001	-0,07274647	-2,08481939	1,93932645	0,99863452	0,96157562	1,03712165	0,96157562	1,03712165
Asia	Female	85,098979	80,4477707	88,7978007	2017	84,8757199	84,081963	85,6694768	0	24,8839883	24,0046815	25,7632952	1,4147903	1,39892171	1,4308389	1,39892171	1,4308389
Middle East, Af	Male	49,0110388	38,1787116	59,9370408	2017	49,6887848	45,8070544	53,5705152	0,441	-3,58643843	-7,50343335	0,3305565	0,93268093	0,86205974	1,00908751	0,86205974	1,00908751
Middle East, Af	Female	70,3036851	59,3506175	79,3331258	2017	67,3849899	65,0696779	69,7003019	0,117	7,39325837	5,04724042	9,73927633	1,12323796	1,0846769	1,16316989	1,0846769	1,16316989
Oceania and Ar	Male	56,0322463	45,7130319	65,8550742	2017	56,2921437	53,8809328	58,7033547	0,002	3,01692054	0,54933946	5,48450162	1,05662896	1,01119541	1,10410386	1,01119541	1,10410386
Oceania and Ar	Female	61,6688548	51,8102139	70,6530429	2017	58,8452661	55,3248255	62,3657068	0,493	-1,14646538	-4,68717617	2,39424541	0,98088961	0,92362217	1,04170781	0,92362217	1,04170781
Australia	Male	53,5004793	52,2120513	54,7842525	2018	53,8473996	53,2723478	54,4224513	0	0	-0,81324603	0,81324603	1	0,98501068	1,01521741	0,98501068	1,01521741
Australia	Female	60,6219311	59,4299641	61,8013871	2018	60,4017071	59,9868559	60,8165584	0	0	-0,58668822	0,58668822	1	0,99033391	1,00976043	0,99033391	1,00976043
English speakin	Male	42,8263888	39,0255661	46,7137874	2018	43,118976	41,9662802	44,2716718	0	-10,7284235	-12,0165975	-9,44024957	0,80076246	0,77803948	0,82414907	0,77803948	0,82414907
English speakin	Female	51,0580043	47,1743864	54,9288924	2018	50,9680465	50,2534134	51,6826797	0	-9,43366062	-10,259979	-8,60734223	0,84381798	0,83074575	0,85709591	0,83074575	0,85709591
Europe (exclud	Male	45,0975262	37,6913963	52,727431	2018	42,9632554	41,5722648	44,3542459	0	-10,8841442	-12,389315	-9,37897333	0,79787057	0,77112794	0,82554063	0,77112794	0,82554063
Europe (exclud	Female	52,9935017	45,9607951	59,9093136	2018	53,1120372	51,4520916	54,7719828	0,013	-7,28966996	-9,00066992	-5,57867	0,87931351	0,85162141	0,90790608	0,85162141	0,90790608
Asia	Male	51,4868018	44,9348474	57,9880461	2018	53,6499575	51,5198881	55,7800269	0,001	-0,19744206	-2,40376937	2,00888525	0,9963333	0,95620046	1,03815057	0,95620046	1,03815057
Asia	Female	85,1428936	80,6102883	88,7636346	2018	85,0939197	84,22353	85,9643094	0	24,6922125	23,7280135	25,6564115	1,40879991	1,39154921	1,42626447	1,39154921	1,42626447
Middle East, Af	Male	47,4930717	37,0274456	58,1836506	2018	49,5148012	45,2583115	53,771291	0,441	-4,33259832	-8,62775719	-0,03743944	0,91953932	0,84323697	1,00274608	0,84323697	1,00274608
Middle East, Af	Female	69,9664374	59,1581219	78,9330471	2018	67,167604	64,6287616	69,7064465	0,117	6,7658969	4,19338403	9,33840976	1,11201499	1,07010432	1,1555671	1,07010432	1,1555671
Oceania and Ar	Male	53,176893	42,6240161	63,4530435	2018	56,7941182	54,1501184	59,4381181	0,002	2,94671868	0,24090645	5,65253092	1,05472351	1,0055306	1,10632306	1,0055306	1,10632306
Oceania and Ar	Female	60,9237594	50,7098438	70,2623374	2018	58,9853802	55,1250607	62,8456997	0,493	-1,41632696	-5,29887358	2,46621966	0,97655154	0,91435853	1,0429748	0,91435853	1,0429748
Australia	Male	53,4767578	52,1907654	54,7581445	2019	54,4195759	53,7916403	55,0475116	0	0	-0,88803516	0,88803516	1	0,98381412	1,01645217	0,98381412	1,01645217
Australia	Female	60,449838	59,2656751	61,6218684	2019	60,8116828	60,3586803	61,2646852	0	0	-0,64064225	0,64064225	1	0,98952044	1,01059054	0,98952044	1,01059054
English speakin	Male	44,1908559	40,284209	48,1707481	2019	43,5823906	42,3236887	44,8410926	0	-10,8371853	-12,2438245	-9,43054607	0,8008587	0,77633475	0,82615734	0,77633475	0,82615734
English speakin	Female	50,8364968	46,8762395	54,7862835	2019	51,3354387	50,5550852	52,1157922	0	-9,47624402	-10,3785537	-8,57393432	0,84417067	0,83000065	0,8585826	0,83000065	0,8585826
Europe (exclud	Male	42,6052942	34,9835166	50,5953906	2019	43,8883348	42,3694236	45,407246	0	-10,5312411	-12,1748331	-8,88764916	0,80648065	0,77758928	0,83644547	0,77758928	0,83644547
Europe (exclud	Female	53,4703417	46,3122764	60,4882665	2019	53,351211	51,5386107	55,1638114	0,013	-7,46047172	-9,32882161	-5,59212184	0,87731845	0,84732819	0,90837017	0,84732819	0,90837017
Asia	Male	55,7006693	49,4189943	61,8051609	2019	54,0974383	51,7714798	56,4233968	0,001	-0,32213765	-2,73136702	2,08709173	0,99408048	0,95079743	1,03933391	0,95079743	1,03933391
Asia	Female	85,7684458	81,9243218	88,905756	2019	85,3121195	84,3616855	86,2625534	0	24,5004367	23,4475664	25,5533071	1,40289029	1,38421458	1,42181798	1,38421458	1,42181798
Middle East, Af	Male	46,1867533	36,3485073	56,3312423	2019	49,3408177	44,6928853	53,9887501	0,441	-5,07875821	-9,76891588	-0,38860054	0,90667406	0,82458349	0,99693707	0,82458349	0,99693707
Middle East, Af	Female	68,8350312	57,9415095	77,9793513	2019	66,9502182	64,1778943	69,722542	0,117	6,13853542	3,32944469	8,94762614	1,10094336	1,05558378	1,14825208	1,05558378	1,14825208
Oceania and Ar	Male	54,7112874	44,7208156	64,3360964	2019	57,2960928	54,4089408	60,1832447	0,002	2,87651682	-0,07813198	5,83116562	1,05285813	0,99981423	1,1087162	0,99981423	1,1087162
Oceania and Ar	Female	58,0090312	47,9706064	67,4258324	2019	59,1254942	54,9101654	63,3408231	0,493	-1,68618854	-5,92578859	2,55341152	0,97227196	0,9050162	1,0445258	0,9050162	1,0445258

Subpopulation	Sex	Prevalence(P)	P_LCI	P_UCI	Year	Linear(lr)	lr_LCI	lr_UCI	p_value	Difference(D)	D_LCI	D_UCI	Ratio(R)	R_LCI	R_UCI
Australia	Male	26,9606976	25,6681662	28,2935407	2002	26,2765488	25,7427422	26,8103554	0	0	-0,75491658	0,75491658	1	0,9716791	1,02914635
Australia	Female	22,3637152	21,226656	23,5434794	2002	21,858641	21,3296669	22,387615	0	0	-0,74808228	0,74808228	1	0,96635536	1,03481601
English speakin	Male	28,0538082	24,3022801	32,138583	2002	28,2920013	26,8228524	29,7611503	0	2,01545256	0,45233123	3,57857389	1,07670157	1,01830744	1,13844427
English speakin	Female	23,4234641	19,8854202	27,375898	2002	22,2807974	21,1760422	23,3855526	0	0,4221564	-0,80270995	1,64702275	1,01931302	0,96459712	1,07713263
Europe (exclud	Male	27,7420995	22,4263963	33,7692864	2002	26,3569522	23,1954803	29,5184241	0	0,08040344	-3,1258177	3,28662459	1,00305989	0,8881619	1,13282179
Europe (exclud	Female	19,1957415	14,7444011	24,6031308	2002	18,5560411	16,6785597	20,4335224	0,095	-3,30259994	-5,25317675	-1,35202313	0,84891101	0,76503502	0,94198291
Asia	Male	19,7857929	14,4820878	26,4315149	2002	21,9088942	19,7177437	24,1000446	0,011	-4,36765463	-6,6228907	-2,11241855	0,83378127	0,75288824	0,92336573
Asia	Female	10,4813771	6,29763908	16,9419828	2002	9,95144452	8,41221201	11,490677	0	-11,9071965	-13,5347868	-10,2796061	0,45526364	0,38928851	0,53241998
Middle East, Af	Male	29,2186894	19,8847705	40,7077276	2002	25,6824941	22,1552245	29,2097637	0,686	-0,59405467	-4,16148796	2,97337862	0,97739221	0,85069384	1,12296045
Middle East, Af	Female	16,499469	9,38281397	27,3828263	2002	20,1658918	17,4406521	22,8911316	0,21	-1,69274916	-4,46885167	1,08335334	0,92255927	0,80421041	1,05832453
Oceania and Ar	Male	36,6428864	25,2202532	49,7941001	2002	29,766422	25,6077138	33,9251301	0	3,48987317	-0,70295442	7,68270077	1,13281322	0,98365849	1,30458469
Oceania and Ar	Female	20,183361	11,9038142	32,1218759	2002	21,3589354	18,8523005	23,8655702	0,003	-0,49970564	-3,06154716	2,06213588	0,97713922	0,8667949	1,10153054
Australia	Male	26,6275924	25,3262098	27,970798	2003	25,7757536	25,2869035	26,2646038	0	0	-0,69133852	0,69133852	1	0,97353522	1,0271842
Australia	Female	21,9530732	20,8174934	23,1325014	2003	21,3782726	20,893848	21,8626972	0	0	-0,68507979	0,68507979	1	0,96846241	1,0325646
English speakin	Male	26,5248501	22,7001652	30,7377003	2003	27,7491134	26,4036941	29,0945326	0	1,97335976	0,54188241	3,40483711	1,07655876	1,0219445	1,1340917
English speakin	Female	21,6712427	18,1173391	25,7034465	2003	21,771812	20,7600979	22,7835262	0	0,39353944	-0,72817031	1,51524919	1,01840838	0,96709514	1,07244426
Europe (exclud	Male	25,3577112	19,8146025	31,8358351	2003	25,6415181	22,7463011	28,5367351	0	-0,13423553	-3,07043302	2,80196197	0,99479218	0,88717374	1,11546525
Europe (exclud	Female	17,9250875	13,0838139	24,0618084	2003	18,3980582	16,6786957	20,1174206	0,095	-2,98021445	-4,76651629	-1,19391261	0,86059611	0,78169443	0,94746186
Asia	Male	20,2014168	14,0905551	28,0957518	2003	21,6083927	19,601778	23,6150074	0,011	-4,16736092	-6,23266404	-2,10205781	0,83832244	0,76251575	0,92166557
Asia	Female	8,69457199	4,83357916	15,1487372	2003	9,50372337	8,09412296	10,9133238	0	-11,8745492	-13,3650661	-10,3840324	0,44455057	0,38261224	0,51651566
Middle East, Af	Male	22,3258546	13,4430783	34,7234822	2003	25,7513684	22,5211607	28,9815761	0,686	-0,02438522	-3,29137406	3,24260362	0,99905395	0,88002019	1,13418851
Middle East, Af	Female	14,7642042	7,73590607	26,3540091	2003	19,9971363	17,5014126	22,49286	0,21	-1,38113629	-3,92343916	1,16116659	0,93539533	0,82396233	1,06189857
Oceania and Ar	Male	28,0655642	17,9889532	40,9670572	2003	28,8332536	25,0247862	32,6417209	0	3,05749994	-0,78221333	6,89721321	1,11861923	0,97888072	1,27830587
Oceania and Ar	Female	19,2565741	10,8151518	31,927948	2003	20,9357187	18,6401892	23,2312481	0,003	-0,44255393	-2,78864059	1,90353273	0,97929889	0,8755685	1,09531844
Australia	Male	25,398254	24,0999941	26,74181	2004	25,2749584	24,8291487	25,7207682	0	0	-0,63047015	0,63047015	1	0,97536408	1,02525817
Australia	Female	21,4032919	20,2683709	22,5837621	2004	20,8979042	20,4561304	21,339678	0	0	-0,62476247	0,62476247	1	0,97054652	1,03034731
English speakin	Male	25,7852646	22,0058245	29,9644333	2004	27,2062254	25,9792626	28,4331882	0	1,93126696	0,62582294	3,23671099	1,07641029	1,0255269	1,12981836
English speakin	Female	20,7130065	17,2685976	24,6398241	2004	21,2628267	20,3401881	22,1854653	0	0,36492247	-0,65802721	1,38787215	1,01746216	0,96951812	1,0677771
Europe (exclud	Male	27,856556	21,9061406	34,7049998	2004	24,9260839	22,2857741	27,5663938	0	-0,34887449	-3,02655674	2,32880776	0,98619683	0,88578289	1,09799387
Europe (exclud	Female	17,8509267	13,041618	23,9453816	2004	18,2400753	16,6720928	19,8080577	0,095	-2,65782895	-4,28685722	-1,02880068	0,8728184	0,79887366	0,95360755
Asia	Male	26,5450452	19,019784	35,7338802	2004	21,3078912	19,4779474	23,1378351	0,011	-3,96706721	-5,85053231	-2,08360212	0,84304357	0,77227833	0,92029316
Asia	Female	8,85344855	4,86011747	15,5902449	2004	9,05600222	7,77050899	10,3414954	0	-11,841902	-13,2011874	-10,4826166	0,433345	0,37541024	0,50022047
Middle East, Af	Male	18,0966451	10,3071368	29,8160646	2004	25,8202427	22,8744361	28,7660492	0,686	0,54528423	-2,4340651	3,52463355	1,02157409	0,91019193	1,14658632
Middle East, Af	Female	24,9206563	15,2479303	37,9796871	2004	19,8283808	17,5523912	22,1043704	0,21	-1,06952341	-3,38799118	1,24894437	0,9488215	0,84429829	1,06628457
Oceania and Ar	Male	25,6936484	15,1903672	40,0313844	2004	27,9000851	24,4269314	31,3732389	0	2,6251267	-0,87652197	6,12677537	1,10386275	0,97344575	1,25175232
Oceania and Ar	Female	20,4204612	11,4686212	33,6998923	2004	20,512502	18,4190807	22,6059233	0,003	-0,38540223	-2,52492949	1,75412504	0,98155785	0,88440815	1,08937917
Australia	Male	24,7820675	23,5013634	26,1087436	2005	24,7741633	24,3688671	25,1794595	0	0	-0,5731754	0,5731754	1	0,97712957	1,02340573
Australia	Female	21,242818	20,1159634	22,4150843	2005	20,4175358	20,0159088	20,8191629	0	0	-0,56798641	0,56798641	1	0,97256481	1,02820911
English speakin	Male	27,8886157	23,9165339	32,2408381	2005	26,6633374	25,5478764	27,7787985	0	1,88917416	0,70236397	3,07598435	1,07625582	1,02898018	1,12570351
English speakin	Female	21,3102129	17,6725235	25,4650721	2005	20,7538413	19,9150487	21,592634	0	0,33630551	-0,59368236	1,26629337	1,01647141	0,97179369	1,06320316
Europe (exclud	Male	29,7831547	23,2337334	37,2821715	2005	24,2106498	21,8102814	26,6110182	0	-0,56351346	-2,99785804	1,87083111	0,97725399	0,8838265	1,0805575
Europe (exclud	Female	21,4184718	16,030599	28,0130826	2005	18,0820924	16,6566022	19,5075825	0,095	-2,33544346	-3,81643178	-0,85445514	0,8856158	0,816504	0,96057747
Asia	Male	22,9947751	15,9892635	31,9040372	2005	21,0073898	19,3437443	22,6710352	0,011	-3,76677351	-5,47907639	-2,05447063	0,84795557	0,78208449	0,91937465
Asia	Female	7,91250622	4,08514709	14,7733911	2005	8,60828106	7,43960861	9,77695351	0	-11,8092548	-13,0450135	-10,5734961	0,42161214	0,36756741	0,48360327
Middle East, Af	Male	30,7814628	20,2754625	43,7442985	2005	25,8891169	23,2110142	28,5672197	0,686	1,11495367	-1,59364361	3,82355095	1,0450047	0,94109671	1,16038533
Middle East, Af	Female	21,5226987	12,5898561	34,3061049	2005	19,6596253	17,590469	21,7287816	0,21	-0,75791053	-2,86568469	1,34986363	0,96287943	0,86511009	1,07169806
Oceania and Ar	Male	30,8715436	19,2917072	45,4849023	2005	26,9669167	23,8093901	30,1244434	0	2,19275346	-0,99067857	5,3761855	1,08850969	0,96713542	1,22511628
Oceania and Ar	Female	21,1693903	12,2538585	34,0541253	2005	20,0892853	18,1861062	21,9924644	0,003	-0,32825052	-2,27334561	1,61684456	0,98392311	0,89318265	1,0838821
Australia	Male	23,6932411	22,4511488	24,9819143	2006	24,2733681	23,9052233	24,6415129	0	0	-0,52063541	0,52063541	1	0,97877956	1,02168051
Australia	Female	19,4047163	18,330969	20,5255518	2006	19,9371674	19,5723555	20,3019794	0	0	-0,51592206	0,51592206	1	0,97445455	1,02621513
English speakin	Male	25,6007637	21,7425024	29,8822497	2006	26,1204495	25,1072369	27,133662	0	1,84708136	0,76905986	2,92510287	1,07609498	1,03219654	1,12186037
English speakin	Female	19,0889348	15,7424226	22,9530501	2006	20,244856	19,4829511	21,0067609	0	0,30768855	-0,53705219	1,15242929	1,01543291	0,97381687	1,05882741
Europe (exclud	Male	22,3798937	16,5627301	29,5173785	2006	23,4952157	21,3148765	25,6755548	0	-0,77815243	-2,98935336	1,4330485	0,96794213	0,88107417	1,06337469
Europe (exclud	Female	21,0959545	15,9041596	27,4297674	2006	17,9241095	16,6292866	19,2189324	0,095	-2,01305797	-3,35829183	-0,6678241	0,89902989	0,83446908	0,96858561
Asia	Male	19,3274776	12,8397465	28,0388044	2006	20,7068883	19,1957405	22,2180361	0,011	-3,5664798	-5,12182482	-2,01113477	0,85307025	0,7917966	0,9190856
Asia	Female	8,87572526	4,32078623	17,3611541	2006	8,16055991	7,09901355	9,22210626	0	-11,7766075	-12,8990907	-10,6541244	0,40931391	0,35892705	0,46677416
Middle East, Af	Male	23,9275978	14,7074403	36,457255	2006	25,9579912	23,5253762	28,3906063	0,686	1,68462312	-0,77569121	4,14493745	1,06940212	0,97255061	1,17589858
Middle East, Af	Female	20,1499559	11,9089707	32,0207603	2006	19,4908698	17,6113823	21,3703573	0,21	-0,44629765	-2,36086317	1,46826787	0,97761479	0,88622056	1,07843434
Oceania and Ar	Male	26,0956439	15,2244364	40,9773643	2006	26,0337483	23,1656556	28,901841	0	1,76038023	-1,13124323	4,65200369	1,07252311	0,95964351	1,19868037
Oceania and Ar	Female	22,0306008	12,7728545	35,2840975	2006	19,6660686	17,937344	21,3947932	0,003	-0,27109882	-2,03789721	1,49569957	0,98640234	0,90169482	1,0790675
Australia	Male	23,759894	22,5019515	25,0654152	2007	23,7725729	23,4370985	24,1080473	0	0	-0,47443248	0,47443248	1	0,98024069	1,02015761
Australia	Female	19,1038279	18,030614	20,2251597	2007	19,4567991	19,1243617	19,7892364	0	0	-0,47013741	0,47013741	1	0,97612645	1,02445744
English speakin	Male	24,2567959	20,2010305	28,8326271	2007	25,5775615	24,6542648	26,5008582	0	1,80498856	0,82263429	2,78734283	1,07592735	1,03502406	1,11844711
English speakin	Female	17,7281429	14,4710382	21,5337744	2007	19,7358706	19,0415797	20,4301616	0	0,27907158	-0,49070404	1,0488472	1,01434314	0,97543907	1,05479884
Europe (exclud	Male	21,9504178	15,9202232	29,4642842	2007	22,7797815	20,7929329	24,7666301	0	-0,9927914	-3,00776297	1,02218017	0,95823795	0,87720618	1,04675502
Europe (exclud	Female	14,4171033	9,96033248	20,4158956	2007	17,7661266	16,5862106	18,9460426	0,095	-1,69067247	-2,91652578	-0,46481916	0,91310634	0,8525876	0,97792085
Asia	Male	19,7367004	13,5312716	27,8706714	2007	20,4063868	19,0293434	21,7834303	0,011	-3,36618609	-4,7835046	-1,94886758	0,85840043	0,80121548	0,91966683
Asia	Female	8,82755037	4,56248668	16,3947316	2007	7,71283876	6,74549758	8,68017994	0	-11,7439603	-12,7668306	-10,7210901	0,39640841	0,34927758	0,44989898
Middle East, Af	Male	28,2793561	18,2535479	41,0468208	2007	26,0268655	23,8101288	28,2436021	0,686	2,25429257	0,01231476	4,49627037	1,09482745	1,00427362	1,19354639
Middle East, Af	Female	23,4454379	14,4593553	35,6863058	2007	19,3221143	17,6094188	21,0348098	0,21	-0,13468477	-1,87934535	1,6099758	0,99307775	0,90735894	1,08689448
Oceania and Ar	Male	22,4762508	12,9023239	36,201539	2007	25,1005799	22,4870114	27,7141485	0	1,32800699	-1,30700413	3,96301812	1,05586299	0,95054732	1,17284708
Oceania and Ar	Female	22,3160798	13,228228	35,12035	2007	19,2428519	17,6675401	20,8181637	0,003	-0,21394711	-1,8239539	1,39605968	0,98900399	0,90965882	1,07527007
Australia	Male	22,6754921	21,4332432	23,967776	2008	23,2717778	22,9630668	23,5804887	0	0	-0,4365832	0,4365832	1	0,98141468	1,01893728
Australia	Female	18,3230532	17,2589133	19,4373917	2008	18,9764307	18,6705145	19,2823468	0	0	-0,43263079	0,43263079	1	0,9774596	1,02306019
English speakin	Male	26,551158	22,249606	31,3490238	2008	25,0346735	24,1850356	25,8843115	0	1,76289576	0,85891176	2,66687976	1,07575252	1,03725891	1,11567466
English speakin	Female	17,9708882	14,5295103	22,0173985	2008	19,2268853	18,5879835	19,8657871	0	0,25045462	-0,45790984	0,95881907	1,01319819	0,97645979	1,05131885
Europe (exclud	Male	17,5968802	11,7436099	25,5238336	2008	22,0643474	20,2360056	23,8926891	0	-1,20743037	-3,06165151	0,64679078	0,94811611	0,87179805	1,03111513
Europe (exclud	Female	15,8915137	10,6714758	23,0073191	2008	17,6081437	16,5223591	18,6939283	0,095	-1,36828698	-2,49634415	-0,24022981	0,92789545	0,87060021	0,98896136
Asia	Male	20,9514221	14,1946269	29,8072375	2008	20,1058854	18,8386997	21,373071	0,011	-3,16589238	-4,47014004	-1,86164473	0,86396001	0,81006943	0,92143571
Asia	Female	10,995936	6,0314843	19,2111844	2008	7,2651176	6,37494898	8,15528623	0	-11,7113131	-12,6525808	-10,7700454	0,38284953	0,33834284	0,43321078
Middle East, Af	Male	27,3335429	17,0542908	40,763615	2008	26,0957398	24,05585	28,1356296	0,686	2,82396202	0,76084474	4,88707929	1,12134707	1,03587207	1,21387504
Middle East, Af	Female	21,140997	12,1522752	34,1907292	2008	19,1533588	17,5772987	20,7294188	0,21	0,1769281	-1,42854692	1,78240313	1,00932357	0,92814228	1,0976055
Oceania and Ar	Male	19,7577329	10,6814261	33,6415818	2008	24,1674115	21,7623483	26,5724748	0	0,89563375	-1,52916143	3,32042893	1,03848583	0,93928784	1,14816011
Oceania and Ar	Female	15,6836527	7,86845301	28,8319936	2008	18,8196353	17,3699987	20,2692718	0,003	-0,15679541	-1,63835906	1,32476825	0,99173736	0,91668287	1,07293702
Australia	Male	22,2413748	21,0272508	23,5047378	2009	22,7709826	22,4814853	23,0604799	0	0	-0,409411	0,409411	1	0,98218116	1,01814211
Australia	Female	17,5208222	16,4892046	18,6026041	2009	18,4960623	18,2091858	18,7829387	0	0	-0,40570458	0,40570458	1	0,97830417	1,02217698
English speakin	Male	26,7475719	22,4365037	31,550048	2009	24,4917855	23,6950276	25,2885435	0	1,72080296	0,8730814	2,56852453	1,07556999	1,03865138	1,11380086
English speakin	Female	20,3100092	16,6190658	24,5790391	2009	18,7178999	18,1187623	19,3170376	0	0,22183765	-0,44243939	0,8861147	1,01199378	0,97663117	1,04863683
Europe (exclud	Male	15,0530252	10,459938	21,1857855	2009	21,3489132	19,6343644	23,0634621	0	-1,42206934	-3,16088689	0,31674821	0,93754906	0,86433308	1,01696702
Europe (exclud	Female	18,8839972	13,4597742	25,8414222	2009	17,4501608	16,4319536	18,468368	0,095	-1,04590148	-2,10375028	0,01194732	0,94345275	0,88817661	1,00216903
Asia	Male	21,510171	15,0179048	29,8240852	2009	19,8053839	18,6170657	20,9937021	0,011	-2,96559868	-4,18867216	-1,74252519	0,86976413	0,81802276	0,92477823
Asia	Female	7,90552551	3,69016585	16,1297311	2009	6,81739645	5,98263041	7,65216248	0	-11,6786658	-12,5613506	-10,795981	0,36858637	0,32578909	0,41700572
Middle East, Af	Male	28,89435	18,0556235	42,8381426	2009	26,164614	24,2516835	28,0775446	0,686	3,39363146	1,45891912	5,32834381	1,14903316	1,0668521	1,23754473
Middle East, Af	Female	21,3675168	12,3561308	34,3734304	2009	18,9846033	17,5066345	20,462572	0,21	0,48854098	-1,01701199	1,99409395	1,02641324	0,94808561	1,11121204
Oceania and Ar	Male	28,2606993	17,3542315	42,4969435	2009	23,2342431	20,9788669	25,4896193	0	0,46326052	-1,81061951	2,73714054	1,02034434	0,92518639	1,12528954
Oceania and Ar	Female	18,3140004	10,0425191	31,0469669	2009	18,3964186	17,037005	19,7558322	0,003	-0,0996437	-1,48899734	1,28970993	0,99461271	0,9222789	1,07261961
Australia	Male	22,0346734	20,8572636	23,2590155	2010	22,2701874	21,990792	22,5495828	0	0	-0,39512479	0,39512479	1	0,98241415	1,01790065
Australia	Female	17,8656951	16,8489981	18,9297748	2010	18,0156939	17,7388279	18,2925599	0	0	-0,39154771	0,39154771	1	0,97850077	1,0219716
English speakin	Male	27,6655797	23,4750717	32,2885055	2010	23,9488976	23,1799421	24,717853	0	1,67871016	0,86056944	2,49685088	1,07537926	1,03894028	1,11309626
English speakin	Female	20,2120195	16,5726251	24,4167267	2010	18,2089146	17,6306836	18,7871456	0	0,19322069	-0,44787672	0,8343181	1,01072513	0,97568978	1,04701855
Europe (exclud	Male	16,2056299	10,777113	23,6439254	2010	20,6334791	18,9787586	22,2881996	0	-1,63670831	-3,31485064	0,04143403	0,92650676	0,85427235	1,00484906
Europe (exclud	Female	15,933139	10,8778196	22,7384313	2010	17,2921779	16,3095005	18,2748553	0,095	-0,72351599	-1,74445165	0,29741967	0,95983968	0,90496573	1,018041
Asia	Male	15,3831641	10,0004128	22,9251856	2010	19,5048824	18,3580301	20,6517348	0,011	-2,76530497	-3,94569988	-1,58491005	0,87582929	0,82472454	0,93010079
Asia	Female	4,89635574	1,9114103	11,9737061	2010	6,3696753	5,56403803	7,17531256	0	-11,6460186	-12,4979025	-10,7941347	0,35356258	0,31126672	0,40160574
Middle East, Af	Male	28,3476388	17,5676885	42,3444638	2010	26,2334883	24,3873086	28,079668	0,686	3,96330091	2,09609944	5,83050238	1,17796442	1,09669756	1,26525327
Middle East, Af	Female	18,4796358	10,382767	30,7258652	2010	18,8158478	17,389452	20,2422435	0,21	0,80015386	-0,65286355	2,25317127	1,04441427	0,96667393	1,12840651
Oceania and Ar	Male	25,5836211	15,7482781	38,7372704	2010	22,3010747	20,1243988	24,4777506	0	0,03088728	-2,16364677	2,22542133	1,00138693	0,90753686	1,10494222
Oceania and Ar	Female	24,7132858	15,3967761	37,189141	2010	17,9732019	16,6612244	19,2851794	0,003	-0,042492	-1,38336479	1,2983808	0,99764139	0,92592883	1,07490804
Australia	Male	21,1364666	20,0892306	22,2231123	2011	21,7693922	21,4899968	22,0487877	0	0	-0,39512479	0,39512479	1	0,98201325	1,0183162
Australia	Female	17,3090443	16,3994512	18,2580697	2011	17,5353255	17,2584595	17,8121916	0	0	-0,39154771	0,39154771	1	0,97791837	1,02258024
English speakin	Male	22,8996309	19,7411453	26,3972508	2011	23,4060096	22,6370542	24,174965	0	1,63661736	0,81847664	2,45475808	1,07517975	1,03791822	1,11377897
English speakin	Female	19,3531521	16,3772603	22,7228525	2011	17,6999292	17,1216982	18,2781602	0	0,16460373	-0,47649368	0,80570114	1,00938698	0,97341887	1,04668412
Europe (exclud	Male	21,0795473	16,0201957	27,2188321	2011	19,918045	18,2633245	21,5727654	0	-1,85134728	-3,52948961	-0,17320494	0,91495641	0,84118738	0,99519471
Europe (exclud	Female	13,9583435	9,9249229	19,2800668	2011	17,134195	16,1515176	18,1168724	0,095	-0,4011305	-1,42206616	0,61980517	0,97712443	0,9206947	1,03701276
Asia	Male	18,0843156	13,1869639	24,2915197	2011	19,204381	18,0575286	20,3512333	0,011	-2,56501126	-3,74540617	-1,38461635	0,8821735	0,82990141	0,937738
Asia	Female	5,1071007	2,85284045	8,97801516	2011	5,92195414	5,11631688	6,72759141	0	-11,6133714	-12,4652553	-10,7614875	0,33771567	0,29449107	0,38728465
Middle East, Af	Male	31,1579003	22,3570892	41,5683536	2011	26,3023626	24,4561829	28,1485423	0,686	4,53297036	2,66576889	6,40017183	1,20822678	1,12501846	1,29758933
Middle East, Af	Female	16,3404883	9,89639667	25,7801107	2011	18,6470922	17,2206965	20,073488	0,21	1,11176674	-0,34125067	2,56478415	1,06340155	0,98350359	1,14979026
Oceania and Ar	Male	14,3076058	8,08330051	24,0696794	2011	21,3679063	19,1912304	23,5445822	0	-0,40148596	-2,59602001	1,7930481	0,98155732	0,88577989	1,08769095
Oceania and Ar	Female	15,2779602	8,98422242	24,7802644	2011	17,5499852	16,2380077	18,8619628	0,003	0,01465971	-1,32621309	1,3555325	1,00083601	0,9272147	1,08030289
Australia	Male	20,9167862	19,8693671	22,0042581	2012	21,2685971	20,9790998	21,5580944	0	0	-0,409411	0,409411	1	0,98093454	1,01943602
Australia	Female	16,6083382	15,7200274	17,5364012	2012	17,0549571	16,7680807	17,3418336	0	0	-0,40570458	0,40570458	1	0,97649263	1,02407327
English speakin	Male	20,9860383	17,9637776	24,3657481	2012	22,8631216	22,0663637	23,6598795	0	1,59452456	0,746803	2,44224613	1,07497084	1,03549605	1,11595047
English speakin	Female	16,5913177	13,8468765	19,754978	2012	17,1909439	16,5918063	17,7900815	0	0,13598676	-0,52829028	0,80026381	1,00797345	0,96971125	1,04774536
Europe (exclud	Male	22,6224843	16,8700387	29,6371266	2012	19,2026108	17,488062	20,9171597	0	-2,06598624	-3,8048038	-0,32716869	0,90286213	0,82489081	0,98820354
Europe (exclud	Female	17,6579202	12,6943632	24,028115	2012	16,9762121	15,9580049	17,9944194	0,095	-0,078745	-1,1365938	0,9791038	0,99538287	0,93526973	1,05935969
Asia	Male	19,9429987	14,5801842	26,6626412	2012	18,9038795	17,7155613	20,0921977	0,011	-2,36471755	-3,58779104	-1,14164407	0,88881648	0,83344934	0,94786173
Asia	Female	2,68599463	1,41592304	5,03710114	2012	5,47423299	4,63946696	6,30899902	0	-11,5807241	-12,4634089	-10,6980393	0,32097606	0,27532483	0,37419667
Middle East, Af	Male	25,8601605	17,2783878	36,8077217	2012	26,3712369	24,4583063	28,2841674	0,686	5,10263981	3,16792746	7,03735215	1,23991426	1,15169837	1,33488717
Middle East, Af	Female	17,5809191	10,5673727	27,8022638	2012	18,4783367	17,000368	19,9563055	0,21	1,42337962	-0,08217335	2,92893259	1,08345841	0,99842596	1,17573278
Oceania and Ar	Male	13,8486803	7,86814339	23,2288831	2012	20,4347379	18,1793617	22,690114	0	-0,83385919	-3,10773922	1,44002083	0,96079388	0,85967467	1,07380723
Oceania and Ar	Female	13,9819384	8,3275762	22,5319262	2012	17,1267685	15,7673549	18,4861822	0,003	0,07181141	-1,31754222	1,46116505	1,00421059	0,9259504	1,08908523
Australia	Male	20,1051624	19,0716163	21,1800605	2013	20,7678019	20,4590909	21,0765128	0	0	-0,4365832	0,4365832	1	0,97919731	1,02124464
Australia	Female	16,0978299	15,2211149	17,0149076	2013	16,5745887	16,2686726	16,8805049	0	0	-0,43263079	0,43263079	1	0,97423566	1,02644569
English speakin	Male	22,9288347	19,7807746	26,4129397	2013	22,3202336	21,4705957	23,1698716	0	1,55243176	0,64844776	2,45641577	1,07475186	1,03171712	1,11958165
English speakin	Female	16,4273489	13,6636099	19,6230518	2013	16,6819585	16,0430567	17,3208603	0	0,1073698	-0,60099465	0,81573426	1,00647798	0,96458506	1,05019035
Europe (exclud	Male	17,9145327	12,9640269	24,2292036	2013	18,4871767	16,6588349	20,3155184	0	-2,28062521	-4,13484636	-0,42640407	0,89018456	0,80546527	0,98381468
Europe (exclud	Female	14,5928142	10,3958246	20,1040424	2013	16,8182292	15,7324446	17,9040139	0,095	0,24364049	-0,88441668	1,37169766	1,01469964	0,94880324	1,08517269
Asia	Male	18,0102493	12,984561	24,4349024	2013	18,603378	17,3361924	19,8705637	0,011	-2,16442384	-3,4686715	-0,86017619	0,89577983	0,83545431	0,96046127
Asia	Female	3,00378474	1,68003287	5,31418771	2013	5,02651184	4,13634321	5,91668046	0	-11,5480769	-12,4893446	-10,6068092	0,30326616	0,25380263	0,36236962
Middle East, Af	Male	23,1820048	15,5185024	33,1451894	2013	26,4401111	24,4002213	28,480001	0,686	5,67230926	3,60919198	7,73542653	1,27312998	1,17692854	1,37719486
Middle East, Af	Female	17,2953979	10,6830363	26,7736868	2013	18,3095812	16,7335212	19,8856413	0,21	1,73499249	0,12951747	3,34046752	1,10467786	1,01158539	1,20633728
Oceania and Ar	Male	14,3566783	8,22813731	23,8629861	2013	19,5015695	17,0965062	21,9066327	0	-1,26623243	-3,69102761	1,15856275	0,93902906	0,82933747	1,06322891
Oceania and Ar	Female	14,6568939	8,80098649	23,4090273	2013	16,7035519	15,2539153	18,1531884	0,003	0,12896312	-1,35260054	1,61052677	1,00778077	0,92221546	1,10128504
Australia	Male	20,4788363	19,4537056	21,5435385	2014	20,2670067	19,9315323	20,6024811	0	0	-0,47443248	0,47443248	1	0,97686276	1,02368525
Australia	Female	15,9650975	15,0998009	16,870127	2014	16,0942203	15,761783	16,4266577	0	0	-0,47013741	0,47013741	1	0,97121097	1,02964241
English speakin	Male	22,0440173	18,947514	25,487438	2014	21,7773457	20,854049	22,7006424	0	1,51033896	0,52798469	2,49269323	1,07452205	1,02671269	1,12455769
English speakin	Female	16,2439724	13,5394899	19,3676581	2014	16,1729732	15,4786822	16,8672641	0	0,07875284	-0,69102278	0,84852846	1,00489324	0,95814262	1,05392496
Europe (exclud	Male	17,579354	12,447485	24,2411906	2014	17,7717425	15,7848939	19,7585911	0	-2,49526418	-4,51023575	-0,48029262	0,87688048	0,78317311	0,98180001
Europe (exclud	Female	16,4706467	11,7605691	22,5842935	2014	16,6602463	15,4803303	17,8401623	0,095	0,56602599	-0,65982732	1,7918793	1,03516952	0,96155093	1,11442452
Asia	Male	16,6597944	11,8913904	22,8445439	2014	18,3028766	16,9258331	19,67992	0,011	-1,96413014	-3,38144864	-0,54681163	0,90308731	0,83612943	0,97540722
Asia	Female	2,57192233	1,41085927	4,64347624	2014	4,57879068	3,6114495	5,54613186	0	-11,5154297	-12,5382999	-10,4925594	0,28449907	0,23008688	0,35177895
Middle East, Af	Male	25,1992328	17,1137657	35,4699914	2014	26,5089854	24,2922488	28,7257221	0,686	6,2419787	4,0000009	8,48395651	1,3079872	1,20110845	1,42437639
Middle East, Af	Female	15,1562342	8,97577522	24,4492925	2014	18,1408257	16,4281302	19,8535212	0,21	2,04660537	0,3019448	3,79126595	1,127164	1,02332841	1,24153562
Oceania and Ar	Male	19,5063499	12,5035335	29,1256495	2014	18,568401	15,9548325	21,1819696	0	-1,69860567	-4,33361679	0,93640546	0,91618863	0,79512453	1,05568571
Oceania and Ar	Female	16,582632	10,6062879	24,9852711	2014	16,2803352	14,7050234	17,855647	0,003	0,18611482	-1,42389197	1,79612161	1,01156408	0,91627015	1,11676876
Australia	Male	19,8243729	18,8093011	20,8801366	2015	19,7662115	19,3980667	20,1343564	0	0	-0,52063541	0,52063541	1	0,9740042	1,02668962
Australia	Female	15,3520298	14,5008477	16,243683	2015	15,613852	15,24904	15,978664	0	0	-0,51592206	0,51592206	1	0,96749736	1,03359456
English speakin	Male	20,1472198	17,1379725	23,5347834	2015	21,2344577	20,2212451	22,2476703	0	1,46824616	0,39022466	2,54626767	1,07428061	1,0206397	1,13074067
English speakin	Female	14,2509664	11,6475357	17,3222213	2015	15,6639878	14,9020829	16,4258928	0	0,05013587	-0,79460487	0,89487661	1,00321099	0,9505112	1,05883265
Europe (exclud	Male	18,6708444	13,2325231	25,6826536	2015	17,0563084	14,8759692	19,2366476	0	-2,70990315	-4,92110408	-0,49870222	0,86290225	0,75833094	0,98189359
Europe (exclud	Female	17,5710686	12,7317702	23,7491919	2015	16,5022634	15,2074405	17,7970863	0,095	0,88841148	-0,45682238	2,23364534	1,05689893	0,97381968	1,1470659
Asia	Male	16,1379427	11,7159488	21,8165053	2015	18,0023751	16,4912274	19,5135229	0,011	-1,76383643	-3,31918146	-0,2084914	0,91076507	0,83572698	0,99254067
Asia	Female	4,34633449	2,24934334	8,23361604	2015	4,13106953	3,06952318	5,19261588	0	-11,4827824	-12,6052656	-10,3602993	0,26457722	0,20440568	0,34246164
Middle East, Af	Male	21,5460633	14,1623217	31,3724474	2015	26,5778597	24,1452446	29,0104747	0,686	6,81164815	4,35133382	9,27196248	1,34461071	1,22470609	1,47625456
Middle East, Af	Female	15,5549259	9,0888464	25,3389994	2015	17,9720702	16,0925827	19,8515577	0,21	2,35821825	0,44365273	4,27278377	1,15103373	1,03407151	1,28122536
Oceania and Ar	Male	17,1303406	10,6138969	26,4631282	2015	17,6352326	14,76714	20,5033253	0	-2,1309789	-5,02260236	0,76064456	0,89219083	0,75746915	1,05087379
Oceania and Ar	Female	15,4765786	9,54610481	24,1093341	2015	15,8571185	14,1283939	17,5858431	0,003	0,24326653	-1,52353186	2,01006492	1,01558017	0,90843276	1,13536536
Australia	Male	20,4060628	19,4135978	21,4357688	2016	19,2654164	18,8601202	19,6707126	0	0	-0,5731754	0,5731754	1	0,9706867	1,03019852
Australia	Female	15,5070261	14,6762862	16,3757645	2016	15,1334836	14,7318565	15,5351106	0	0	-0,56798641	0,56798641	1	0,96316382	1,03824498
English speakin	Male	19,0568842	16,1373332	22,363781	2016	20,6915697	19,5761087	21,8070308	0	1,42615336	0,23934317	2,61296355	1,0740266	1,01363853	1,13801234
English speakin	Female	15,2293126	12,5768865	18,3238794	2016	15,1550025	14,3162098	15,9937952	0	0,02151891	-0,90846896	0,95150678	1,00142194	0,9418018	1,06481629
Europe (exclud	Male	18,7457415	13,3499818	25,6761177	2016	16,3408742	13,9405058	18,7412427	0	-2,92454212	-5,35888669	-0,49019755	0,84819731	0,73122482	0,98388163
Europe (exclud	Female	15,6504214	11,1810086	21,4744513	2016	16,3442805	14,9187904	17,7697707	0,095	1,21079697	-0,27019135	2,69178529	1,08000782	0,98590375	1,18309408
Asia	Male	18,5123846	13,8408585	24,3156519	2016	17,7018736	16,0382282	19,3655191	0,011	-1,56354272	-3,2758456	0,14876016	0,918842	0,83447855	1,01173435
Asia	Female	3,92263872	2,41986731	6,29841507	2016	3,68334838	2,51467593	4,85202082	0	-11,4501352	-12,6858939	-10,2143765	0,24339065	0,17702207	0,33464192
Middle East, Af	Male	25,0499135	16,9849485	35,3152714	2016	26,646734	23,9686312	29,3248367	0,686	7,3813176	4,67272032	10,0899149	1,38313823	1,24816296	1,53270961
Middle East, Af	Female	17,3306009	10,6206736	26,9991736	2016	17,8033147	15,7341584	19,872471	0,21	2,66983113	0,56205697	4,77760529	1,17641881	1,04420967	1,32536716
Oceania and Ar	Male	14,4877137	8,49527018	23,616211	2016	16,7020642	13,5445376	19,8595909	0	-2,56335214	-5,74678417	0,62007989	0,86694541	0,71677286	1,04858091
Oceania and Ar	Female	13,474143	8,0215382	21,7564297	2016	15,4339018	13,5307227	17,3370809	0,003	0,30041823	-1,64467686	2,24551332	1,01985123	0,89899451	1,15695537
Australia	Male	19,3965444	18,4171991	20,4149354	2017	18,7646212	18,3188115	19,2104309	0	0	-0,63047015	0,63047015	1	0,9669593	1,03416969
Australia	Female	14,9303201	14,108508	15,7912013	2017	14,6531152	14,2113414	15,094889	0	0	-0,62476247	0,62476247	1	0,95825933	1,04355884
English speakin	Male	19,3819888	16,4635818	22,6772882	2017	20,1486818	18,9217189	21,3756446	0	1,38406056	0,07861654	2,68950459	1,07375905	1,0058166	1,14629097
English speakin	Female	14,0891911	11,5613409	17,06311	2017	14,6460171	13,7233785	15,5686557	0	-0,00709805	-1,03004773	1,01585163	0,99951559	0,93209259	1,07181564
Europe (exclud	Male	13,1589374	8,56762614	19,6810729	2017	15,6254401	12,9851303	18,2657499	0	-3,13918109	-5,81686334	-0,46149884	0,83270746	0,70207856	0,98764121
Europe (exclud	Female	17,6945403	12,6900534	24,1272311	2017	16,1862977	14,6183152	17,7542801	0,095	1,53318247	-0,0958458	3,16221074	1,10463184	0,99805988	1,22258347
Asia	Male	20,1520949	15,2578554	26,132133	2017	17,4013722	15,5714283	19,231316	0,011	-1,36324901	-3,24671411	0,52021608	0,92735004	0,83257216	1,03291719
Asia	Female	3,67460194	1,95579617	6,79918734	2017	3,23562722	1,95013399	4,52112045	0	-11,417488	-12,7767734	-10,0582025	0,22081497	0,14824843	0,32890231
Middle East, Af	Male	24,9066553	16,5968757	35,6010437	2017	26,7156082	23,7698017	29,6614148	0,686	7,95098705	4,97163772	10,9303364	1,42372223	1,27185812	1,59371941
Middle East, Af	Female	16,9007399	10,2152869	26,6622383	2017	17,6345592	15,3585696	19,9105488	0,21	2,98144401	0,66297623	5,29991178	1,20346827	1,05408059	1,37402765
Oceania and Ar	Male	19,561375	12,5932434	29,1014315	2017	15,7688958	12,295742	19,2420496	0	-2,99572538	-6,49737405	0,5059233	0,84035247	0,67336688	1,04874817
Oceania and Ar	Female	13,5075651	8,15547758	21,5479535	2017	15,0106851	12,9172638	17,1041064	0,003	0,35756994	-1,78195733	2,4970972	1,02440232	0,88818582	1,18150964
Australia	Male	18,2367667	17,2827525	19,2311992	2018	18,263826	17,7749759	18,7526762	0	0	-0,69133852	0,69133852	1	0,96285458	1,03857843
Australia	Female	14,516974	13,7009718	15,3729185	2018	14,1727468	13,6883222	14,6571714	0	0	-0,68507979	0,68507979	1	0,95281185	1,04952515
English speakin	Male	20,3116175	17,1895418	23,8375153	2018	19,6057938	18,2603745	20,951213	0	1,34196776	-0,08950958	2,77344511	1,07347681	0,99724778	1,15553276
English speakin	Female	14,4526157	11,8448304	17,5204301	2018	14,1370318	13,1253176	15,148746	0	-0,03571502	-1,15742477	1,08599473	0,99748002	0,9214273	1,07980998
Europe (exclud	Male	12,7614074	8,3612389	18,9971595	2018	14,910006	12,014789	17,8052229	0	-3,35382006	-6,29001755	-0,41762256	0,81636816	0,6710542	0,99314924
Europe (exclud	Female	17,30428	12,3585663	23,6941118	2018	16,0283148	14,3089523	17,7476772	0,095	1,85556796	0,06926612	3,6418698	1,13092508	1,0105066	1,2656934
Asia	Male	19,0253899	14,2723652	24,9015021	2018	17,1008707	15,094256	19,1074854	0,011	-1,16295531	-3,22825842	0,90234781	0,93632466	0,83015159	1,05607685
Asia	Female	3,92084046	2,0345752	7,42335878	2018	2,78790607	1,37830566	4,19750648	0	-11,3848407	-12,8753576	-9,89432388	0,19670894	0,11850541	0,32652019
Middle East, Af	Male	27,62937	19,0878963	38,1890092	2018	26,7844825	23,5542748	30,0146902	0,686	8,52065649	5,25366766	11,7876453	1,46653185	1,29610787	1,65936471
Middle East, Af	Female	18,6366649	11,7102626	28,3446512	2018	17,4658037	14,97008	19,9615274	0,21	3,29305688	0,75075401	5,83535976	1,23235135	1,06396332	1,4273893
Oceania and Ar	Male	17,8789997	11,1317609	27,4525563	2018	14,8357274	11,0272601	18,6441948	0	-3,42809861	-7,26781188	0,41161466	0,81230118	0,62751676	1,05149893
Oceania and Ar	Female	16,1054208	9,88743184	25,1427201	2018	14,5874684	12,291939	16,8829979	0,003	0,41472164	-1,93136502	2,7608083	1,02926191	0,87617415	1,20909762
Australia	Male	17,6819317	16,7482326	18,6560191	2019	17,7630308	17,2292242	18,2968375	0	0	-0,75491658	0,75491658	1	0,95839113	1,04341534
Australia	Female	14,4915019	13,6871096	15,3347693	2019	13,6923784	13,1634044	14,2213525	0	0	-0,74808228	0,74808228	1	0,94683073	1,05615498
English speakin	Male	19,3635251	16,2474507	22,9137228	2019	19,0629058	17,5937568	20,5320548	0	1,29987496	-0,26324637	2,86299629	1,07317867	0,98797754	1,16572737
English speakin	Female	14,1137965	11,4568836	17,2667064	2019	13,6280464	12,5232912	14,7328016	0	-0,06433198	-1,28919833	1,16053437	0,99530162	0,90981937	1,08881538
Europe (exclud	Male	18,5054992	12,8212861	25,9593624	2019	14,1945718	11,0330999	17,3560437	0	-3,56845903	-6,77468017	-0,36223788	0,79910754	0,63826482	1,00048261
Europe (exclud	Female	16,3253862	11,4744214	22,701257	2019	15,8703319	13,9928505	17,7478132	0,095	2,17795345	0,22737665	4,12853026	1,15906319	1,02343286	1,31266792
Asia	Male	15,4222353	11,5879158	20,2349145	2019	16,8003693	14,6092188	18,9915197	0,011	-0,9626616	-3,21789767	1,29257448	0,94580533	0,82732417	1,08125419
Asia	Female	3,94845013	2,37005453	6,50794642	2019	2,34018492	0,8009524	3,87941743	0	-11,3521935	-12,9797838	-9,72460317	0,1709115	0,08843542	0,33030588
Middle East, Af	Male	31,0862202	22,4267928	41,3087265	2019	26,8533568	23,3260872	30,3806264	0,686	9,09032594	5,52289265	12,6577592	1,51175534	1,32117912	1,72982163
Middle East, Af	Female	20,0799174	12,6203785	30,4139076	2019	17,2970482	14,5718084	20,0222879	0,21	3,60466976	0,82856725	6,38077227	1,26326104	1,07409027	1,48574891
Oceania and Ar	Male	18,4954948	11,7598378	27,8705836	2019	13,902559	9,74385082	18,0612672	0	-3,86047185	-8,05329944	0,33235574	0,78266818	0,57944487	1,05716611
Oceania and Ar	Female	16,558146	10,3374915	25,4592236	2019	14,1642518	11,6576169	16,6708866	0,003	0,47187335	-2,08996817	3,03371486	1,03446248	0,86307397	1,23988518

Subpopulation	Sex	Prevalence(P)	P_LCI	P_UCI	Year	Linear(lr)	lr_LCI	lr_UCI	p_value	Difference(D)	D_LCI	D_UCI	Ratio(R)	R_LCI	R_UCI
Australia	Male	29,1516483	27,7995107	30,5417324	2002	29,0308736	28,4928638	29,5688834	0,013	0	-0,76086076	0,76086076	1	0,97413179	1,02655514
Australia	Female	23,7605595	22,5823152	24,980443	2002	24,2992616	23,7623984	24,8361249	0,014	0	-0,75923927	0,75923927	1	0,96923773	1,03173862
English speakin	Male	36,4526979	32,6273545	40,457216	2002	36,0036562	34,7363331	37,2709794	0,204	6,97278261	5,59598832	8,3495769	1,24018508	1,19181847	1,2905145
English speakin	Female	32,2608225	28,5961014	36,1573734	2002	32,6294315	31,6987604	33,5601027	0,005	8,33016987	7,25575331	9,40458643	1,34281576	1,29523236	1,39214724
Europe (exclud	Male	43,9604071	37,7790858	50,3349297	2002	45,4810632	42,601736	48,3603904	0,144	16,4501896	13,5210293	19,3793498	1,56664466	1,46663572	1,67347314
Europe (exclud	Female	30,7262364	25,4021286	36,6184711	2002	32,1587033	29,3346666	34,98274	0,552	7,85944164	4,98482771	10,7340556	1,32344364	1,20886856	1,448878
Asia	Male	31,7413191	24,6071648	39,8505499	2002	31,6008409	29,8868996	33,3147822	0,089	2,56996729	0,77356831	4,36636628	1,08852532	1,02788971	1,15273784
Asia	Female	6,81203649	3,73163875	12,1152357	2002	8,44583279	6,82990484	10,0617607	0,009	-15,8534289	-17,5562046	-14,1506531	0,3475757	0,28668459	0,42139993
Middle East, Af	Male	27,5158912	18,2815542	39,1783828	2002	22,0189684	17,9006354	26,1373013	0,597	-7,01190527	-11,1652318	-2,85857878	0,7584673	0,62850759	0,91529944
Middle East, Af	Female	10,6113437	5,07437771	20,8622181	2002	9,1883303	5,56849597	12,8081646	0,039	-15,1109313	-18,7703606	-11,4515021	0,37813208	0,2548473	0,56105703
Oceania and Ar	Male	11,1169851	4,94892406	23,1039718	2002	21,471052	17,1835525	25,7585516	0,05	-7,55982162	-11,880945	-3,23869827	0,73959373	0,60519786	0,90383478
Oceania and Ar	Female	21,6736374	12,9098309	34,0600295	2002	21,897509	18,8037012	24,9913168	0,071	-2,40175266	-5,54179547	0,73829015	0,90115944	0,78108136	1,03969749
Australia	Male	28,6273832	27,2650724	30,0296595	2003	28,9594924	28,4667931	29,4521918	0,013	0	-0,69678208	0,69678208	1	0,97622657	1,02435237
Australia	Female	23,8083783	22,6201276	25,0388506	2003	24,3696544	23,8780051	24,8613038	0,014	0	-0,69529716	0,69529716	1	0,9718719	1,02894218
English speakin	Male	36,4034191	32,4602381	40,5381018	2003	36,0831296	34,9225387	37,2437205	0,204	7,12363717	5,86279466	8,38447969	1,24598626	1,20146369	1,2921587
English speakin	Female	32,6337195	28,8131375	36,6997305	2003	32,7710247	31,9187334	33,6233159	0,005	8,40137023	7,41743959	9,38530087	1,3447472	1,30120522	1,38974623
Europe (exclud	Male	44,3968757	37,6398379	51,3677349	2003	45,2714179	42,6345838	47,908252	0,144	16,3119255	13,6294552	18,9943958	1,56326697	1,47123013	1,66106142
Europe (exclud	Female	31,6670401	25,837196	38,1358967	2003	32,0775123	29,4913123	34,6637124	0,552	7,70785791	5,07534011	10,3403757	1,31628918	1,21131582	1,43035958
Asia	Male	35,8321652	27,9126925	44,6081537	2003	31,4538616	29,8842661	33,0234571	0,089	2,49436917	0,8492605	4,13947784	1,08613304	1,03035319	1,14493263
Asia	Female	10,4053794	6,24471741	16,8402362	2003	8,67535413	7,19551747	10,1551908	0,009	-15,6943003	-17,2536706	-14,13493	0,35599004	0,29980565	0,42270352
Middle East, Af	Male	23,8495625	14,7206596	36,2342918	2003	22,1240776	18,3525851	25,8955701	0,597	-6,83541483	-10,6389537	-3,03187593	0,76396635	0,64368328	0,90672633
Middle East, Af	Female	9,82798812	4,5605098	19,9102745	2003	9,57447168	6,25949495	12,8894484	0,039	-14,7951827	-18,1464198	-11,4439457	0,392885	0,27774374	0,55575914
Oceania and Ar	Male	24,0137687	14,106878	37,8149916	2003	21,9022459	17,9758338	25,828658	0,05	-7,05724652	-11,0144507	-3,10004239	0,75630628	0,63167251	0,90553123
Oceania and Ar	Female	18,9860162	10,6489897	31,5456671	2003	22,1806116	19,3473602	25,0138631	0,071	-2,18904279	-5,0646354	0,68654982	0,91017342	0,79976366	1,03582556
Australia	Male	28,9014025	27,5117479	30,3318788	2004	28,8881112	28,4387912	29,3374312	0,013	0	-0,63543444	0,63543444	1	0,97824376	1,0222401
Australia	Female	23,843755	22,6418194	25,088802	2004	24,4400472	23,9916848	24,8884097	0,014	0	-0,63408025	0,63408025	1	0,97438935	1,0262838
English speakin	Male	36,2441363	32,2750762	40,4100569	2004	36,162603	35,1041954	37,2210105	0,204	7,27449174	6,12465913	8,42432435	1,25181611	1,21100572	1,29400178
English speakin	Female	32,4379763	28,6008663	36,5265185	2004	32,9126178	32,1353658	33,6898698	0,005	8,47257059	7,57526937	9,36987181	1,34666752	1,3069929	1,38754649
Europe (exclud	Male	43,5831247	36,6027018	50,8274894	2004	45,0617726	42,6570965	47,4664487	0,144	16,1736614	13,7273671	18,6199556	1,55987258	1,47553353	1,64903231
Europe (exclud	Female	32,4732571	26,3507961	39,2599398	2004	31,9963214	29,6378213	34,3548215	0,552	7,55627418	5,15553438	9,95701397	1,30917592	1,21341374	1,41249562
Asia	Male	30,1598591	22,8108282	38,6897988	2004	31,3068823	29,8754806	32,7382839	0,089	2,41877105	0,9185047	3,91903739	1,08372894	1,0326342	1,13735185
Asia	Female	9,6005104	5,6628841	15,8170581	2004	8,90487547	7,55532989	10,2544211	0,009	-15,5351717	-16,9572484	-14,113095	0,3643559	0,31277169	0,4244477
Middle East, Af	Male	25,8276445	16,3254015	38,327298	2004	22,2291868	18,7897525	25,6686212	0,597	-6,65892439	-10,1275836	-3,19026514	0,76949257	0,65867183	0,89895875
Middle East, Af	Female	6,18996651	2,23501162	15,9981707	2004	9,96061305	6,9375009	12,9837252	0,039	-14,4794342	-17,5356141	-11,4232542	0,40755294	0,30069915	0,55237734
Oceania and Ar	Male	26,7577221	15,7882895	41,584982	2004	22,3334398	18,7527256	25,914154	0,05	-6,55467143	-10,1634666	-2,94587629	0,77310142	0,65808088	0,90822545
Oceania and Ar	Female	18,6661578	10,3106518	31,4206617	2004	22,4637143	19,8799143	25,0475143	0,071	-1,97633292	-4,59874619	0,64608035	0,91913547	0,81807879	1,03267561
Australia	Male	28,2500705	26,8775805	29,6642138	2005	28,81673	28,4082425	29,2252175	0,013	0	-0,57768855	0,57768855	1	0,98015262	1,02024928
Australia	Female	24,2774021	23,0748701	25,5218112	2005	24,51044	24,102823	24,9180569	0,014	0	-0,57645743	0,57645743	1	0,97675556	1,0237976
English speakin	Male	34,6999888	30,7629855	38,858066	2005	36,2420763	35,2798529	37,2042998	0,204	7,42534631	6,38000616	8,47068647	1,25767484	1,22038638	1,29610264
English speakin	Female	32,2824341	28,4333439	36,3876547	2005	33,0542109	32,3475927	33,7608292	0,005	8,54377095	7,72801309	9,35952882	1,34857681	1,31254153	1,38560142
Europe (exclud	Male	38,9271579	32,0314971	46,2961583	2005	44,8521273	42,6659792	47,0382755	0,144	16,0353973	13,8114131	18,2593816	1,55646138	1,47942603	1,63750804
Europe (exclud	Female	26,3674061	20,9048021	32,6678806	2005	31,9151304	29,7709619	34,0592989	0,552	7,40469045	5,22212084	9,58726005	1,30210353	1,21503138	1,39541549
Asia	Male	29,1301196	21,8206561	37,7071416	2005	31,159903	29,8585817	32,4612242	0,089	2,34317292	0,97924513	3,70710071	1,08131294	1,03466026	1,13006918
Asia	Female	9,89616344	6,36790521	15,0647895	2005	9,13439682	7,90749285	10,3613008	0,009	-15,3760432	-16,6688869	-14,0831994	0,37267372	0,32549944	0,42668491
Middle East, Af	Male	21,6008471	12,9338145	33,8197359	2005	22,3342961	19,2074246	25,4611676	0,597	-6,48243395	-9,63587452	-3,32899339	0,77504616	0,67330853	0,89215647
Middle East, Af	Female	8,70515408	3,57741409	19,6824959	2005	10,3467544	7,59837132	13,0951375	0,039	-14,1636856	-16,9421314	-11,3852397	0,42213663	0,32349426	0,55085778
Oceania and Ar	Male	20,6701109	10,9633669	35,5405052	2005	22,7646337	19,5093214	26,0199461	0,05	-6,05209633	-9,33293777	-2,7712549	0,78997977	0,68423935	0,91206101
Oceania and Ar	Female	21,5827345	12,678803	34,2845505	2005	22,7468169	20,3978229	25,0958109	0,071	-1,76362305	-4,14772128	0,62047518	0,92804605	0,83587903	1,03037573
Australia	Male	28,8160192	27,4519973	30,2195846	2006	28,7453488	28,3743053	29,1163924	0,013	0	-0,52473486	0,52473486	1	0,98191101	1,01842223
Australia	Female	24,280525	23,0892359	25,5128897	2006	24,5808328	24,2105799	24,9510856	0,014	0	-0,52361659	0,52361659	1	0,97892346	1,02153033
English speakin	Male	37,130833	33,0354725	41,419862	2006	36,3215497	35,4475282	37,1955712	0,204	7,57620088	6,62668159	8,52572017	1,26356267	1,22952558	1,29854201
English speakin	Female	34,6466385	30,6828811	38,835568	2006	33,1958041	32,5539579	33,8376503	0,005	8,61497131	7,8739897	9,35595292	1,35047516	1,31777757	1,38398406
Europe (exclud	Male	48,4477253	40,8746355	56,0927818	2006	44,6424821	42,6567266	46,6282375	0,144	15,8971332	13,87701	17,9172565	1,55303323	1,48274282	1,62665581
Europe (exclud	Female	31,8436077	25,4728187	38,9744904	2006	31,8339395	29,8863157	33,7815632	0,552	7,25310672	5,27060186	9,23561157	1,29507164	1,21598975	1,37929663
Asia	Male	28,621077	21,4607092	37,0436841	2006	31,0129236	29,8308877	32,1949596	0,089	2,2675748	1,02867114	3,50647846	1,07888493	1,03633175	1,12318539
Asia	Female	8,13325968	5,34268164	12,1936442	2006	9,36391816	8,24947806	10,4783582	0,009	-15,2169146	-16,3912501	-14,0425791	0,38094389	0,3378792	0,42949744
Middle East, Af	Male	20,7347548	12,1904292	33,0160852	2006	22,4394053	19,5991578	25,2796529	0,597	-6,30594351	-9,17032467	-3,44156236	0,78062734	0,68736628	0,88654196
Middle East, Af	Female	11,3188443	5,41301918	22,1586636	2006	10,7328958	8,23644261	13,229349	0,039	-13,847937	-16,3716972	-11,3241767	0,43663679	0,34585382	0,55124932
Oceania and Ar	Male	22,9922144	12,8435434	37,6920904	2006	23,1958276	20,2389127	26,1527425	0,05	-5,54952124	-8,52962509	-2,56941739	0,80694194	0,70989972	0,91724969
Oceania and Ar	Female	19,9013973	11,3236512	32,5889147	2006	23,0299196	20,8962456	25,1635936	0,071	-1,55091318	-3,71647359	0,61464723	0,93690559	0,85296469	1,02910718
Australia	Male	30,0002162	28,5971348	31,4418245	2007	28,6739676	28,3358517	29,0120836	0,013	0	-0,47816814	0,47816814	1	0,98346224	1,01681586
Australia	Female	26,0265904	24,7933699	27,2988857	2007	24,6512255	24,3138302	24,9886209	0,014	0	-0,4771491	0,4771491	1	0,98083013	1,01954454
English speakin	Male	39,3362471	34,9661678	43,8839494	2007	36,4010231	35,6045652	37,197481	0,204	7,72705545	6,86179968	8,59231122	1,26947981	1,23831547	1,30142846
English speakin	Female	34,9235616	30,8082926	39,2764965	2007	33,3373972	32,7525106	33,9222839	0,005	8,68617167	8,01094725	9,3613961	1,35236267	1,32260272	1,38279226
Europe (exclud	Male	42,7779232	35,351139	50,5450941	2007	44,4328368	42,6233039	46,2423696	0,144	15,7588691	13,9180184	17,5997199	1,54958802	1,48526204	1,61669993
Europe (exclud	Female	38,8829405	31,9176568	46,3337839	2007	31,7527485	29,9779634	33,5275337	0,552	7,10152298	5,29495224	8,90809373	1,28807992	1,21604942	1,36437701
Asia	Male	32,6214105	25,100117	41,1580108	2007	30,8659443	29,7888062	31,9430824	0,089	2,19197668	1,06301747	3,32093589	1,07644483	1,03751468	1,11683575
Asia	Female	8,67009915	5,63702328	13,1084542	2007	9,5934395	8,57789855	10,6089804	0,009	-15,057786	-16,1279071	-13,987665	0,38916684	0,34976767	0,43300407
Middle East, Af	Male	16,0362649	8,81710898	27,3906639	2007	22,5445146	19,9563201	25,132709	0,597	-6,12945307	-8,73963946	-3,51926668	0,78623631	0,70053875	0,88241734
Middle East, Af	Female	4,39033729	1,36077438	13,2581892	2007	11,1190372	8,84412759	13,3939468	0,039	-13,5321884	-15,8319817	-11,232395	0,45105413	0,36743049	0,55370971
Oceania and Ar	Male	26,5819843	15,8426471	41,0502493	2007	23,6270215	20,9325131	26,3215299	0,05	-5,04694615	-7,76258562	-2,33130668	0,82398857	0,73473152	0,92408878
Oceania and Ar	Female	25,8497477	16,0269132	38,9037867	2007	23,3130222	21,3686976	25,2573469	0,071	-1,33820331	-3,31158465	0,63517803	0,94571453	0,8690706	1,02911774
Australia	Male	29,1147095	27,7153776	30,5548283	2008	28,6025864	28,2914447	28,9137282	0,013	0	-0,44002084	0,44002084	1	0,98473378	1,01550289
Australia	Female	25,0201363	23,7876106	26,2944905	2008	24,7216183	24,4111397	25,032097	0,014	0	-0,4390831	0,4390831	1	0,9823957	1,01791976
English speakin	Male	33,6201687	29,5036442	38,0014422	2008	36,4804965	35,7475784	37,2134145	0,204	7,87791002	7,08168263	8,67413741	1,27542649	1,24661764	1,3049011
English speakin	Female	34,1912484	30,0200765	38,6221229	2008	33,4789904	32,9407648	34,0172159	0,005	8,75737204	8,13601567	9,3787284	1,35423943	1,32689209	1,3821504
Europe (exclud	Male	48,1122026	39,4651431	56,8738447	2008	44,2231915	42,5580196	45,8883634	0,144	15,6206051	13,9266137	17,3145964	1,54612561	1,48669947	1,60792713
Europe (exclud	Female	33,040494	25,7634022	41,2314386	2008	31,6715576	30,0383613	33,3047539	0,552	6,94993925	5,28749313	8,61238538	1,28112801	1,2149063	1,3509593
Asia	Male	29,7624868	22,4402677	38,2942778	2008	30,718965	29,7277588	31,7101712	0,089	2,11637856	1,07748547	3,15527164	1,07399256	1,03803742	1,1111931
Asia	Female	7,92896052	4,82308946	12,7666065	2008	9,82296084	8,88843772	10,757484	0,009	-14,8986575	-15,8834064	-13,9139086	0,39734295	0,3609855	0,43736223
Middle East, Af	Male	13,2615386	6,66119327	24,6731422	2008	22,6496238	20,2679103	25,0313373	0,597	-5,95296263	-8,35491355	-3,55101172	0,79187328	0,71243277	0,88017187
Middle East, Af	Female	12,0686153	5,6806852	23,825273	2008	11,5051786	9,41175673	13,5986004	0,039	-13,2164398	-15,3327601	-11,1001194	0,46538938	0,38779885	0,55850417
Oceania and Ar	Male	32,5927955	20,5499596	47,4758515	2008	24,0582154	21,5786695	26,5377612	0,05	-4,54437105	-7,04336221	-2,0453799	0,84112028	0,75831417	0,93296861
Oceania and Ar	Female	24,4014522	14,45008	38,1499434	2008	23,5961249	21,8069146	25,3853352	0,071	-1,12549344	-2,94144234	0,69045547	0,95447331	0,88386135	1,03072648
Australia	Male	28,4536334	27,0826156	29,8656296	2009	28,5312052	28,2394285	28,822982	0,013	0	-0,41263468	0,41263468	1	0,98564151	1,01456766
Australia	Female	25,4205899	24,1955513	26,6858182	2009	24,7920111	24,5008561	25,0831661	0,014	0	-0,41175531	0,41175531	1	0,98352877	1,01674707
English speakin	Male	36,6595747	32,4216769	41,1143988	2009	36,5599698	35,8726674	37,2472723	0,204	8,02876459	7,28209305	8,77543613	1,28140292	1,25427114	1,30912161
English speakin	Female	33,2005479	29,1286383	37,5401623	2009	33,6205835	33,1158562	34,1253108	0,005	8,8285724	8,24588821	9,41125659	1,35610554	1,33050261	1,38220114
Europe (exclud	Male	50,7295127	42,5139838	58,9058305	2009	44,0135462	42,4520118	45,5750806	0,144	15,482341	13,8937808	17,0709011	1,54264588	1,48672552	1,60066958
Europe (exclud	Female	27,397089	21,2386962	34,557676	2009	31,5903666	30,0588177	33,1219155	0,552	6,79835552	5,23937723	8,35733381	1,27421557	1,2122124	1,33939013
Asia	Male	30,6715587	23,3357042	39,1362806	2009	30,5719857	29,6424705	31,5015009	0,089	2,04078043	1,0665463	3,01501457	1,07152801	1,03770104	1,10645768
Asia	Female	7,31950319	4,67053078	11,2929078	2009	10,0524822	9,17612221	10,9288422	0,009	-14,7395289	-15,6629887	-13,8160691	0,40547264	0,37132863	0,44275622
Middle East, Af	Male	19,7471426	11,1771534	32,4849188	2009	22,7547331	20,5212534	24,9882127	0,597	-5,77647219	-8,02892974	-3,52401465	0,79753844	0,72259162	0,88025871
Middle East, Af	Female	12,426081	5,80916463	24,6106785	2009	11,8913199	9,92818915	13,8544507	0,039	-12,9006912	-14,8852953	-10,916087	0,47964322	0,4064805	0,56597455
Oceania and Ar	Male	19,513877	10,7707818	32,7492876	2009	24,4894093	22,1641862	26,8146324	0,05	-4,04179596	-6,38525412	-1,69833779	0,85833771	0,78016104	0,94434813
Oceania and Ar	Female	31,6796817	20,8084939	45,0028486	2009	23,8792275	22,2013747	25,5570804	0,071	-0,91278356	-2,61571087	0,79014375	0,96318235	0,89695344	1,03430144
Australia	Male	28,349468	27,0165055	29,7214148	2010	28,459824	28,1782287	28,7414194	0,013	0	-0,39823599	0,39823599	1	0,98610453	1,01409128
Australia	Female	25,6510465	24,4469966	26,8932892	2010	24,8624039	24,5814086	25,1433991	0,014	0	-0,3973873	0,3973873	1	0,9841436	1,01611188
English speakin	Male	35,3484989	31,2967972	39,6222281	2010	36,6394432	35,9761238	37,3027626	0,204	8,17961916	7,45900237	8,90023595	1,28740934	1,2611204	1,31424628
English speakin	Female	33,3696291	29,3356054	37,6627227	2010	33,7621766	33,2750616	34,2492917	0,005	8,89977276	8,33742106	9,46212446	1,35796107	1,3332997	1,3830786
Europe (exclud	Male	46,3565613	38,1277093	54,7887392	2010	43,8039009	42,2968555	45,3109463	0,144	15,3440769	13,8109488	16,877205	1,53914869	1,48502345	1,59524665
Europe (exclud	Female	33,5110621	26,5873202	41,2253392	2010	31,5091757	30,0310694	32,9872819	0,552	6,64677179	5,14219332	8,15135027	1,26734228	1,20764163	1,32999428
Asia	Male	29,2575273	22,1637113	37,5271574	2010	30,4250064	29,5279262	31,3220865	0,089	1,96518231	1,02494362	2,90542101	1,06905111	1,0363144	1,10282195
Asia	Female	10,3028491	6,59987016	15,7334719	2010	10,2820035	9,43622372	11,1277833	0,009	-14,5804004	-15,4716365	-13,6891642	0,41355629	0,38060518	0,44936016
Middle East, Af	Male	22,8425746	13,3157848	36,3287938	2010	22,8598423	20,7042989	25,0153857	0,597	-5,59998175	-7,77384085	-3,42612265	0,80323203	0,7305751	0,88311482
Middle East, Af	Female	14,434334	7,24685893	26,6984661	2010	12,2774613	10,3828331	14,1720896	0,039	-12,5849426	-14,5002949	-10,6695902	0,49381634	0,42302563	0,57645344
Oceania and Ar	Male	24,018268	14,6579227	36,779922	2010	24,9206032	22,6765176	27,1646887	0,05	-3,53922086	-5,80090516	-1,27753656	0,87564151	0,79980296	0,95867118
Oceania and Ar	Female	28,5122971	18,6993362	40,8851851	2010	24,1623302	22,5430252	25,7816352	0,071	-0,70007369	-2,34357816	0,94343077	0,97184208	0,90798627	1,04018866
Australia	Male	28,1162295	26,9229243	29,3411894	2011	28,3884428	28,1068475	28,6700382	0,013	0	-0,39823599	0,39823599	1	0,98606983	1,01412696
Australia	Female	25,0867703	24,0162692	26,1885419	2011	24,9327967	24,6518014	25,2137919	0,014	0	-0,3973873	0,3973873	1	0,98418801	1,01606603
English speakin	Male	36,5840631	33,1780672	40,1297287	2011	36,7189166	36,0555972	37,382236	0,204	8,33047373	7,60985694	9,05109052	1,29344596	1,26706215	1,32037915
English speakin	Female	32,2680599	28,8989687	35,8319834	2011	33,9037698	33,4166547	34,3908849	0,005	8,97097312	8,40862142	9,53332482	1,35980613	1,33520085	1,38486485
Europe (exclud	Male	42,5097083	36,0664783	49,2178824	2011	43,5942557	42,0872102	45,1013011	0,144	15,2058128	13,6726847	16,7389409	1,53563392	1,48138651	1,59186782
Europe (exclud	Female	36,0689889	30,119516	42,4792728	2011	31,4279847	29,9498785	32,906091	0,552	6,49518806	4,99060958	7,99976654	1,2605078	1,20099653	1,32296795
Asia	Male	30,2237739	23,8178799	37,5043787	2011	30,278027	29,3809469	31,1751072	0,089	1,88958419	0,94934549	2,82982288	1,06656174	1,03375279	1,10041197
Asia	Female	12,8029193	8,75144053	18,3527848	2011	10,5115249	9,66574506	11,3573047	0,009	-14,4212718	-15,3125079	-13,5300357	0,4215943	0,38869535	0,45727779
Middle East, Af	Male	27,0334137	18,6796755	37,4046487	2011	22,9649515	20,8094081	25,120495	0,597	-5,42349131	-7,59735041	-3,24963221	0,80895425	0,73609366	0,88902678
Middle East, Af	Female	14,0279346	7,93151112	23,6086853	2011	12,6636027	10,7689744	14,558231	0,039	-12,269194	-14,1845463	-10,3538416	0,50790944	0,43714597	0,59012781
Oceania and Ar	Male	28,1584437	19,0672228	39,470351	2011	25,3517971	23,1077115	27,5958826	0,05	-3,03664577	-5,29833007	-0,77496147	0,89303232	0,81692796	0,97622651
Oceania and Ar	Female	28,7734821	20,205826	39,1897659	2011	24,4454328	22,8261279	26,0647378	0,071	-0,48736382	-2,13086829	1,15614064	0,9804529	0,91673744	1,04859674
Australia	Male	27,7566546	26,5693547	28,9760748	2012	28,3170617	28,0252849	28,6088384	0,013	0	-0,41263468	0,41263468	1	0,98553371	1,01467863
Australia	Female	24,7053326	23,6467981	25,7952433	2012	25,0031894	24,7120345	25,2943444	0,014	0	-0,41175531	0,41175531	1	0,98366675	1,01660446
English speakin	Male	36,3302034	32,9338433	39,8686037	2012	36,7983899	36,1110875	37,4856924	0,204	8,4813283	7,73465676	9,22799984	1,29951301	1,27208639	1,32753095
English speakin	Female	33,7358846	30,344065	37,303795	2012	34,0453629	33,5406356	34,5500902	0,005	9,04217348	8,45948929	9,62485767	1,3616408	1,33621209	1,38755344
Europe (exclud	Male	42,70233	35,9112776	49,7801034	2012	43,3846104	41,8230759	44,9461448	0,144	15,0675487	13,4789885	16,6561089	1,53210142	1,47580205	1,59054852
Europe (exclud	Female	30,9711969	25,2271461	37,369485	2012	31,3467938	29,8152449	32,8783427	0,552	6,34360433	4,78462604	7,90258262	1,25371181	1,19229718	1,31828987
Asia	Male	28,9701869	22,737911	36,1122595	2012	30,1310477	29,2015325	31,0605629	0,089	1,81398607	0,83975193	2,7882202	1,06405983	1,03000867	1,09923669
Asia	Female	12,3924225	8,50734018	17,7083234	2012	10,7410462	9,86468623	11,6174062	0,009	-14,2621432	-15,185603	-13,3386834	0,42958704	0,39560165	0,46649206
Middle East, Af	Male	24,1334219	15,7878061	35,0542539	2012	23,0700608	20,8365811	25,3035404	0,597	-5,24700087	-7,49945842	-2,99454332	0,81470532	0,7391248	0,89801446
Middle East, Af	Female	18,6334544	11,2339249	29,2982013	2012	13,0497441	11,0866133	15,0128749	0,039	-11,9534454	-13,9380495	-9,96884118	0,52192318	0,4488263	0,60692478
Oceania and Ar	Male	26,4949112	17,7573116	37,5679664	2012	25,782991	23,4577679	28,1082141	0,05	-2,53407068	-4,87752884	-0,19061251	0,91051082	0,83150281	0,99702604
Oceania and Ar	Female	24,8402038	17,1103544	34,6042754	2012	24,7285355	23,0506826	26,4063883	0,071	-0,27465395	-1,97758126	1,42827336	0,98901524	0,92321937	1,05950025
Australia	Male	28,4345139	27,2337597	29,6666258	2013	28,2456805	27,9345387	28,5568222	0,013	0	-0,44002084	0,44002084	1	0,98454237	1,01570032
Australia	Female	25,3506707	24,2820269	26,4499163	2013	25,0735822	24,7631036	25,3840609	0,014	0	-0,4390831	0,4390831	1	0,98264066	1,01766601
English speakin	Male	37,2457972	33,8252964	40,798935	2013	36,8778633	36,1449453	37,6107814	0,204	8,63218287	7,83595548	9,42841025	1,30561072	1,2762781	1,3356175
English speakin	Female	34,5915905	31,1332343	38,2208999	2013	34,1869561	33,6487305	34,7251816	0,005	9,11337384	8,49201748	9,7347302	1,36346517	1,33642696	1,39105042
Europe (exclud	Male	42,6545652	36,0776359	49,5019727	2013	43,1749651	41,5097932	44,840137	0,144	14,9292846	13,2352933	16,623276	1,52855107	1,46845381	1,59110784
Europe (exclud	Female	31,1082383	25,5469965	37,2740118	2013	31,2656028	29,6324065	32,8987991	0,552	6,1920206	4,52957447	7,85446673	1,24695397	1,18177783	1,31572463
Asia	Male	29,1642185	22,7735385	36,5006291	2013	29,9840684	28,9928622	30,9752746	0,089	1,73838794	0,69949486	2,77728103	1,06154527	1,02519299	1,09918656
Asia	Female	12,8326628	8,99127644	17,9907925	2013	10,9705675	10,0360444	11,9050907	0,009	-14,1030147	-15,0877636	-13,1182658	0,43753491	0,40144745	0,4768664
Middle East, Af	Male	29,0225926	20,1700555	39,822307	2013	23,17517	20,7934566	25,5568835	0,597	-5,07051043	-7,47246134	-2,66855952	0,82048546	0,73991659	0,9098274
Middle East, Af	Female	13,5586091	7,68845907	22,8034831	2013	13,4358855	11,3424636	15,5293073	0,039	-11,6376968	-13,7540171	-9,52137638	0,53585823	0,45832113	0,62651277
Oceania and Ar	Male	31,3555061	22,1529719	42,3034675	2013	26,2141849	23,734639	28,6937307	0,05	-2,03149558	-4,53048674	0,46749558	0,92807765	0,84377696	1,02080072
Oceania and Ar	Female	22,6886567	15,3901753	32,1337902	2013	25,0116381	23,2224279	26,8008484	0,071	-0,06194408	-1,87789298	1,75400482	0,99752951	0,92767626	1,07264265
Australia	Male	28,016938	26,8386704	29,2262695	2014	28,1742993	27,8361833	28,5124152	0,013	0	-0,47816814	0,47816814	1	0,98317142	1,01711662
Australia	Female	25,2695971	24,2088866	26,3606183	2014	25,143975	24,8065796	25,4813704	0,014	0	-0,4771491	0,4771491	1	0,98120225	1,01915788
English speakin	Male	36,1662927	32,7575202	39,7202513	2014	36,9573367	36,1608788	37,7537946	0,204	8,78303744	7,91778167	9,64829321	1,31173934	1,27977863	1,34449823
English speakin	Female	34,2353075	30,8267483	37,8147231	2014	34,3285492	33,7436626	34,9134358	0,005	9,1845742	8,50934978	9,85979862	1,36527932	1,33598861	1,39521222
Europe (exclud	Male	44,3984883	37,3878048	51,6395508	2014	42,9653198	41,155787	44,7748526	0,144	14,7910206	12,9501698	16,6318713	1,52498273	1,45964101	1,59324952
Europe (exclud	Female	31,6708699	25,7261099	38,2813187	2014	31,1844119	29,4096267	32,959197	0,552	6,04043687	4,23386612	7,84700762	1,24023397	1,16979321	1,31491641
Asia	Male	30,9017716	24,4722084	38,1669373	2014	29,8370891	28,759951	30,9142272	0,089	1,66278982	0,53383062	2,79174903	1,05901797	1,01948636	1,10008245
Asia	Female	13,9359584	9,65820932	19,6953238	2014	11,2000889	10,1845479	12,2156298	0,009	-13,9438861	-15,0140072	-12,8737651	0,44543828	0,40642467	0,48819689
Middle East, Af	Male	19,554685	12,5570007	29,1519288	2014	23,2802793	20,6920848	25,8684737	0,597	-4,89401999	-7,50420638	-2,2838336	0,82629488	0,73887626	0,92405626
Middle East, Af	Female	21,6498793	13,9063341	32,0977368	2014	13,8220268	11,5471172	16,0969364	0,039	-11,3219482	-13,6217415	-9,02215484	0,54971526	0,46603863	0,64841593
Oceania and Ar	Male	22,5021048	14,8656149	32,5611003	2014	26,6453788	23,9508704	29,3398871	0,05	-1,52892049	-4,24455996	1,18671898	0,9457335	0,85416674	1,04711623
Oceania and Ar	Female	23,6046404	16,4578386	32,6423114	2014	25,2947408	23,3504162	27,2390654	0,071	0,15076579	-1,82261555	2,12414713	1,0059961	0,93048344	1,08763693
Australia	Male	27,841737	26,6637554	29,0511451	2015	28,1029181	27,7318745	28,4739616	0,013	0	-0,52473486	0,52473486	1	0,98150134	1,01884731
Australia	Female	25,5302116	24,468588	26,6216616	2015	25,2143678	24,8441149	25,5846206	0,014	0	-0,52361659	0,52361659	1	0,97944754	1,02098372
English speakin	Male	38,5894015	35,0481501	42,2556816	2015	37,0368101	36,1627886	37,9108315	0,204	8,93389201	7,98437272	9,88341129	1,31789909	1,2827392	1,3540227
English speakin	Female	35,4618877	31,9429062	39,1455553	2015	34,4701423	33,8282961	35,1119886	0,005	9,25577456	8,51479295	9,99675617	1,36708335	1,335046	1,3998895
Europe (exclud	Male	40,9177338	34,1135923	48,0884757	2015	42,7556745	40,7699191	44,7414299	0,144	14,6527565	12,6326332	16,6728797	1,52139626	1,44968175	1,59665843
Europe (exclud	Female	28,6466858	23,1255438	34,8877663	2015	31,1032209	29,1555971	33,0508447	0,552	5,88885314	3,90634828	7,871358	1,23355149	1,15671098	1,31549653
Asia	Male	32,2058288	25,5231159	39,7054495	2015	29,6901098	28,5080738	30,8721457	0,089	1,5871917	0,34828804	2,82609536	1,05647783	1,01308077	1,10173387
Asia	Female	11,5194801	7,90002203	16,5001326	2015	11,4296102	10,3151701	12,5440503	0,009	-13,7847575	-14,9590931	-12,610422	0,45329751	0,41073347	0,50027245
Middle East, Af	Male	27,6829358	19,2263185	38,1044604	2015	23,3853885	20,545141	26,225636	0,597	-4,71752955	-7,5819107	-1,8531484	0,83213382	0,73643708	0,94026593
Middle East, Af	Female	18,4946976	10,9002094	29,6213821	2015	14,2081682	11,711715	16,7046214	0,039	-11,0061996	-13,5299598	-8,48243932	0,56349492	0,47240671	0,67214652
Oceania and Ar	Male	29,5122426	20,8813442	39,9109919	2015	27,0765727	24,1196578	30,0334875	0,05	-1,02634539	-4,00644924	1,95375846	0,96347905	0,86311665	1,07551148
Oceania and Ar	Female	26,1232659	18,3487465	35,7496354	2015	25,5778434	23,4441694	27,7115174	0,071	0,36347566	-1,80208475	2,52903607	1,01441542	0,93203128	1,10408166
Australia	Male	27,3369146	26,1976138	28,5066244	2016	28,0315369	27,6230494	28,4400244	0,013	0	-0,57768855	0,57768855	1	0,97960238	1,02082234
Australia	Female	25,0948836	24,0605272	26,1583895	2016	25,2847606	24,8771436	25,6923775	0,014	0	-0,57645743	0,57645743	1	0,97745931	1,02306049
English speakin	Male	37,7849496	34,2777384	41,4248267	2016	37,1162834	36,15406	38,0785069	0,204	9,08474658	8,03940642	10,1300867	1,32409021	1,28529219	1,36405939
English speakin	Female	33,0319677	29,6122658	36,6410084	2016	34,6117355	33,9051173	35,3183537	0,005	9,32697492	8,51121706	10,1427328	1,36887733	1,33372768	1,40495332
Europe (exclud	Male	41,3507384	34,6581488	48,3788297	2016	42,5460292	40,3598811	44,7321774	0,144	14,5144924	12,2905081	16,7384766	1,51779153	1,43885372	1,60105999
Europe (exclud	Female	30,2418037	24,4661656	36,7180905	2016	31,02203	28,8778615	33,1661984	0,552	5,73726941	3,5546998	7,91983901	1,22690622	1,14284752	1,3171476
Asia	Male	31,1203131	24,8598039	38,1568121	2016	29,5431304	28,2418092	30,8444517	0,089	1,51159358	0,14766579	2,87552137	1,05392475	1,00614375	1,10397483
Asia	Female	12,1437619	8,55224212	16,9637572	2016	11,6591316	10,4322276	12,8860355	0,009	-13,625629	-14,9184727	-12,3327852	0,46111299	0,41454613	0,5129108
Middle East, Af	Male	19,8929839	12,5910881	29,9771813	2016	23,4904977	20,3636263	26,6173692	0,597	-4,54103911	-7,69447967	-1,38759855	0,83800249	0,73297648	0,95807737
Middle East, Af	Female	12,8548129	6,93443262	22,6022283	2016	14,5943096	11,8459265	17,3426927	0,039	-10,690451	-13,4688968	-7,91200512	0,57719786	0,47779298	0,69728393
Oceania and Ar	Male	26,0269643	17,9440536	36,1469309	2016	27,5077666	24,2524542	30,7630789	0,05	-0,5237703	-3,80461173	2,75707114	0,98131496	0,87101401	1,10558389
Oceania and Ar	Female	26,9592738	19,2181216	36,4130585	2016	25,8609461	23,5119521	28,2099401	0,071	0,57618553	-1,8079127	2,96028377	1,02278786	0,93265574	1,12163037
Australia	Male	27,3627985	26,2184094	28,537818	2017	27,9601557	27,5108357	28,4094757	0,013	0	-0,63543444	0,63543444	1	0,97752987	1,02298664
Australia	Female	25,110601	24,0753461	26,1750254	2017	25,3551533	24,9067909	25,8035158	0,014	0	-0,63408025	0,63408025	1	0,97530216	1,02532327
English speakin	Male	38,3213533	34,8285934	41,9389778	2017	37,1957568	36,1373492	38,2541644	0,204	9,23560114	8,08576853	10,3854338	1,33031294	1,28754193	1,37450476
English speakin	Female	36,1831551	32,682221	39,8371814	2017	34,7533286	33,9760767	35,5305806	0,005	9,39817528	8,50087406	10,2954765	1,37066135	1,3321341	1,41030286
Europe (exclud	Male	43,7280818	36,5803016	51,1461587	2017	42,336384	39,9317079	44,7410601	0,144	14,3762283	11,929934	16,8225226	1,51416839	1,42737563	1,60623866
Europe (exclud	Female	31,9110366	25,8815896	38,613349	2017	30,940839	28,5823389	33,2993391	0,552	5,58568568	3,18494588	7,98642547	1,22029785	1,12844954	1,319622
Asia	Male	27,5787808	21,72641	34,3164504	2017	29,3961511	27,9647495	30,8275527	0,089	1,43599545	-0,06427089	2,9362618	1,05135864	0,99880725	1,10667497
Asia	Female	11,2264191	8,06661796	15,416399	2017	11,8886529	10,5391073	13,2381985	0,009	-13,4665004	-14,8885771	-12,0444237	0,46888507	0,41799655	0,52596896
Middle East, Af	Male	26,0823059	17,6556062	36,7366063	2017	23,595607	20,1561727	27,0350413	0,597	-4,36454867	-7,83320792	-0,89588942	0,84390113	0,72879051	0,97719319
Middle East, Af	Female	12,795575	7,01571482	22,2002713	2017	14,980451	11,9573388	18,0035631	0,039	-10,3747024	-13,4308823	-7,3185224	0,5908247	0,48248135	0,72349704
Oceania and Ar	Male	24,4063787	16,7576688	34,1154243	2017	27,9389605	24,3582463	31,5196746	0,05	-0,0211952	-3,62999035	3,58759994	0,99924195	0,87816222	1,13701598
Oceania and Ar	Female	24,8235801	17,3060045	34,2540394	2017	26,1440487	23,5602487	28,7278487	0,071	0,7888954	-1,83351787	3,41130868	1,03111381	0,93261824	1,14001168
Australia	Male	28,262754	27,1065398	29,448362	2018	27,8887745	27,3960751	28,3814738	0,013	0	-0,69678208	0,69678208	1	0,9753252	1,02529904
Australia	Female	24,8610948	23,8205827	25,9315863	2018	25,4255461	24,9338968	25,9171954	0,014	0	-0,69529716	0,69529716	1	0,97302413	1,02772374
English speakin	Male	36,8619938	33,3172943	40,5544632	2018	37,2752302	36,1146393	38,4358211	0,204	9,38645571	8,12561319	10,6472982	1,33656752	1,28956656	1,38528153
English speakin	Female	34,4893802	30,9891463	38,1663177	2018	34,8949218	34,0426305	35,747213	0,005	9,46937564	8,48544501	10,4533063	1,37243549	1,33033998	1,41586301
Europe (exclud	Male	42,1410664	35,171842	49,4381216	2018	42,1267387	39,4899046	44,7635728	0,144	14,2379642	11,5554939	16,9204345	1,51052671	1,41541125	1,61203391
Europe (exclud	Female	29,7022183	23,885233	36,2609702	2018	30,8596481	28,273448	33,4458481	0,552	5,43410195	2,80158415	8,06661974	1,21372607	1,11370007	1,32273581
Asia	Male	29,4878083	23,6821302	36,0445494	2018	29,2491718	27,6795763	30,8187673	0,089	1,36039733	-0,28471134	3,005506	1,04877939	0,99117006	1,10973711
Asia	Female	10,9362659	7,97178711	14,8255542	2018	12,1181743	10,6383376	13,5980109	0,009	-13,3073719	-14,8667421	-11,7480016	0,47661412	0,42118349	0,5393398
Middle East, Af	Male	24,8775583	16,8668051	35,0871679	2018	23,7007162	19,9292238	27,4722087	0,597	-4,18805823	-7,99159713	-0,38451933	0,84982997	0,72409944	0,99739198
Middle East, Af	Female	11,3968977	5,95372276	20,7200882	2018	15,3665923	12,0516156	18,6815691	0,039	-10,0589538	-13,4101908	-6,70771671	0,6043761	0,48667939	0,75053613
Oceania and Ar	Male	28,9441073	20,1819938	39,6219589	2018	28,3701544	24,4437423	32,2965664	0,05	0,48137989	-3,47582424	4,43858402	1,0172607	0,88478645	1,16956961
Oceania and Ar	Female	22,9708197	15,6119024	32,4640559	2018	26,4271514	23,5938999	29,2604028	0,071	1,00160528	-1,87398733	3,87719789	1,03939366	0,93211207	1,15902284
Australia	Male	28,8413108	27,6756531	30,0356753	2019	27,8173933	27,2793835	28,3554031	0,013	0	-0,76086076	0,76086076	1	0,9730187	1,02772948
Australia	Female	25,0586602	24,0200012	26,1267882	2019	25,4959389	24,9590757	26,0328021	0,014	0	-0,75923927	0,75923927	1	0,97066019	1,03022666
English speakin	Male	36,445619	32,8493079	40,1999489	2019	37,3547035	36,0873804	38,6220267	0,204	9,53731028	8,16051599	10,9141046	1,34285421	1,29142333	1,39633331
English speakin	Female	35,0497067	31,4698627	38,8061782	2019	35,0365149	34,1058437	35,9671861	0,005	9,54057601	8,46615944	10,6149926	1,37419983	1,3283998	1,42157893
Europe (exclud	Male	38,8892065	31,9182582	46,3462085	2019	41,9170934	39,0377662	44,7964206	0,144	14,0997001	11,1705399	17,0288604	1,50686633	1,40308071	1,61832896
Europe (exclud	Female	30,2042721	24,180965	36,9958413	2019	30,7784571	27,9544204	33,6024938	0,552	5,28251822	2,40790429	8,15713214	1,20719057	1,09873218	1,32635515
Asia	Male	28,8770955	23,4411831	34,997331	2019	29,1021925	27,3882512	30,8161338	0,089	1,28479921	-0,51159978	3,08119819	1,0461869	0,98330462	1,1130905
Asia	Female	10,283104	7,66246912	13,667357	2019	12,3476956	10,7317676	13,9636235	0,009	-13,1482433	-14,8510191	-11,4454675	0,48430049	0,42417816	0,55294445
Middle East, Af	Male	22,7270265	15,4417537	32,1428785	2019	23,8058255	19,6874925	27,9241584	0,597	-4,01156779	-8,16489428	0,14175871	0,85578923	0,71906336	1,01851274
Middle East, Af	Female	11,0850515	5,95377672	19,7118043	2019	15,7527337	12,1328994	19,3725681	0,039	-9,74320517	-13,4026345	-6,08377588	0,61785266	0,49053489	0,77821562
Oceania and Ar	Male	26,7932177	18,9246676	36,4620342	2019	28,8013482	24,5138487	33,0888478	0,05	0,98395498	-3,33716837	5,30507834	1,03537193	0,8910498	1,20306973
Oceania and Ar	Female	25,4328229	17,7182325	35,0745399	2019	26,710254	23,6164462	29,8040618	0,071	1,21431515	-1,92572766	4,35435795	1,04762779	0,93127697	1,1785151

The content has been provided by the author(s) and has not been reviewed, verified, or endorsed by European Publishing. It may not have undergone peer review. The views, opinions, and recommendations expressed are solely those of the author(s) and do not necessarily reflect the position of European Publishing. European Publishing accepts no responsibility or liability for any consequences arising from the use of, or reliance on, this content.

© 2026 Aung L. et al. DOI:10.18332/tid/217085
